# Synthesis of 1-[bis(trifluoromethyl)phosphine]-1’-oxazolinylferrocene ligands and their application in regio- and enantioselective Pd-catalyzed allylic alkylation of monosubstituted allyl substrates

**DOI:** 10.3762/bjoc.10.126

**Published:** 2014-05-30

**Authors:** Zeng-Wei Lai, Rong-Fei Yang, Ke-Yin Ye, Hongbin Sun, Shu-Li You

**Affiliations:** 1 State Key Laboratory of Organometallic Chemistry, Shanghai Institute of Organic Chemistry, Chinese Academy of Sciences, 345 Lingling Lu, Shanghai 200032, China; 2 State Key Laboratory of Natural Medicines and Center of Drug Discovery, China Pharmaceutical University, 24 Tongjia Xiang, Nanjing 210009, China; 3 Process Development and Manufacturing Department, Pharmaron (Beijing) Co. Ltd, 6 Taihe Road, BDA, Beijing, 100176, China

**Keywords:** allylic substitution, enantioselectivity, ferrocene, organophosphorus, palladium, regioselectivity

## Abstract

A class of novel, easily accessible and air-stable 1-[bis(trifluoromethyl)phosphine]-1’-oxazolinylferrocene ligands has been synthesized from ferrocene. It became apparent that these ligands can be used in the regio- and enantioselective Pd-catalyzed allylic alkylation of monosubstituted allyl substrates in a highly efficient manner. Excellent regio- and enantioselectivity could be obtained for a wide range of substrates.

## Introduction

**Scheme 1:**
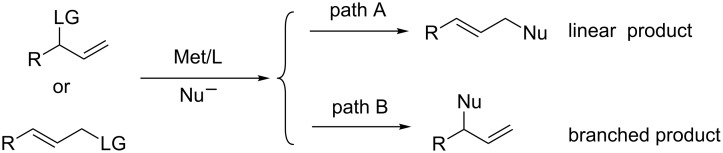
Transition metal-catalyzed allylic substitution reactions with monosubstituted allyl substrates.

**Figure 1:**
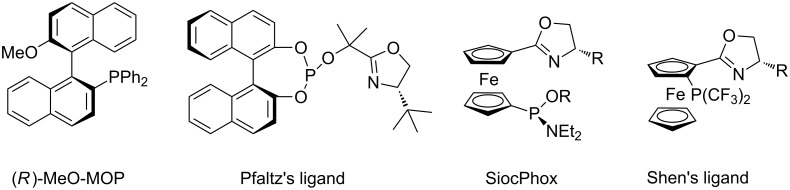
Representative ligands developed for the regio- and enantioselective Pd-catalyzed allylic alkylation.

The palladium-catalyzed asymmetric allylic alkylation (AAA) reaction is now becoming an efficient method for the construction of carbon–carbon bonds [1–5]. Despite extensive investigation and noteworthy advances in this field, several challenges remain to be solved. For instance, with monosubstituted allyl substrates, the palladium-catalyzed allylic substitution reaction prefers to give linear products rather than the branched ones [6–9] (Scheme 1). Accordingly, the regio- and enantioselective allylic substitution reaction of monosubstituted allylic substrates to preferably obtain the branched products is one of the continuing challenges. To our knowledge, there are several cases in which high levels of both regio- and enantioselectivity have been realized by introducing special ligands [10–34] (Figure 1). Hayashi and coworkers reported a sterically bulky chiral monophosphine ligand (MeO-MOP) could be used for the Pd-catalyzed alkylation of branched monosubstituted allyl acetate favoring the branched products. However, linear products were favored when the linear allyl substrates were employed [23–24]. The chiral oxazoline–phosphite ligands introduced by Pfaltz and coworkers proved to be highly efficient for regio- and enantiocontrol in the Pd-catalyzed allylic alkylation reaction. Excellent results were obtained for the bulky and electron-rich aryl allyl substrates [25–27]. In 2001, Dai, Hou and their coworkers synthesized a new class of 1,1’-ferrocene-based *P*,*N*-ligands, namely SiocPhox. The application of these SiocPhox ligands in the Pd-catalyzed allylic substitution led to excellent regio- and enantioselectivities for a wide range of substrates in both allylic alkylation and amination reactions despite of the electronic properties of the allylic substrates [28–33]. Recently, Shen and co-workers reported an elegant synthesis of bis(perfluoroalkyl)phosphine-oxazoline ligands where small but strongly electron-withdrawing substituents were introduced at the phosphorus [34]. 1,2-Ferrocene based *P*,*N*-ligands were synthesized and gave excellent regio- and enantioselectivities in the Pd-catalyzed allylic alkylation reactions of monosubstituted allylic substrates. Inspired by these pioneering studies above and as our continuing interests in the transition metal-catalyzed asymmetric allylic alkylation reaction [35–38], we envisaged that the 1-[bis(trifluoromethyl)phosphine]-1’-oxazolinylferrocene ligands, a straightforward combination of the features of SiocPhox and Shen’s ligand, should be highly efficient for the Pd-catalyzed allylic alkylation reactions of monosubstituted allyl substrates. Herein, we report the synthesis of 1-[bis(trifluoromethyl)phosphine]-1’-oxazolinylferrocene ligands and their application in Pd-catalyzed allylic alkylation reactions of monosubstituted allyl substrates with excellent regio- and enantioselectivity.

## Results and Discussion

**Scheme 2:**
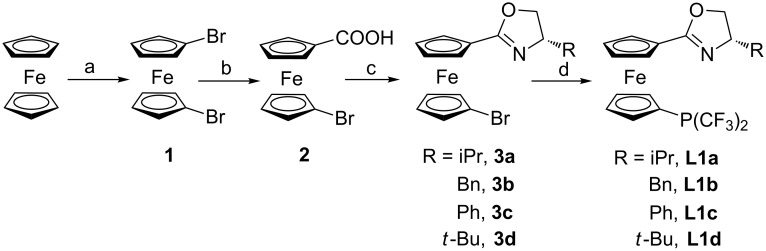
Preparation of 1-[bis(trifluoromethyl)phosphine]-1’-oxazolinylferrocene ligands. Reagents and conditions: (a) (i) *n*-BuLi, TMEDA, Et_2_O, rt; (ii) (BrCF_2_)_2_, −78 °C. (b) *n*-BuLi, CO_2_, THF, −20 °C. (c) (i) (COCl)_2_, DCM, rt; then TEA, amino alcohol, DCM, rt; (ii) Ph_3_P, CCl_4_, TEA, CH_3_CN, rt. (d) (i) *n*-BuLi, TMEDA, P(OPh)_3_, Et_2_O, −78 °C; (ii) TMSCF_3_, CsF, Et_2_O, rt.

As depicted in Scheme 2, ligands **L1a–L1d** were synthesized from known compounds **3**, which were obtained from ferrocene in three steps according to the reported procedures [39–41]. The commercially available ferrocene was dilithiated with *n*-BuLi and then quenched with dibromoterafluoroethane to give dibromoferrocence **1**. Treatment of **1** with *n*-BuLi at −20 °C followed by trapping with CO_2_ afforded compound **2**. Treatment of compound **2** with (COCl)_2_ and then chiral amino alcohols yielded the amide intermediates which were transformed to their corresponding 1-bromo-1’-oxazolinylferrocenes **3**. Eventually, lithium–bromide exchange of **3** with *n*-BuLi at −78 °C, followed by quenching with P(OPh)_3_, provided the phosphonite intermediates which were used without further purification. Subsequently, trifluoromethylation provided the ligands **L1a–d** in moderate yields, upon treatment with Ruppert’s reagent (TMSCF_3_) and CsF [42–45]. Notably, ligands **L1a–d** are moisture and air-stable, and their NMR spectra show no change even after being stored over six months under ambient atmosphere.

To test the suitability of these 1-[bis(trifluoromethyl)phosphine]-1’-oxazolinylferrocene ligands in Pd-catalyzed allylic alkylation reactions, we began our study by choosing methyl cinnamyl carbonate and dimethyl malonate as the model substrates, along with the catalysts derived from Pd_2_(dba)_3_ and ligands **1a–d**. The results are summarized in Table 1. Ligands **L1a–d** were screened in the reaction using bis(trimethylsilyl)acetamide (BSA) as the base and LiOAc as the additive. The results suggested that ligands **L1a–d** were effective for this reaction with full conversion and high selectivities (entries 1–4, Table 1). The catalyst derived from **L1d** gave the highest selectivities [b/l (branched/linear): 95/5, 82% ee; entry 4, Table 1]. With ligand **L1d**, different reaction parameters including the Pd precursor and solvent were further optimized. The utilization of [Pd(C_3_H_5_)Cl]_2_ as Pd precursor or DCM as solvent resulted in slightly lower selectivities (entries 5–6, Table 1). Further screening of the additives revealed that NaOAc was the optimal one (b/l: 97/3, 85% ee, entry 7, Table 1). Running the reaction at 0 °C resulted in an increased enantioselectivity (b/l: 96/4, 88% ee, entry 9, Table 1). When the reaction was run at −30 °C, only a trace amount of product was formed. As for the leaving groups of allyl substrates, the cinnamyl acetate could also be tolerated to give a similar level of regio- and enantioselectivity (entry 11, Table 1). The absolute configuration of the product was assigned as (*S*) by comparing the sign of the optical rotation with that reported in literature [28].

**Table 1:** Evaluation of the ligands and optimization of the reaction conditions.^a^

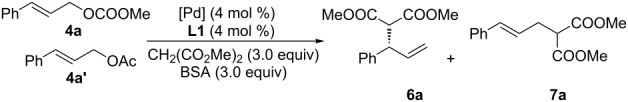									

entry	**4** or **4a’**	[Pd]	**L1**	Additive	Solvent	*T* (°C)	Yield (%)^b^	**6a**/**7a**^c^	ee (%)^d^

1	**4a**	Pd_2_(dba)_3_	**L1a**	LiOAc	DCE	rt	95	84/16	68
2	**4a**	Pd_2_(dba)_3_	**L1b**	LiOAc	DCE	rt	96	93/7	68
3	**4a**	Pd_2_(dba)_3_	**L1c**	LiOAc	DCE	rt	91	85/15	80
4	**4a**	Pd_2_(dba)_3_	**L1d**	LiOAc	DCE	rt	95	95/5	82
5	**4a**	[Pd(C_3_H_5_)Cl]_2_	**L1d**	LiOAc	DCE	rt	93	90/10	76
6	**4a**	Pd_2_(dba)_3_	**L1d**	LiOAc	DCM	rt	91	83/17	76
7	**4a**	Pd_2_(dba)_3_	**L1d**	NaOAc	DCE	rt	90	97/3	85
8	**4a**	Pd_2_(dba)_3_	**L1d**	KOAc	DCE	rt	91	91/9	82
9	**4a**	**Pd_2_(dba)_3_**	**L1d**	**NaOAc**	**DCE**	**0**	**95**	**96/4**	**88**
10^e^	**4a**	Pd_2_(dba)_3_	**L1d**	NaOAc	DCE	−30	trace	nd	nd
11^e^	**4a’**	Pd_2_(dba)_3_	**L1d**	NaOAc	DCE	rt	80	95/5	87

^a^Reagents and conditions: 2.0 mol % Pd_2_(dba)_3_, 4.0 mol % ligand, 0.2 mmol allyl substrate, 0.6 mmol dimethyl malonate, 0.6 mmol BSA, 3.0 mol % additive, solvent (2 mL). ^b^Isolated yield after 12 h. ^c^Determined by ^1^H NMR of the crude reaction mixture. ^d^Determined by HPLC. ^e^Reaction for 24 h.

Under the optimized reaction conditions (2 mol % of Pd_2_(dba)_3_, 4 mol % of **L1d**, 300 mol % of CH_2_(COOMe)_2_, 300 mol % of BSA and 3 mol % of NaOAc in DCE at 0 °C; entry 9, Table 1), the substrate scope was examined to test the generality of the reaction (Table 2). We first compared the reaction of branched substrate **5** with the linear substrate **4a**. Nearly identical results were obtained indicating that the reaction proceeds via the formation of the same Pd-π-allyl intermediate. Substrates bearing either an electron-donating group or electron-withdrawing group on the aromatic ring of the aryl allyl carbonates all proceeded smoothly in full conversion within 12 h. In all cases, the reactions gave excellent regioselectivity favoring the formation of the branched products in good to excellent enantioselectivity (b/l: 93/7–99/1, 81–94% ee). It is known that the regioselectivity could be strongly influenced by electronic properties of the allyl substrates and the formation of branched products was dramatically reduced for substrates bearing electron-withdrawing groups [21]. Fortunately, with our catalytic system, substrates bearing electron-withdrawing groups were well tolerated with excellent regioselectivity and preferred formation of the branched products (b/l: 93/7–99/1, entries 8–10, and 13, Table 2). Reactions of sterically hindered 1-naphthyl allyl carbonate, 2-MeO and 2-Me-substituted cinnamyl carbonates occurred smoothly to give excellent regio- and enantioselectivity (b/l: up to 99/1, up to 94% ee, entries 3, 11, 12, Table 2). In addition, heteroaryl allyl carbonates **4e** and **4f** also gave good regioselectivity with slightly lower enantioselectivity (entries 6 and 7, Table 2). Good regioselectivity (b/l: 81/19) was obtained with 2-buten-3-yl carbonate as a substrate (entry 14, Table 2).

**Table 2:** Regio- and enantioselective allylic alkylation of monosubstituted allyl substrates.^a^

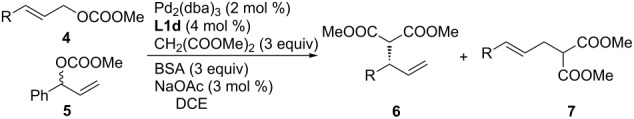					

entry	R	*T* (°C)	Yield (%)^b^	**6**/**7**^c^	ee (%)^d^

1	**4a**, Ph	0	95	96/4	88
2	**5**	0	93	95/5	87
3	**4b**, 1-naphthyl	0	95	99/1	92
4	**4c**, 4-MeC_6_H_4_	0	93	95/5	85
5	**4d**, 4-MeOC_6_H_4_	0	96	95/5	82
6	**4e**, 2-thienyl	0	94	98/2	70
7	**4f**, 2-furyl	rt	90	83/17	65
8	**4g**, 4-ClC_6_H_4_	0	91	96/4	83
9	**4h**, 4-BrC_6_H_4_	0	90	99/1	83
10	**4i**, 2-FC_6_H_4_	rt	90	93/7	81
11	**4j**, 2-MeOC_6_H_4_	0	95	99/1	92
12	**4k**, 2-MeC_6_H_4_	0	91	97/3	94
13	**4l**, 3-ClC_6_H_4_	0	90	93/7	88
14^e^	**4m**, methyl	0	96	81/19	ND

^a^Reagents and conditions: 2.0 mol % Pd_2_(dba)_3_, 4.0 mol % **L1d**, 0.5 mmol allyl substrate, 1.5 mmol dimethyl malonate, 1.5 mmol BSA, 3.0 mol % NaOAc, DCE (5 mL). ^b^Isolated yield after 12 h. ^c^Determined by ^1^H NMR of the crude. ^d^Determined by HPLC. ^e^[Pd(C_3_H_5_)Cl]_2_ as the Pd precursor.

**Scheme 3:**
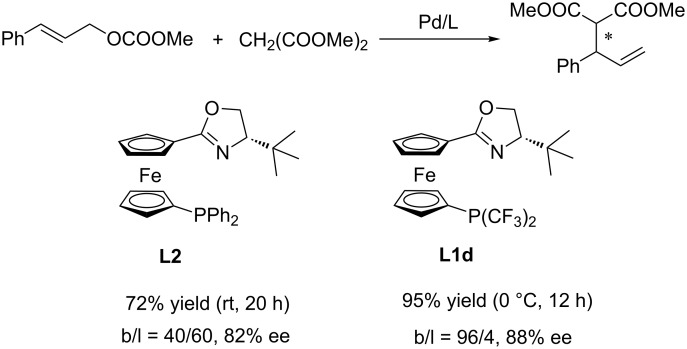
Comparison of the effect of ligands in the reaction.

**Figure 2:**
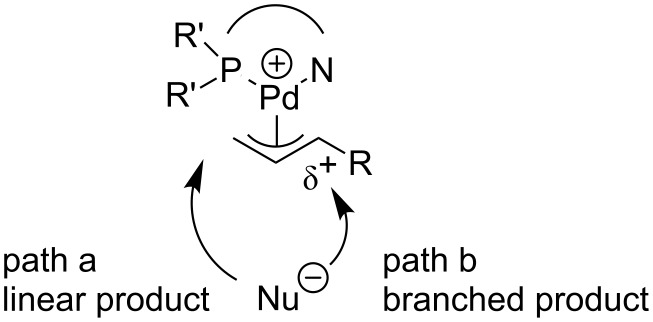
Preliminary explanation of the regioselectivity.

We conducted some control experiments to probe the effect of the bis(trifluoromethyl) group in the ligands (Scheme 3). With ferrocence-based biphenyl phosphine-oxazoline **L2** as the ligand, the Pd-catalyzed allylic alkylation of cinnamyl carbonate with dimethyl malonate afforded the linear product as the major product (b/l: 40/60). Whereas the corresponding ligand **L1d** with two CF_3_ groups (instead of two phenyl groups) at the P atom improved the regioselectivity significantly (b/l: 96/4). A preliminary explanation was described in Figure 2. In addition to the effect of different metals, there are at least two additional factors controlling the regioselectivity of the allylic alkylation reaction. The steric factor favors path a since the terminal allylic carbon is less hindered. In contrast, when the R group has the ability to stabilize the carbocation, the electronic factor would favor the formation of the branched product (path b). The phosphorus atom has a stronger *trans* effect comparing with the oxazoline nitrogen, indicating that the carbon *trans* to phosphorus atom bears more electropositivity [46]. This fact may be responsible for the preferred placement of the substituted allylic carbon in the *trans* position to the phosphorus atom to better stabilize the electropositivity of the carbon. When the nucleophile attacks the more electropositive substituted allylic carbon terminus, a branched product will be formed. The introduction of the CF_3_ group on the phosphorus atom further increases the *trans* influence of the P(CF_3_)_2_ moiety and enhances the electronic factor, providing a better branched-product selectivity. Further experimental studies and computational investigation are still needed to confirm this hypothesis.

## Conclusion

In summary, we have synthesized a class of novel and efficient bis(trifluoromethyl)phosphine-oxazolines as π-acceptor ligands which have shown good to excellent regio- and enantioselectivity for the Pd-catalyzed asymmetric allylic alkylation reaction of monosubstituted allyl carbonates. Further studies on the synthesis of 1-[bis(perfluoroalkyl)phosphine]-1’-oxazolinylferrocene ligands and their applications in asymmetric catalysis are ongoing in our lab.

## Supporting Information

File 1 Experimental, characterization data and spectra.
