# Supplementary material for: Synthesis of 1-[bis(trifluoromethyl)phosphine]-1’-oxazolinylferrocene ligands and their application in regio- and enantioselective Pd-catalyzed allylic alkylation of monosubstituted allyl substrates
Source: Beilstein J Org Chem. 2014 May 30;10:1261–6. doi: 10.3762/bjoc.10.126 (PMC4077528; doi:10.3762/bjoc.10.126)
Supplement: File 1 — Experimental, characterization data and spectra. [file Beilstein_J_Org_Chem-10-1261-s001.pdf]

Supporting Information

for

Synthesis of 1-[bis(trifluoromethyl)phosphine]-1'-oxazolinyl-ferrocene ligands and their application in regio- and enantioselective Pd-catalyzed allylic alkylation of monosubstituted allyl substrates

Zeng-Wei Lai[1,2], Rong-Fei Yang[3], Ke-Yin Ye[1], Hongbin Sun*[2] and Shu-Li You*[1]

Address: [1]State Key Laboratory of Organometallic Chemistry, Shanghai Institute of Organic Chemistry, Chinese Academy of Sciences, 345 Lingling Lu, Shanghai 200032, China, [2]State Key Laboratory of Natural Medicines and Center of Drug Discovery, China Pharmaceutical University, 24 Tongjia Xiang, Nanjing 210009, China and [3]Process Development and Manufacturing Department, Pharmaron (Beijing) Co. Ltd., 6 Taihe Road, BDA, Beijing, 100176, China.

Email: Shu-Li You* - slyou@sioc.ac.cn; Hongbin Sun* - hbsun2000@yahoo.com

* Corresponding author

Experimental, characterization data and spectra

Table of contents

General information S2

Experimental details and characterization data S3–8

References S9

Copies of NMR spectra S10–38

Copies of HPLC chromatograms S39–50

General information

Unless stated otherwise, all reactions were performed under a dry argon atmosphere with dry solvents under anhydrous conditions. Liquid reagents and solvents were transferred via syringe using standard Schlenk techniques. Dry diethyl ether (Et_2O) was distilled over sodium–potassium alloy. Dichloromethane (DCM), dimethylformamide (DMF), acetonitrile, and 1,2-dichloroethane (DCE) were distilled over calcium hydride. All reagents were obtained from commercial sources and used without further purification. ^{1}H and ^{13}C NMR spectra were recorded on a Varian instrument (300 MHz and 75 MHz, 400 MHz and 100 MHz, respectively) and internally referenced to tetramethylsilane signal or residual protio solvent signals. ^{19}F and ^{31}P NMR spectra were recorded on an Agilent instrument (376 and 162 MHz respectively). ^{19}F NMR chemical shifts were determined relative to $CFCl_3$ as internal standard and ^{31}P NMR spectra were referenced to an external 85% H_3PO_4 signal (0.0 ppm). Data for ^{1}H NMR are recorded as follows: chemical shift (δ, ppm), multiplicity (s = singlet, d = doublet, t = triplet, m = multiplet or unresolved, br = broad singlet, coupling constant (s) in Hz, integration). Data for ^{13}C NMR are reported in terms of chemical shift (δ, ppm).

Compounds **3** were prepared from ferrocene in three steps according to the reported procedures [1-3].

General procedure for the synthesis of ligands

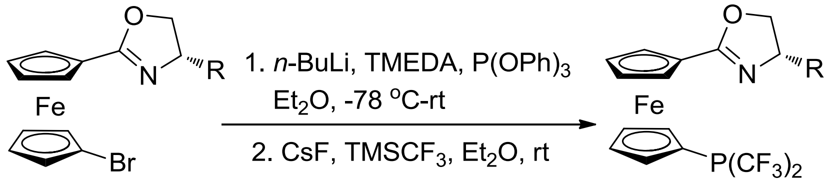

3 **L1**

To a suspension of the corresponding 1-oxazolinyl-1'-bromoferrocenes **3** (9.0 mmol) in anhydrous Et_2O (55 mL) was added TMEDA (1.6 mL, 10.8 mmol) and a solution of *n*-BuLi in hexane (4.5 mL, 2.4 M, 10.8 mmol) at −78 °C. After being stirred for 2 h at that temperature, the reaction mixture was added slowly to a solution of $P(OPh)_3$ (3 mL, 11.7 mmol) in Et_2O and then warmed to rt. The reaction was stirred at rt overnight until there was no change as monitored by ^{31}P NMR spectroscopy. The suspension was filtered through a pad of silica gel, washed with DCM and concentrated in vacuo. The crude product was used for the next step without purification. To a stirred solution of above crude product in Et_2O (55 mL) was added CsF (19.8 mmol, 3 g) and $TMSCF_3$ (27 mmol, 3.9 mL). The reaction was stirred at rt for 4 h, concentrated in vacuo, and purified by silica gel column chromatography (petroleum ether/DCM 10:1) to give the ligand.

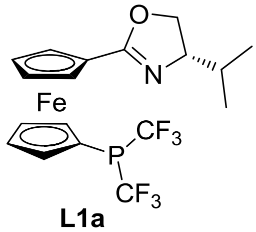

L1a

(*S*)-1-[4,5-Dihydro-4-isopropyloxazol-2-yl]-1'-[bis(trifluoromethyl)phosphino]-ferrocene (**L1a**)

Orange oil, 21% yield, IR (neat) Vmax cm^{-1}: 2962, 1259, 1087, 1016, 796; $[\alpha]_D^{20}$ = -29.9 (*c* 0.45, $CHCl_3$); ^1H NMR (400 MHz, $CDCl_3$) δ 4.85 (s, 1H), 4.81 (s, 1H), 4.55 (s, 2H), 4.45 (s, 2H), 4.41 (s, 2H), 4.28 (dd, *J* = 17.6, 9.2 Hz, 1H), 4.04 (dd, *J* = 16, 7.6 Hz, 1H), 4.00-3.90 (m, 1H), 1.82 (m, 1H), 0.98 (d, *J* = 6.8 Hz, 3H), 0.90 (d, *J* = 6.8 Hz, 3H); ^{13}C NMR (100 MHz, $CDCl_3$) δ 164.1, 75.0, 74.9, 74.8, 74.7, 74.7, 74.7, 72.6, 72.4, 71.6, 71.6, 70.5, 70.4, 69.6, 32.3, 18.8, 17.9. ^{31}P NMR (162 MHz, $CDCl_3$) δ -1.72 (sept, *J* = 73.0 Hz) ; ^{19}F NMR (376 MHz, $CDCl_3$) δ -53.85 (dq, *J* = 73.3, 7.3 Hz, 3F), -53.94 (dq, *J* = 73.4, 7.3 Hz, 3F). HRMS (MALDI-FT) m/z: calcd for $C_{18}H_{19}NOF_6P^{54}Fe$ $[M+1]^+$: 464.0493, found 464.0499

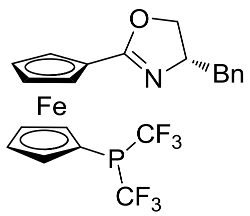

L1b

(*S*)-1-[4-benzyl-4,5-dihydrooxazol-2-yl]-1'-[bis(trifluoromethyl)phosphino]ferrocene (**L1b**)

Orange oil, 25% yield, IR (neat) νmax cm^{-1}: 2961, 2926, 1656, 1259, 1184, 1094, 1018, 797; $[\alpha]_D^{16}$ = -10.8 (*c* 0.55, $CHCl_3$); ^1H NMR (400 MHz, $CDCl_3$) δ 7.31-7.22 (m, 5H), 4.85 (s, 2H), 4.50-4.44 (m, 7H), 4.26 (t, *J* = 8.5 Hz, 1H), 4.07 (t, *J* = 7.5 Hz, 1H), 3.30-3.08 (m, 1H), 2.72 (m, 1H); ^{13}C NMR (75 MHz, $CDCl_3$) δ 164.9, 137.6, 129.3, 128.5, 126.5, 75.0, 74.8, 74.7, 72.11, 71.7, 71.4, 70.4, 67.7, 41.5; ^{31}P NMR (162 MHz, $CDCl_3$) δ -1.74 (sept, *J* = 73.0 Hz); ^{19}F NMR (376 MHz, $CDCl_3$) δ -53.80 (dq, *J* = 73.3, 6.4 Hz, 3F), -53.86 (dq, *J* = 73.3, 6.4 Hz, 3F). HRMS (MALDI-FT) m/z: calcd for $C_{22}H_{19}NOF_6P^{54}Fe$ $[M+1]^+$: 512.0510, found 512.0499

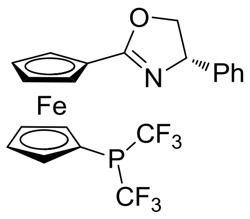

L1c

(*S*)-1-[4,5-dihydro-4-phenyloxazol-2-yl]-1'-[bis(trifluoromethyl)phosphino]ferrocene (**L1c**)

Orange oil, 18% yield, IR (neat) νmax cm^{-1}: 2961, 2926, 1653, 1259, 1182, 1096, 974, 954, 796; $[\alpha]_D^{23}$ = -64.3 (*c* 0.6, $CHCl_3$); ^1H NMR (400 MHz, $CDCl_3$) δ 7.37-7.34 (m, 2H), 7.30-7.28 (m, 3H), 5.24 (dd, *J* = 9.9, 8.1 Hz, 1H), 4.94 (dd, *J* = 7.9, 1.2 Hz, 2H), 4.71 (dd, *J* = 10.0, 8.4 Hz, 1H), 4.62 (s, 2H), 4.51 (s, 2H), 4.48 (s, 2H), 4.21 (t, *J* = 8.2 Hz, 1H); ^{13}C NMR (100 MHz, $CDCl_3$) δ 165.7, 142.1, 128.7, 127.6, 126.5, 75.0, 74.9, 74.8, 74.7 (m), 74.6, 71.9, 71.83, 71.81, 70.6, 69.9; ^{31}P NMR (162 MHz, $CDCl_3$) δ -1.92 (sept, *J* = 74.5 Hz); ^{19}F NMR (376 MHz, $CDCl_3$) δ -53.76 (dq, *J* = 73.3, 7.5 Hz, 3F), -53.86 (dq, *J* = -73.3, 7.3 Hz, 3F); HRMS (MALDI-FT) m/z: calcd for $C_{21}H_{17}NOF_6P^{54}Fe$ $[M+1]^+$: 498.0347, found 498.0343.

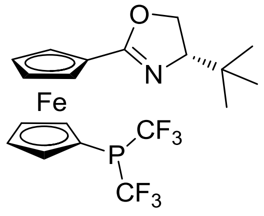

L1d

(*S*)-1-[4,5-Dihydro-4-*tert*-butyloxazol-2-yl]-1'-[bis(trifluoromethyl)phosphino]-ferrocene (**L1d**)

Orange solid, 35% yield, mp = 56 °C, IR (neat) νmax cm^{-1}: 2961, 1258, 1086, 1012, 792; $[\alpha]_D^{21}$ = -57.0 (*c* 0.25, $CHCl_3$). ^1H NMR (400 MHz, $CDCl_3$) δ 4.89 (s, 1H), 4.83 (s, 1H), 4.58 (s, 2H), 4.48 (s, 2H), 4.44 (s, 2H), 4.30-4.20 (m, 1H), 4.16 (t, *J* = 8.2 Hz, 1H), 3.91 (dd, *J* = 9.9, 8.0 Hz, 1H), 0.95 (s, 9H); ^{13}C NMR (100 MHz, $CDCl_3$) δ 164.00, 76.09, 74.84 (d, *J* = 6 Hz) , 74.61 (m), 72.72, 71.52, 71.45, 70.39, 68.41,

33.45, 25.82; ^{31}P NMR (162 MHz, $CDCl_3$) δ -2.35 (sept, *J* = 73.3 Hz); ^{19}F NMR (376 MHz, $CDCl_3$) δ -53.86 (d, *J* = 73.2 Hz, 6F). HRMS (MALDI-FT) m/z: calcd for $C_{19}H_{21}NOF_6P^{54}Fe$ $[M+1]^+$: 478.0649, found 478.0655

General procedure for the Pd-catalyzed allylic alkylation reaction.

$Pd_2(dba)_3$ (9.2 mg, 0.01 mmol) and ligand **L1d** (9.6 mg, 0.02 mmol) were dissolved in dry $(CH_2Cl)_2$ (5.0 mL) and then the reaction mixture was stirred for 30 min at rt under an atmosphere of argon. To this stirred solution was successively added allyl carbonate (0.5 mmol), dimethylmalonate (0.17 mL, 1.5 mmol), *N,O*-bis(trimethylsilyl)acetamide (BSA) (0.37 mL, 1.5 mmol), and NaOAc (1.0 mg, 0.015 mmol). The reaction was stirred at 0 °C and monitored by TLC. After completion, the reaction mixture was diluted with DCM (25 mL) and washed twice with ice-cold saturated aqueous ammonium chloride. The organic phase was dried over anhydrous Na_2SO_4, filtered and concentrated under reduced pressure. The regioselectivity of the reaction was determined by ^{1}H NMR spectroscopy of the crude product. The residue was purified with silica gel column chromatography (petroleum ether/ethyl acetate 30:1) to provide the product. The enantiomeric purities were determined by HPLC.

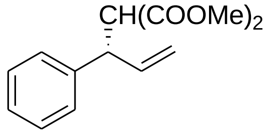

Dimethyl 3-phenyl-1-butene-4,4-dicarboxylate (6a)[4]

Colorless oil, 91% yield, 88% ee [Daicel CHIRALPAK OJ-H (0.46 cm x 25 cm). hexane/2-propanol = 95/5; flow rate = 1.0 mL/min; detection wavelength = 220 nm; t_R = 19.53 (major), 21.99 (minor) min] $[\alpha]_D^{24}$ = - 32.4 (*c* 1.0, $CHCl_3$). ^{1}H NMR (300 MHz, $CDCl_3$) δ 7.29-7.20 (m, 5H), 5.99 (ddd, *J* = 17.4, 9.0, 8.4 Hz, 1H), 5.12 (d, *J* = 16.2 Hz, 1H), 5.08 (d, *J* = 8.2 Hz, 1H), 4.11 (dd, *J* = 10.4 Hz, *J* = 8.7 Hz, 1H), 3.86 (d, *J* = 11.1 Hz, 1H), 3.74 (s, 3H), 3.49 (s, 3H).

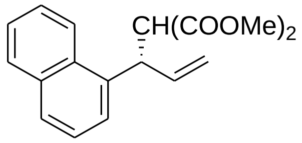

Dimethyl 3-(1-naphthyl)-1-butene-4,4-dicarboxylate (6b)[5]

Colorless oil, 95% yield, 92% ee [Daicel CHIRALPAK OJ-H (0.46 cm x 25 cm). hexane/2-propanol = 90/10; flow rate = 1.0 mL/min; detection wavelength = 254 nm; t_R = 12.5 (major), 17.4 (minor) min] $[\alpha]_D^{23}$ = -35.9 (*c* 0.85, $CHCl_3$). ^{1}H NMR (400 MHz, $CDCl_3$) δ 8.26 (d, *J* = 8.4 Hz, 1H), 7.85 (d, *J* = 8.0 Hz, 1H), 7.75 (d, *J* = 7.9 Hz, 1H), 7.56 (t, *J* = 7.1 Hz, 1H), 7.49 (t, *J* = 7.4 Hz, 1H), 7.47-7.37 (m, 2H), 6.09 (ddd, *J* =17.3, 9.7, 8.0 Hz, 1H), 5.18 (d, *J* = 17.1 Hz, 1H), 5.11 (d, *J* = 10.2 Hz, 1H), 5.04 (dd, *J* = 10.0, 9.2 Hz, 1H), 4.17 (d, *J* = 10.8 Hz, 1H), 3.79 (s, 3H), 3.39 (s, 3H).

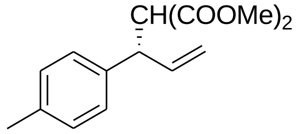

Dimethyl 3-(4-methylphenyl)-1-butene-4,4-dicarboxylate (6c)[6]

Colorless oil, 93% yield, 85% ee [Daicel CHIRALCEL OD-H (0.46 cm x 25 cm). hexane/2-propanol = 69/1; flow rate = 0.7 mL/min; detection wavelength = 220 nm; t_R = 9.87 (minor), 10.6 (major) min] $[\alpha]_D^{23}$ = -25.5 (*c* 1.2, $CHCl_3$). ^1H NMR (400 MHz, $CDCl_3$) δ 7.11 (s, 4H), 5.98 (ddd, *J* = 17.1, 10.2, 8.2 Hz, 1H), 5.11 (d, *J* = 17.0 Hz, 1H), 5.07 (d, *J* = 10.5 Hz, 1H), 4.08 (dd, *J* = 11.0, 8.3 Hz, 1H), 3.86 (d, *J* = 11.0 Hz, 1H), 3.74 (s, 3H), 3.51 (s, 3H), 2.31 (s, 3H).

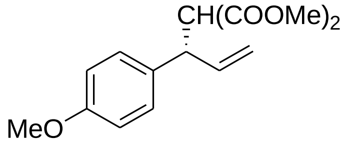

Dimethyl 3-(4-methoxyphenyl)-1-butene-4,4-dicarboxylate (6d)[7]

Colorless oil, 96% yield, 82% ee [Daicel CHIRALCEL OD-H (0.46 cm x 25 cm). hexane/2-propanol = 90/10; flow rate = 0.5 mL/min; detection wavelength = 220 nm; t_R = 11.43 (minor), 12.76 (major) min] $[\alpha]_D^{20}$ = -20.3 (*c* 0.75, $CHCl_3$). ^1H NMR (400 MHz, $CDCl_3$) δ 7.14 (d, *J* = 8.7 Hz, 2H), 6.83 (d, *J* = 8.7 Hz, 2H), 5.97 (ddd, *J* = 17.1, 10.2, 8.1 Hz, 1H), 5.09 (d, *J* = 11.8 Hz, 1H), 5.06 (d, *J* = 8.7 Hz, 1H), 4.06 (dd, *J* = 10.9, 8.2 Hz, 1H), 3.82 (d, *J* = 11.1 Hz, 1H), 3.77 (s, 3H), 3.73 (s, 3H), 3.50 (s, 3H).

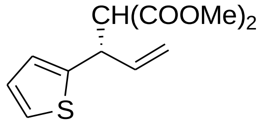

Dimethyl 3-(2-thienyl)-1-butene-4,4-dicarboxylate (6e)[5]

Colorless oil, 94% yield, 70% ee [Daicel CHIRALPAK OJ-H (0.46 cm x 25 cm). hexane/2-propanol = 98/2; flow rate = 1.0 mL/min; detection wavelength = 220 nm; t_R = 23.15 (major), 25.5 (minor) min] $[\alpha]_D^{23}$ = -30.4 (*c* 0.5, $CHCl_3$). ^1H NMR (300 MHz, $CDCl_3$) δ 7.18 (d, *J* = 5.0 Hz, 1H), 6.92 (t, *J* = 4.2 Hz, 1H), 6.88 (d, *J* = 3.0 Hz, 1H), 6.02 (ddd, *J* = 17.4, 9.8, 8.0 Hz, 1H), 5.19 (d, *J* = 18.0 Hz, 1H), 5.13 (d, *J* = 9.6 Hz, 1H), 4.42 (dd, *J* = 9.3, 9.09 Hz, 1H), 3.84 (d, *J* = 10.2 Hz, 1H), 3.73 (s, 3H), 3.61 (s, 3H).

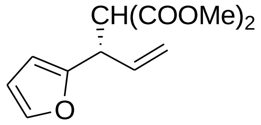

Dimethyl 3-(2-furanyl)-1-butene-4,4-dicarboxylate (6f)[8]

Colorless oil, 90% yield, 65% ee [Daicel CHIRALPAK OJ-H (0.46 cm x 25 cm). hexane/2-propanol = 95/5; flow rate = 0.6 mL/min; detection wavelength = 220 nm; t_R = 22.73 (major), 24.36 (minor) min] $[\alpha]_D^{23}$ = -9.4 (*c* 0.3, $CHCl_3$). ^1H NMR (400 MHz, $CDCl_3$) δ 7.28 (d, *J* = 1.8 Hz, 1H), 6.23 (dd, *J* = 3.2, 1.9 Hz, 1H), 6.06 (d, *J* = 3.3 Hz, 1H), 5.92 (ddd, *J* = 17.1, 10.2, 8.5 Hz, 1H), 5.14 (d, *J* = 17.1 Hz , 1H), 5.11 (d, *J* = 9.6 Hz , 1H), 4.17 (t, *J* = 9.3 Hz, 1H), 3.83 (d, *J* = 10.0 Hz, 1H), 3.70 (s, 1H), 3.68 (s, 3H), 3.60 (s, 3H).

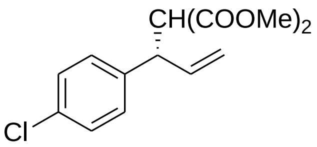

Dimethyl 3-(4-chlorophenyl)-1-butene-4,4-dicarboxylate (6g)[6]
Colorless oil, 91% yield, 83% ee [Daicel CHIRALCEL OD-H (0.46 cm x 25 cm). hexane/2-propanol = 69/1; flow rate = 0.7 mL/min; detection wavelength = 220 nm; t_R = 7.15 (minor), 7.75 (major) min] $[\alpha]_D^{23}$ = -27.6 (*c* 1.0, $CHCl_3$). ^1H NMR (400 MHz, $CDCl_3$) δ 7.28 (d, *J* = 8.2 Hz, 2H), 7.17 (d, *J* = 8.6 Hz, 2H), 5.95 (ddd, *J* = 18.3, 9.8, 8.0 Hz, 1H), 5.11 (d, *J* = 16.8 Hz, 1H), 5.10 (d, *J* = 10.0 Hz, 1H), 4.10 (dd, *J* = 10.8, 8.2 Hz, 1H), 3.82 (d, *J* = 11.0 Hz, 1H), 3.74 (s, 3H), 3.52 (s, 3H).

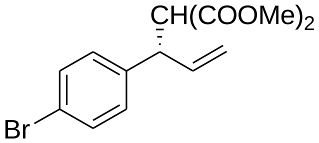

Dimethyl 3-(4-bromophenyl)-1-butene-4,4-dicarboxylate (6h)[8]
Colorless oil, 90% yield, 83% ee [Daicel CHIRALCEL OD-H (0.46 cm x 25 cm). hexane/2-propanol = 69/1; flow rate = 0.7 mL/min; detection wavelength = 220 nm; t_R = 11.53 (minor), 12.27 (major) min] $[\alpha]_D^{23}$ = -29.2 (*c* 1.1, $CHCl_3$). ^1H NMR (400 MHz, $CDCl_3$) δ 7.42 (d, *J* = 8.4 Hz, 2H), 7.10 (d, *J* = 8.4 Hz, 2H), 5.94 (ddd, *J* = 17.0, 10.4, 8.1 Hz, 1H), 5.08 (d, *J* = 16.8 Hz, 1H), 5.07 (d, *J* = 10.4 Hz, 1H),4.07 (dd, *J* = 10.9, 8.2 Hz, 1H), 3.82 (d, *J* = 11.0 Hz, 1H), 3.74 (s, 3H), 3.52 (s, 3H).

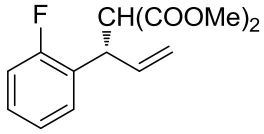

Dimethyl 3-(2-fluorophenyl)-1-butene-4,4-dicarboxylate (6i)[9]
Colorless oil, 90% yield, 81% ee [Daicel CHIRALCEL OD-H (0.46 cm x 25 cm). hexane/2-propanol = 95/5; flow rate = 0.6 mL/min; detection wavelength = 220 nm; t_R = 9.03 (minor), 9.77 (major) min] $[\alpha]_D^{23}$ = -33.4 (*c* 1.2, $CHCl_3$). ^1H NMR (400 MHz, $CDCl_3$) δ 7.20-7.12 (m, 2H), 7.02 (t, *J* = 7.5 Hz, 1H), 6.99-6.93 (m, 1H), 5.98 (ddd, *J* = 17.1, 9.9, 8.6 Hz, 1H), 5.10 (d, *J* = 17.0 Hz, 1H), 5.05 (d, *J* = 10.2 Hz, 1H), 4.33-4.22 (m, 1H), 3.97 (d, *J* = 11.1 Hz, 1H), 3.69 (s, 1H), 3.46 (s, 3H).

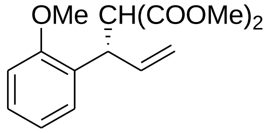

Dimethyl 3-(2-methoxyphenyl)-1-butene-4,4-dicarboxylate (6j)[9]
Colorless oil, 95% yield, 92% ee [Daicel CHIRALCEL OD-H (0.46 cm x 25 cm). hexane/2-propanol = 95/5; flow rate = 0.6 mL/min; detection wavelength = 220 nm; t_R = 10.32 (minor), 11.9 (major) min] $[\alpha]_D^{23}$ = -35.1 (*c* 1.0, $CHCl_3$). ^1H NMR (400 MHz, $CDCl_3$) δ 7.18-7.08 (m, 2H), 6.82 (m, 2H), 6.16-5.99 (m, 1H), 5.07 (d, *J* = 17.1 Hz, 1H), 4.99 (d, *J* = 10.1 Hz, 1H), 4.34-4.24 (m, 1H), 4.14 (d, *J* = 10.7 Hz, 1H), 3.80 (s, 3H), 3.67 (s, 3H), 3.44 (s, 3H).

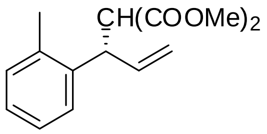

Dimethyl 3-(2-methylphenyl)-1-butene-4,4-dicarboxylate (6k)[10]
Colorless oil, 91% yield, 94% ee [Daicel CHIRALCEL OD-H (0.46 cm x 25 cm). hexane/2-propanol = 99/1; flow rate = 1.0 mL/min; detection wavelength = 220 nm; t_R = 7.7 (minor), 8.7 (major) min] $[\alpha]_D^{23}$ = -67.9 (*c* 1.0, $CHCl_3$). ^1H NMR (400 MHz, $CDCl_3$) δ 7.16-7.07 (m, 4H), 5.83 (ddd, *J* =8.0, 9.2, 18.0 Hz, 1H), 5.04 (d, *J* = 16.8 Hz, 1H), 5.02 (d, *J* = 10.4 Hz, 1H), 4.37 (dd, *J* = 11.4, 8.1 Hz, 1H), 3.95 (d, *J* = 11.4 Hz, 1H), 3.74 (s, 3H), 3.46 (s, 3H), 2.40 (s, 3H).

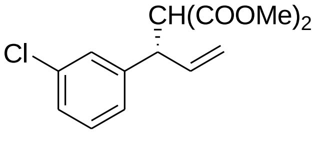

Dimethyl3-(3-chlorophenyl)-1-butene-4,4-dicarboxylate (6l)[8]
Colorless oil, 90% yield, 88% ee [Phenomenex CHIRALCEL PA-2 (0.46 cm x 25 cm). hexane/2-propanol = 95/5; flow rate = 1.0 mL/min; detection wavelength = 220 nm; t_R = 23.6 (major), 36.3 (minor) min] $[\alpha]_D^{23}$ = -26.1 (*c* 1.2, $CHCl_3$). ^1H NMR (400 MHz, $CDCl_3$) δ 7.19-7.12 (m, 3H), 7.06 (d, *J* = 7.0 Hz, 1H), 5.97-5.81 (m, 1H), 5.08 (d, *J* = 13.6 Hz, 1H), 5.06 (d, *J* = 10.4 Hz, 1H), 4.03 (dd, *J* = 10.8, 8.3 Hz, 1H), 3.78 (d, *J* = 11.0 Hz, 1H), 3.69 (s, 3H), 3.48 (s, 3H).

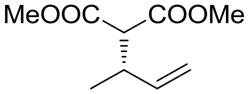

Dimethyl 3-methyl-1-butene-4,4-dicarboxylate (6m)[11]
Colorless oil, 96% yield, $[\alpha]_D^{24}$ = -2.25 (*c* 1.0, $CHCl_3$); ^1H NMR (400 MHz, $CDCl_3$) δ 5.67 (ddd, *J* = 17.3, 10.2, 8.1 Hz, 1H), 5.00 (d, *J* = 17.1 Hz, 1H), 4.92 (d, *J* = 10.3 Hz, 1H), 3.64 (s, 3H), 3.61 (s, 3H), 3.23 (d, *J* = 9.0 Hz, 1H), 2.88-2.82 (m, 1H), 1.00 (d, *J* = 6.8 Hz, 3H).

References

1. Dong, T.-Y.; Lai, L.-L. *J. Organomet. Chem*. **1996**, *509*, 131.
2. Chesney, A.; Bryce, M. R.; Chubb, R. W. J.; Batsanov, A.; Howard, J. *Synthesis* **1998**, 413.
3. Park, J.; Quan, Z.; Lee, S.; Ahn, K. H.; Cho, C.-W. *J. Organomet. Chem*. **1999**, *584*, 140.
4. Lloyd-Jones, G. C.; Pfaltz, A. *Angew. Chem., Int. Ed.* **1995**, *34*, 462.
5. Trost, B. M.; Hachiya, I. *J. Am. Chem. Soc.* **1998**, *120*, 1104.
6. Hayashi, T.; Okada, A.; Suzuka, T.; Kawatsura, M. *Org. Lett.* **2003**, *5*, 1713.
7. Kinoshita, N.; Marx, K. H.; Tanaka, K.; Tsubaki, K.; Kawabata, T.; Yoshikai, N.; Nakamura, E.; Fuji, K. *J. Org. Chem*. **2004**, *69*, 7960.
8. Hu, Z.; Li, Y.; Liu, K.; Shen, Q. *J. Org. Chem*. **2012**, 77, 7957.
9. Nemoto,T.; Sakamoto,T.; Fukuyama,T.; Hamada,Y. *Tetrahedron Lett.* **2007**, *48*, 4977.
10. Liu, W.-B.; Zheng, C.; Zhuo, C.-X.; Dai, L.-X.; You, S.-L. *J. Am. Chem. Soc.* **2012**, *134*, 4812.
11. Evans, P. A.; Nelson, J. D. *J. Am. Chem. Soc*. **1998**, *120*, 5581

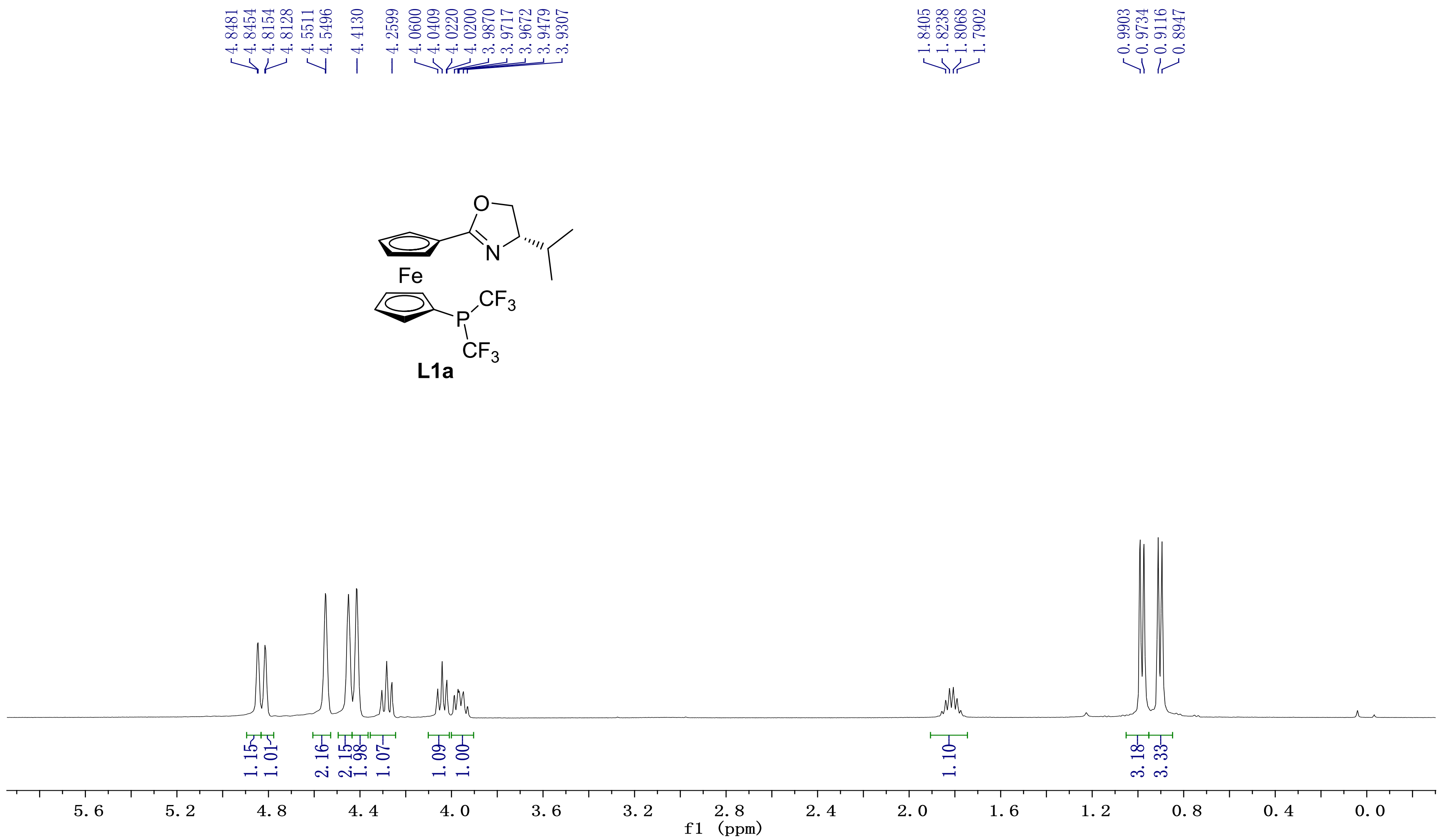

4.8481 4.8454 4.8154 4.8128 4.5511 4.5496 4.4130 4.2599 4.0600 4.0409 4.0220 4.0200 3.9870 3.9717 3.9672 3.9479 3.9307
1.8405 1.8238 1.8068 1.7902
0.9903 0.9734 0.9116 0.8947
O
N
Fe
P
CF3
CF3
L1a
1.15 1.01 2.16 2.15 1.98 1.07 1.09 1.00 1.10 3.18 3.33
5.6 5.2 4.8 4.4 4.0 3.6 3.2 2.8 2.4 2.0 1.6 1.2 0.8 0.4 0.0
f1 (ppm)

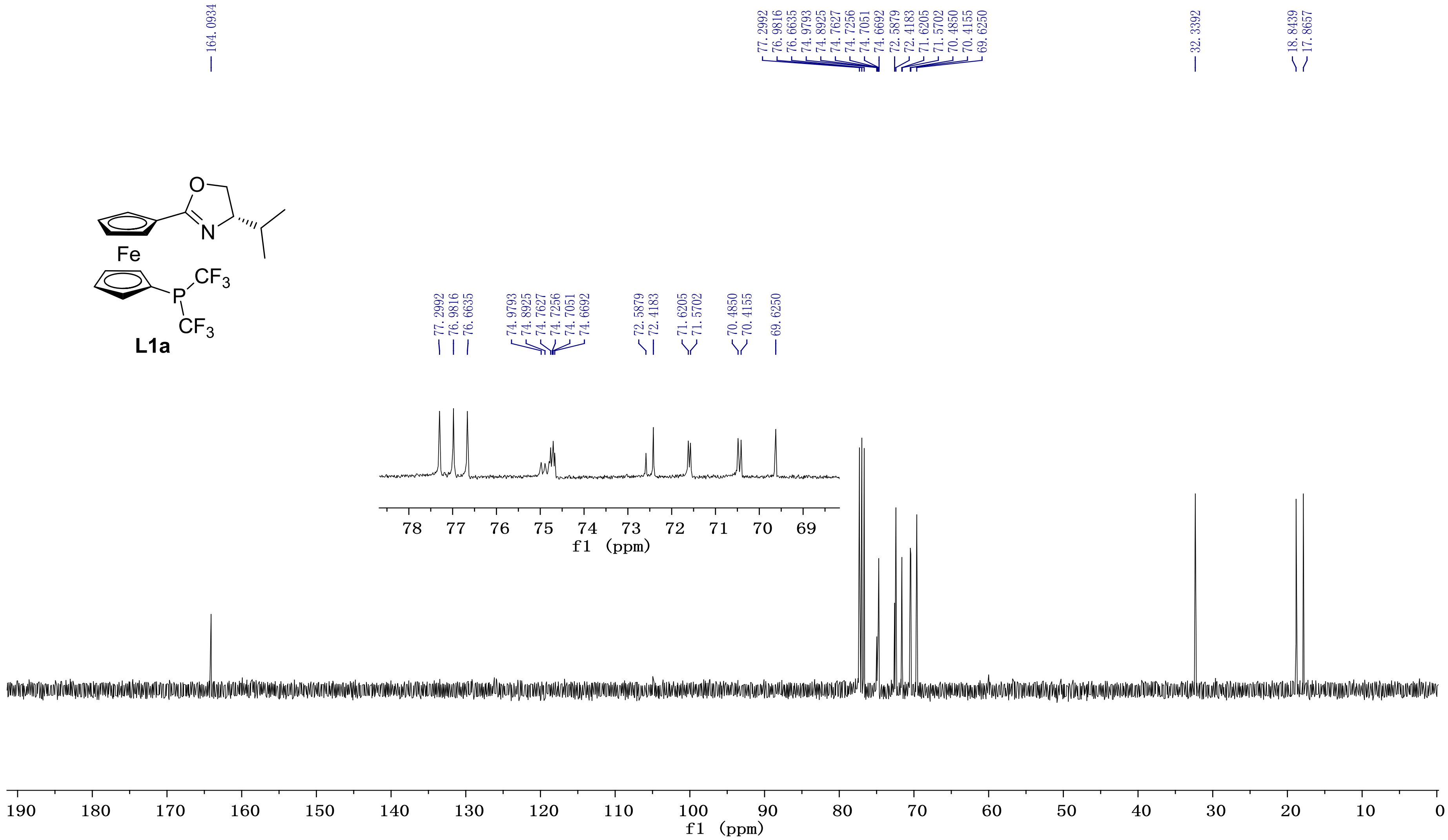

164.0934
77.2992
76.9816
76.6635
74.9793
74.8925
74.7627
74.7256
74.7051
74.6692
72.5879
72.4183
71.6205
71.5702
70.4850
70.4155
69.6250
32.3392
18.8439
17.8657
O
N
Fe
P
CF3
CF3
L1a
f1 (ppm)

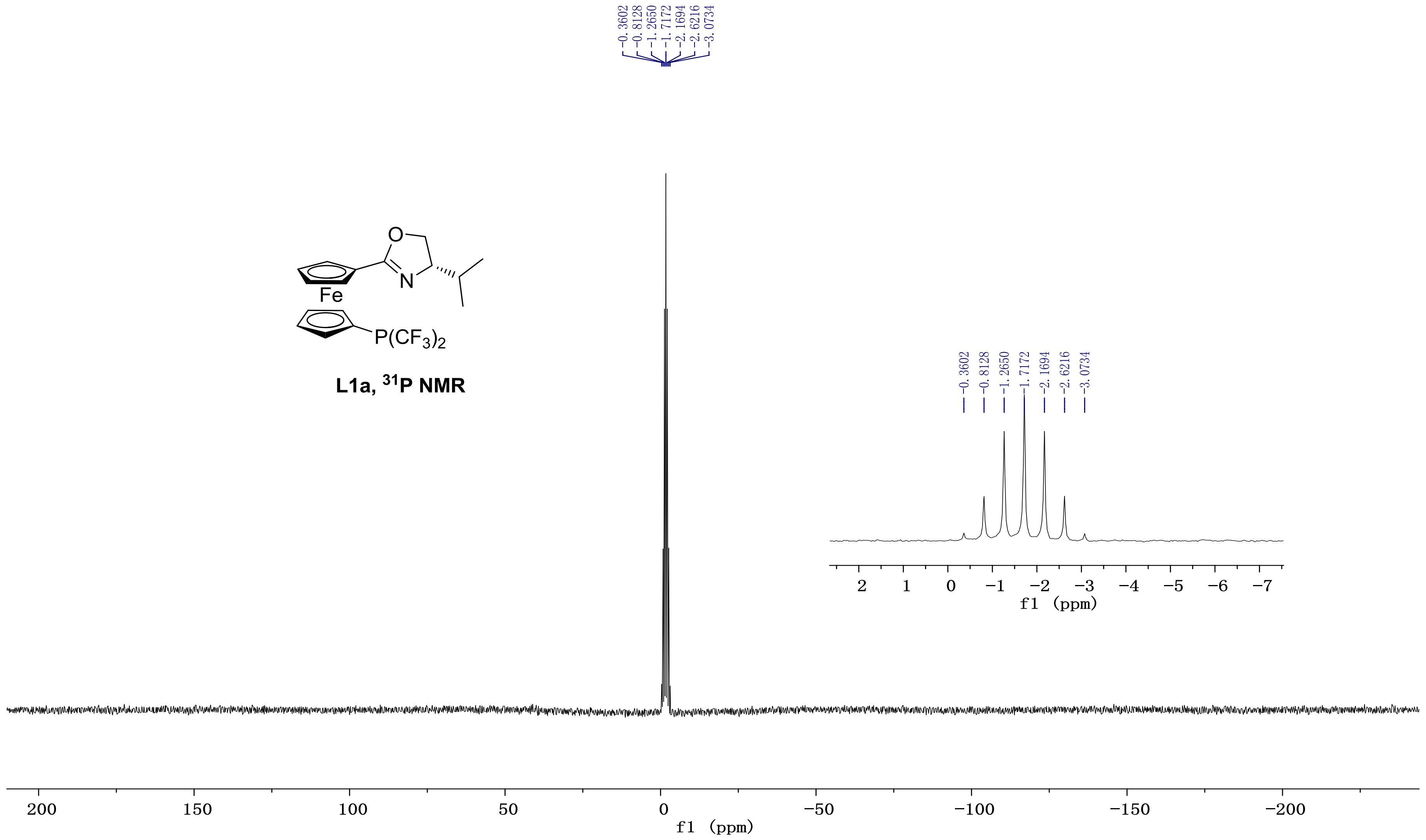

Fe
P(CF3)2
O
N
L1a, 31P NMR
−0.3602
−0.8128
−1.2650
−1.7172
−2.1694
−2.6216
−3.0734
f1 (ppm)

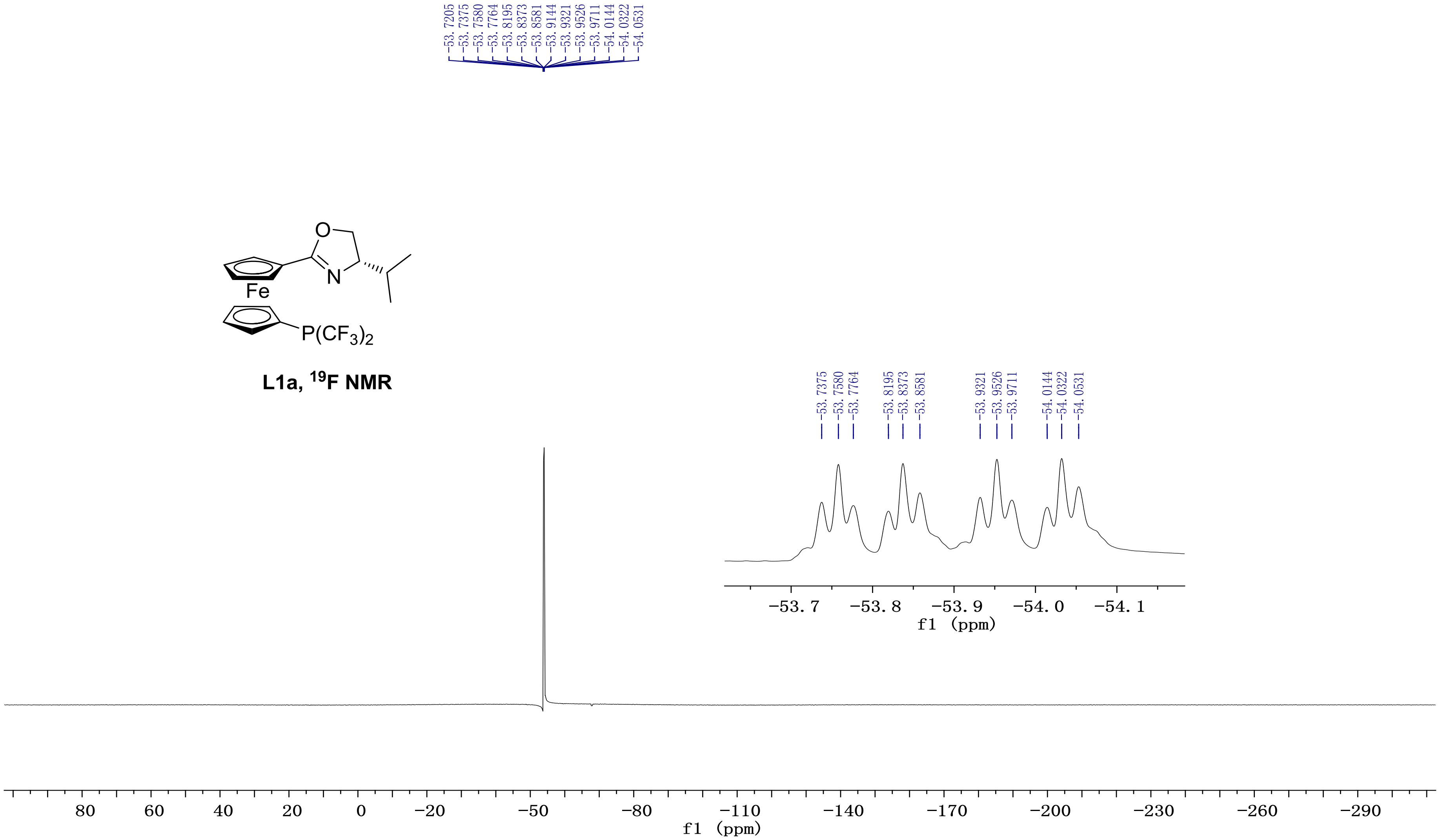
-53.7205
-53.7375
-53.7580
-53.7764
-53.8195
-53.8373
-53.8581
-53.9144
-53.9321
-53.9526
-53.9711
-54.0144
-54.0322
-54.0531
O
N
Fe
P(CF3)2
-53.7375
-53.7580
-53.7764
-53.8195
-53.8373
-53.8581
-53.9321
-53.9526
-53.9711
-54.0144
-54.0322
-54.0531
-53.7 -53.8 -53.9 -54.0 -54.1
f1 (ppm)
80 60 40 20 0 −20 −50 −80 −110 −140 −170 −200 −230 −260 −290
f1 (ppm)

L1a, ^{19}F NMR

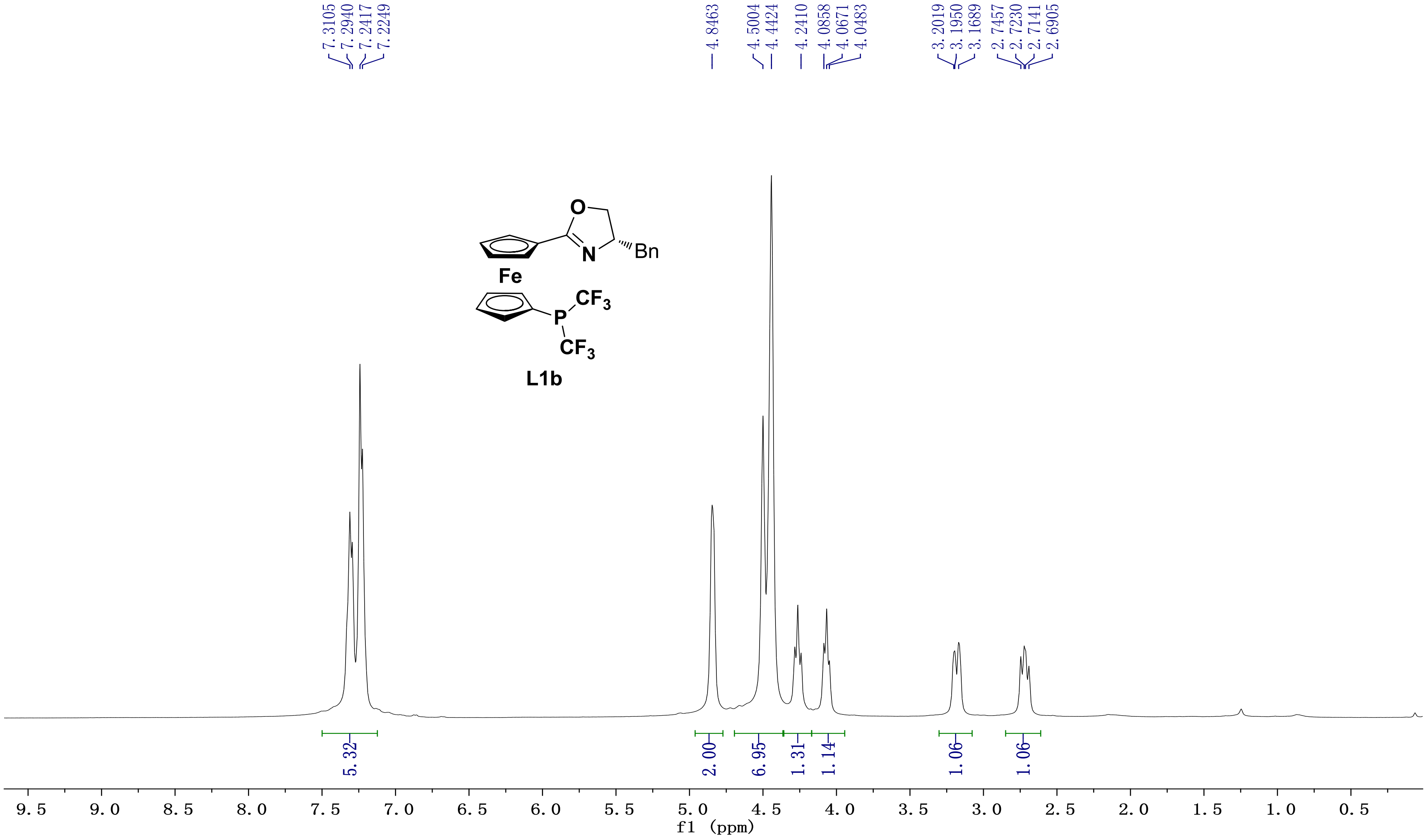
7.3105
7.2940
7.2417
7.2249
4.8463
4.5004
4.4424
4.2410
4.0858
4.0671
4.0483
3.2019
3.1950
3.1689
2.7457
2.7230
2.7141
2.6905
O
N
Bn
Fe
P
CF3
CF3
L1b
5.32
2.00
6.95
1.31
1.14
1.06
1.06
9.5
9.0
8.5
8.0
7.5
7.0
6.5
6.0
5.5
5.0
4.5
4.0
3.5
3.0
2.5
2.0
1.5
1.0
0.5
f1 (ppm)

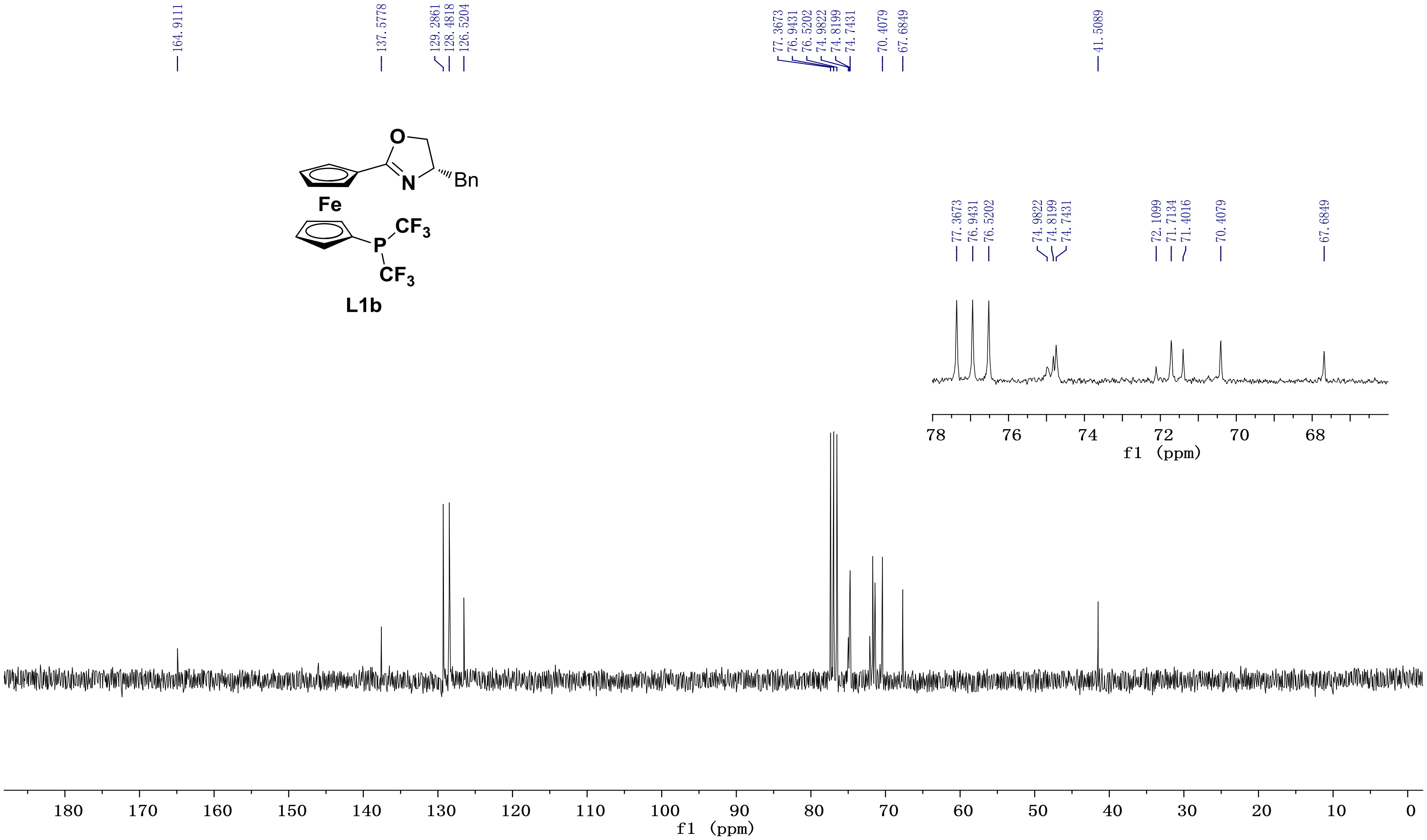
164.9111
137.5778
129.2861
128.4818
126.5204
77.3673
76.9431
76.5202
74.9822
74.8199
74.7431
70.4079
67.6849
41.5089
O
N
Bn
Fe
P
CF3
CF3
L1b
77.3673
76.9431
76.5202
74.9822
74.8199
74.7431
72.1099
71.7134
71.4016
70.4079
67.6849
78 76 74 72 70 68
f1 (ppm)
180 170 160 150 140 130 120 110 100 90 80 70 60 50 40 30 20 10 0
f1 (ppm)

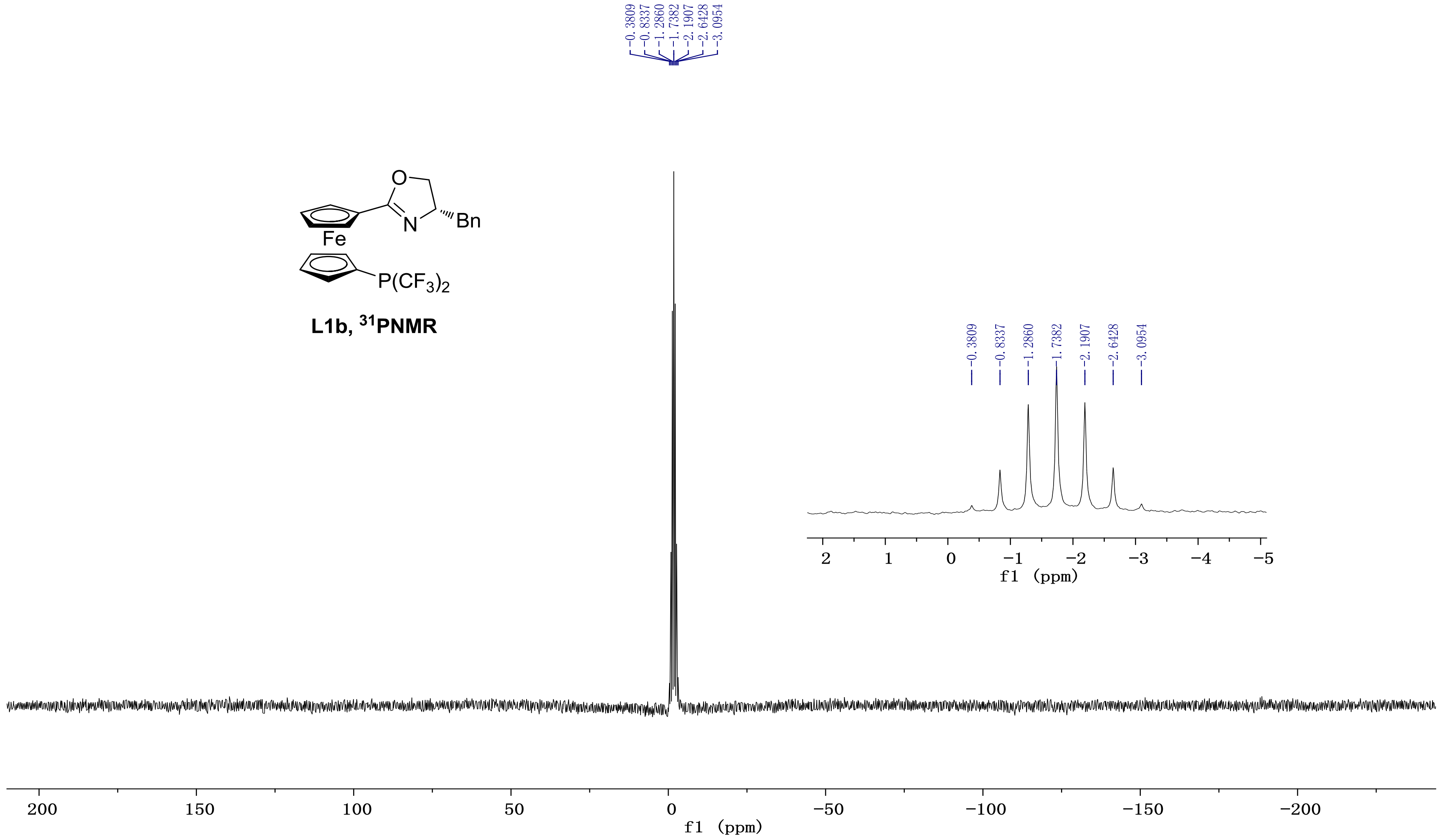
-0.3809
-0.8337
-1.2860
-1.7382
-2.1907
-2.6428
-3.0954
O
N
Bn
Fe
$P(CF_3)_2$
L1b, ^{31}PNMR
2 1 0 −1 −2 −3 −4 −5
f1 (ppm)
200 150 100 50 0 −50 −100 −150 −200
f1 (ppm)

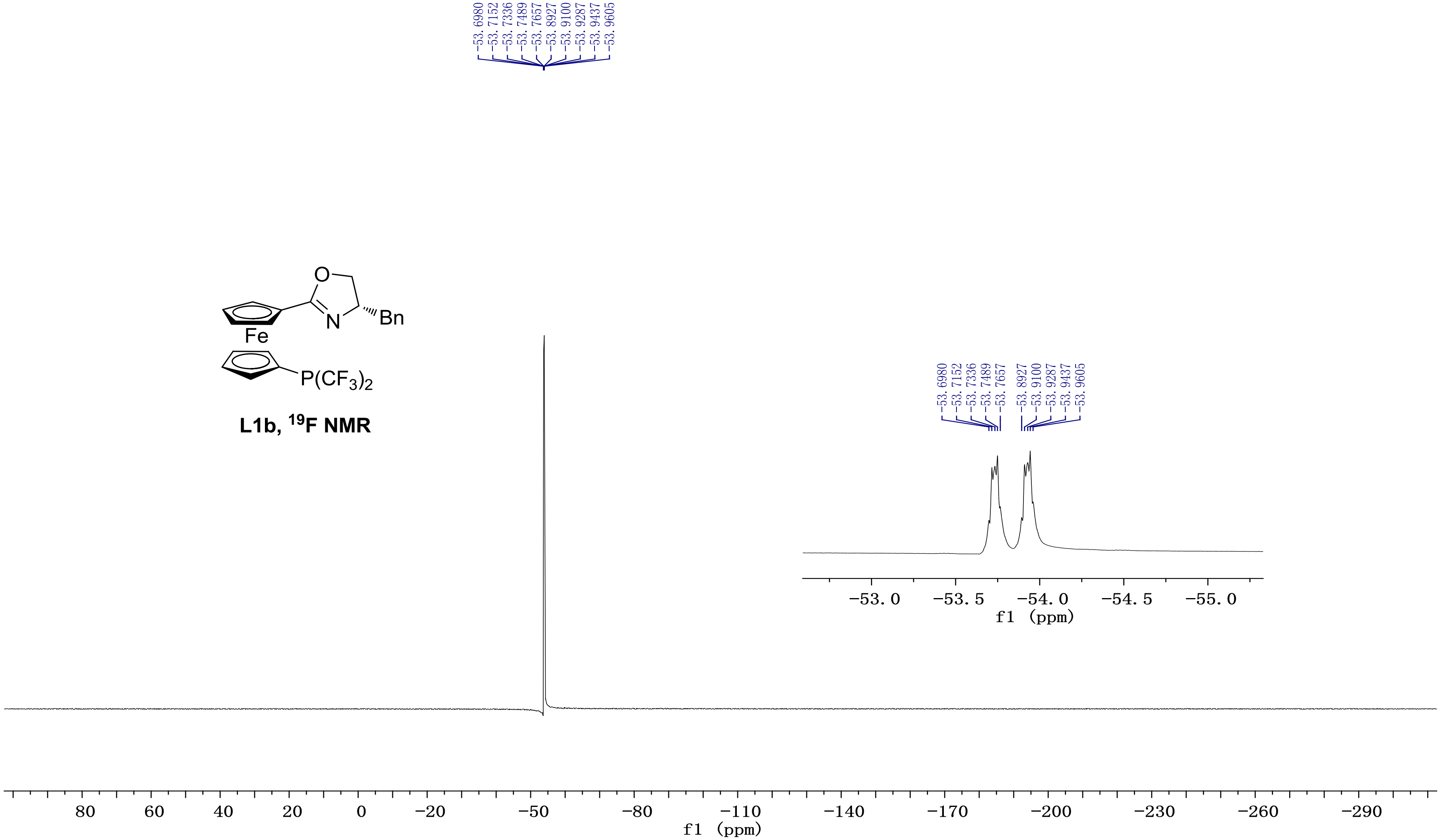
-53.6980
-53.7152
-53.7336
-53.7489
-53.7657
-53.8927
-53.9100
-53.9287
-53.9437
-53.9605
-53.0 -53.5 -54.0 -54.5 -55.0
f1 (ppm)
80 60 40 20 0 −20 −50 −80 −110 −140 −170 −200 −230 −260 −290
f1 (ppm)

L1b, ^{19}F NMR

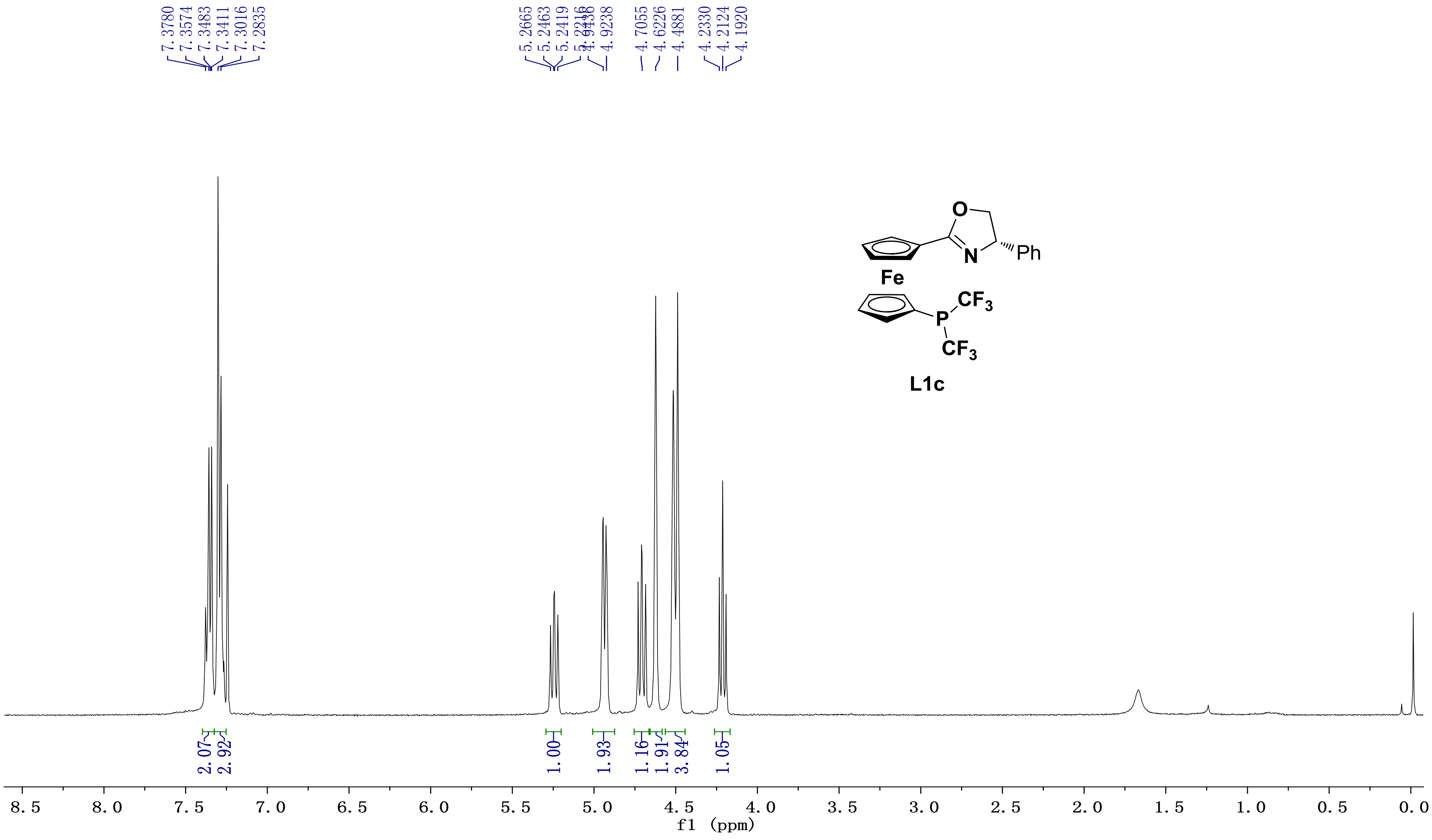
7.3780
7.3574
7.3483
7.3411
7.3016
7.2835
5.2665
5.2463
5.2419
4.9238
4.7055
4.6226
4.4881
4.2330
4.2124
4.1920
2.07
2.92
1.00
1.93
1.16
1.91
3.84
1.05
8.5
8.0
7.5
7.0
6.5
6.0
5.5
5.0
4.5
4.0
3.5
3.0
2.5
2.0
1.5
1.0
0.5
0.0
f1 (ppm)
O
N
Ph
Fe
P
CF3
CF3

L1c

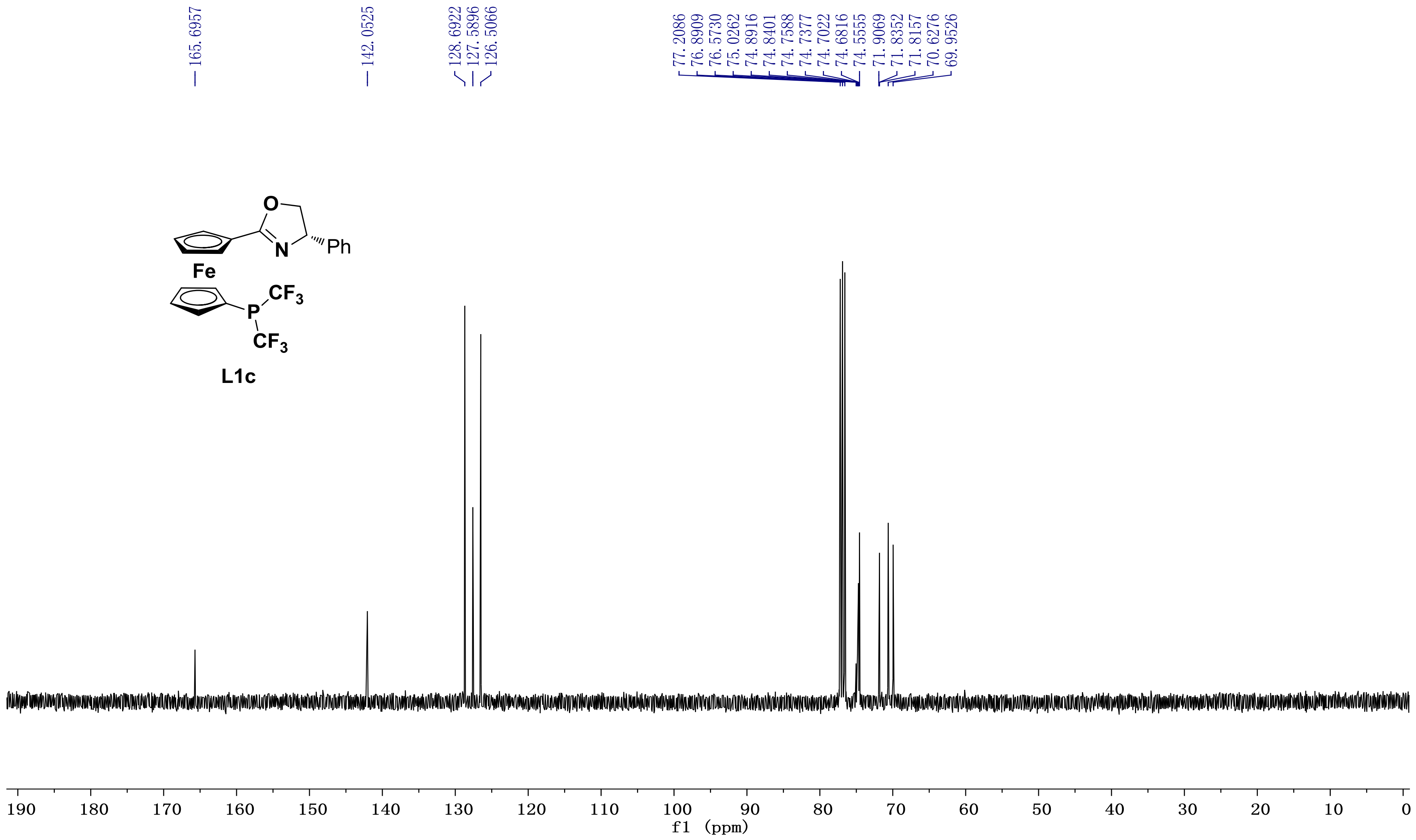
165.6957
142.0525
128.6922
127.5896
126.5066
77.2086
76.8909
76.5730
75.0262
74.8916
74.8401
74.7588
74.7377
74.7022
74.6816
74.5555
71.9069
71.8352
71.8157
70.6276
69.9526
O
N
Ph
Fe
P
CF3
CF3
L1c
190 180 170 160 150 140 130 120 110 100 90 80 70 60 50 40 30 20 10 0
f1 (ppm)

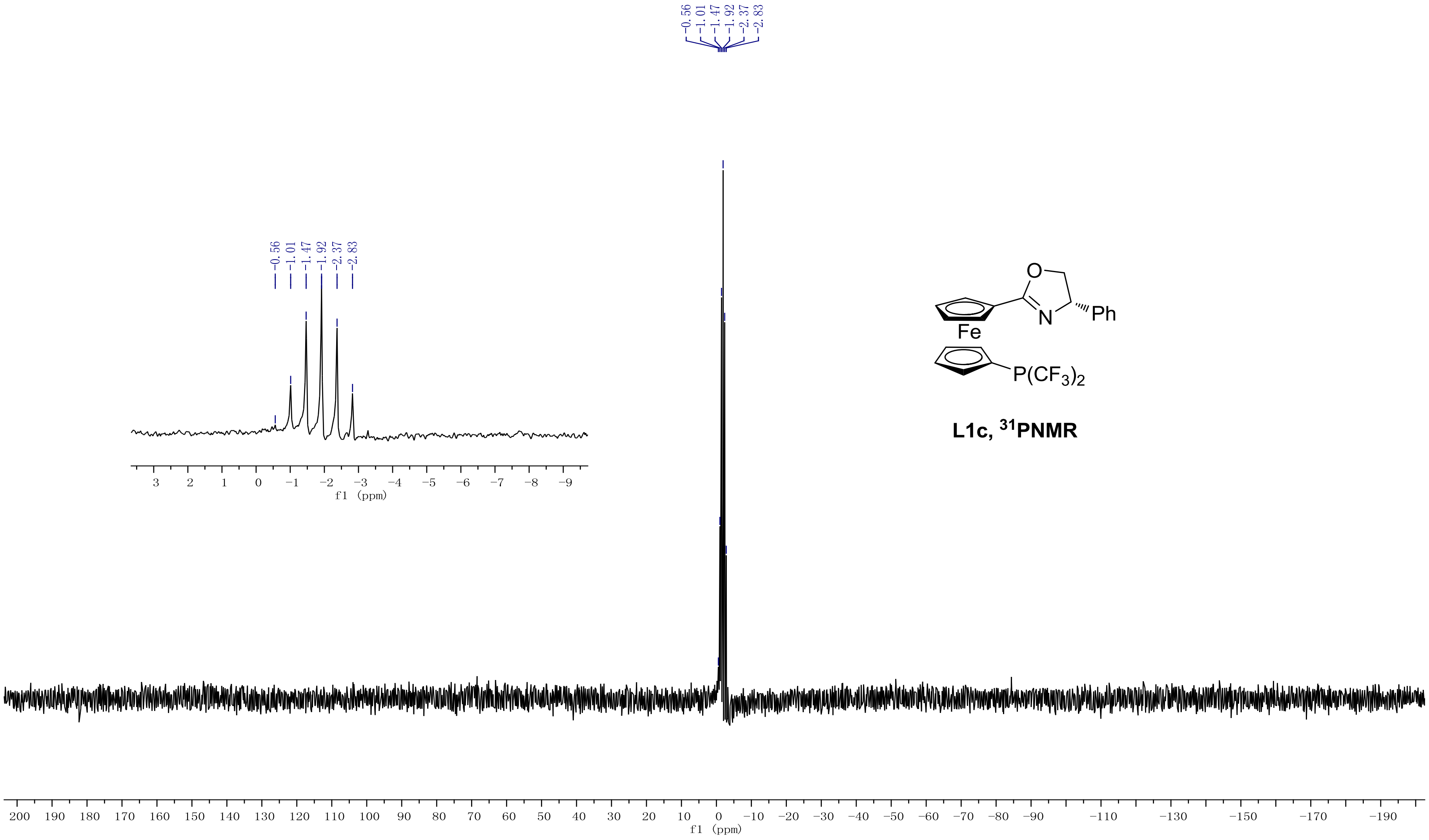
-0.56
-1.01
-1.47
-1.92
-2.37
-2.83
f1 (ppm)
O
N
Ph
Fe
$P(CF_3)_2$
L1c, ^{31}PNMR

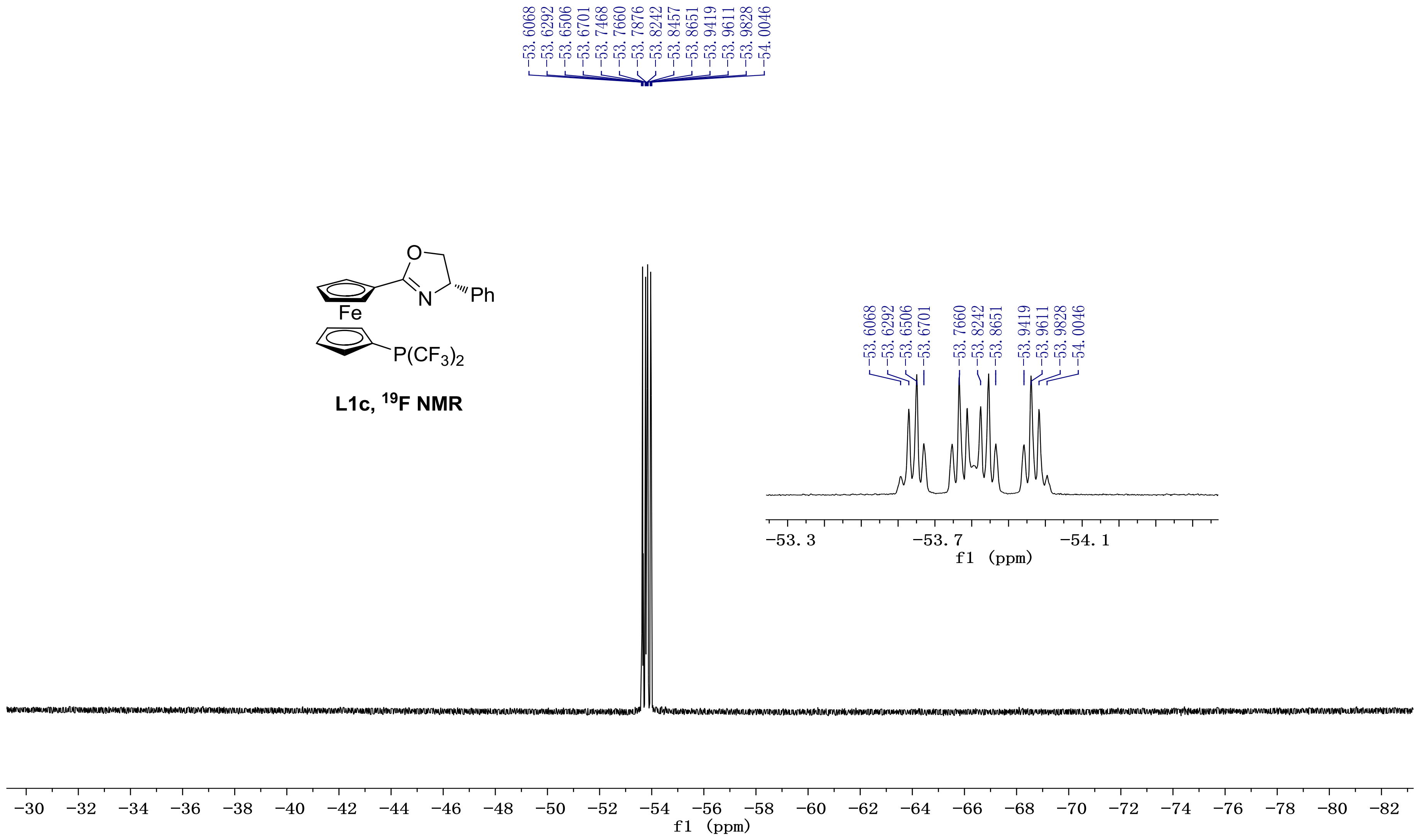

-53.6068
-53.6292
-53.6506
-53.6701
-53.7468
-53.7660
-53.7876
-53.8242
-53.8457
-53.8651
-53.9419
-53.9611
-53.9828
-54.0046
Fe
O
N
Ph
P(CF3)2
L1c, 19F NMR
-53.3
-53.7
-54.1
f1 (ppm)
-30 -32 -34 -36 -38 -40 -42 -44 -46 -48 -50 -52 -54 -56 -58 -60 -62 -64 -66 -68 -70 -72 -74 -76 -78 -80 -82
f1 (ppm)

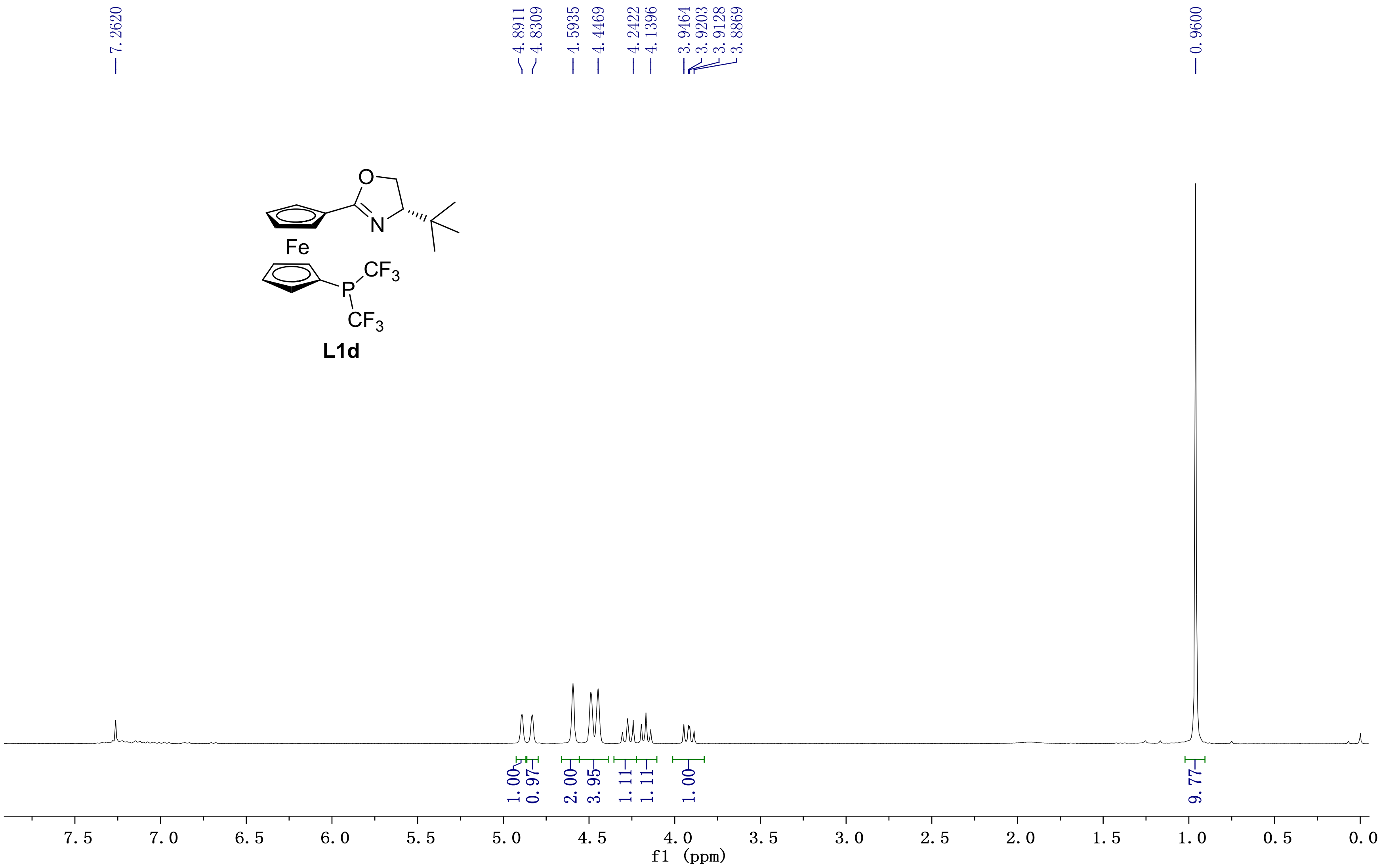
7.2620
4.8911
4.8309
4.5935
4.4469
4.2422
4.1396
3.9464
3.9203
3.9128
3.8869
0.9600
O
N
Fe
P
CF3
CF3
1.00
0.97
2.00
3.95
1.11
1.11
1.00
9.77
7.5
7.0
6.5
6.0
5.5
5.0
4.5
4.0
3.5
3.0
2.5
2.0
1.5
1.0
0.5
0.0
f1 (ppm)

L1d

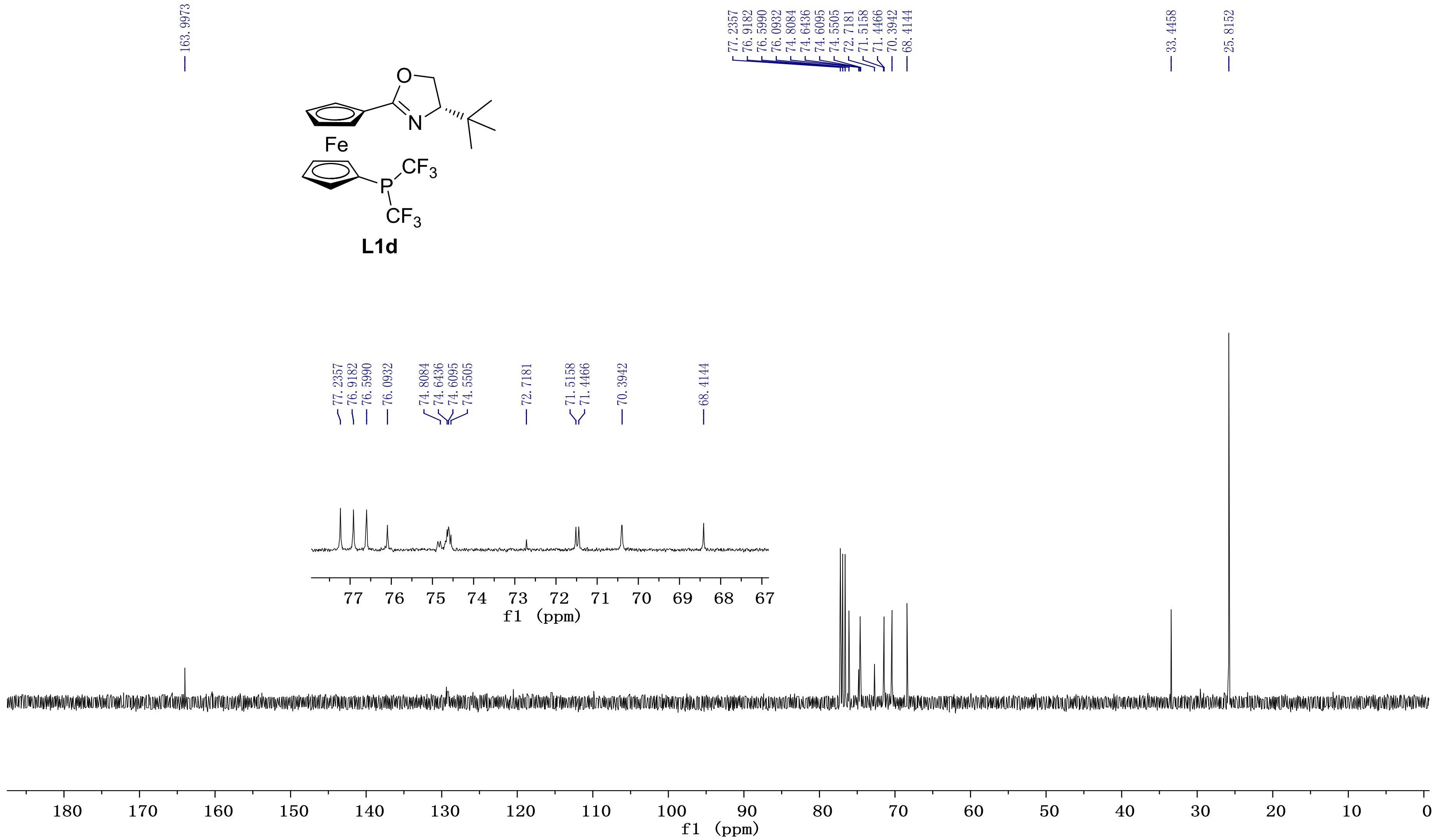

163.9973
77.2357
76.9182
76.5990
76.0932
74.8084
74.6436
74.6095
74.5505
72.7181
71.5158
71.4466
70.3942
68.4144
33.4458
25.8152
O
N
Fe
P
CF3
CF3
L1d
77 76 75 74 73 72 71 70 69 68 67
f1 (ppm)
180 170 160 150 140 130 120 110 100 90 80 70 60 50 40 30 20 10 0
f1 (ppm)

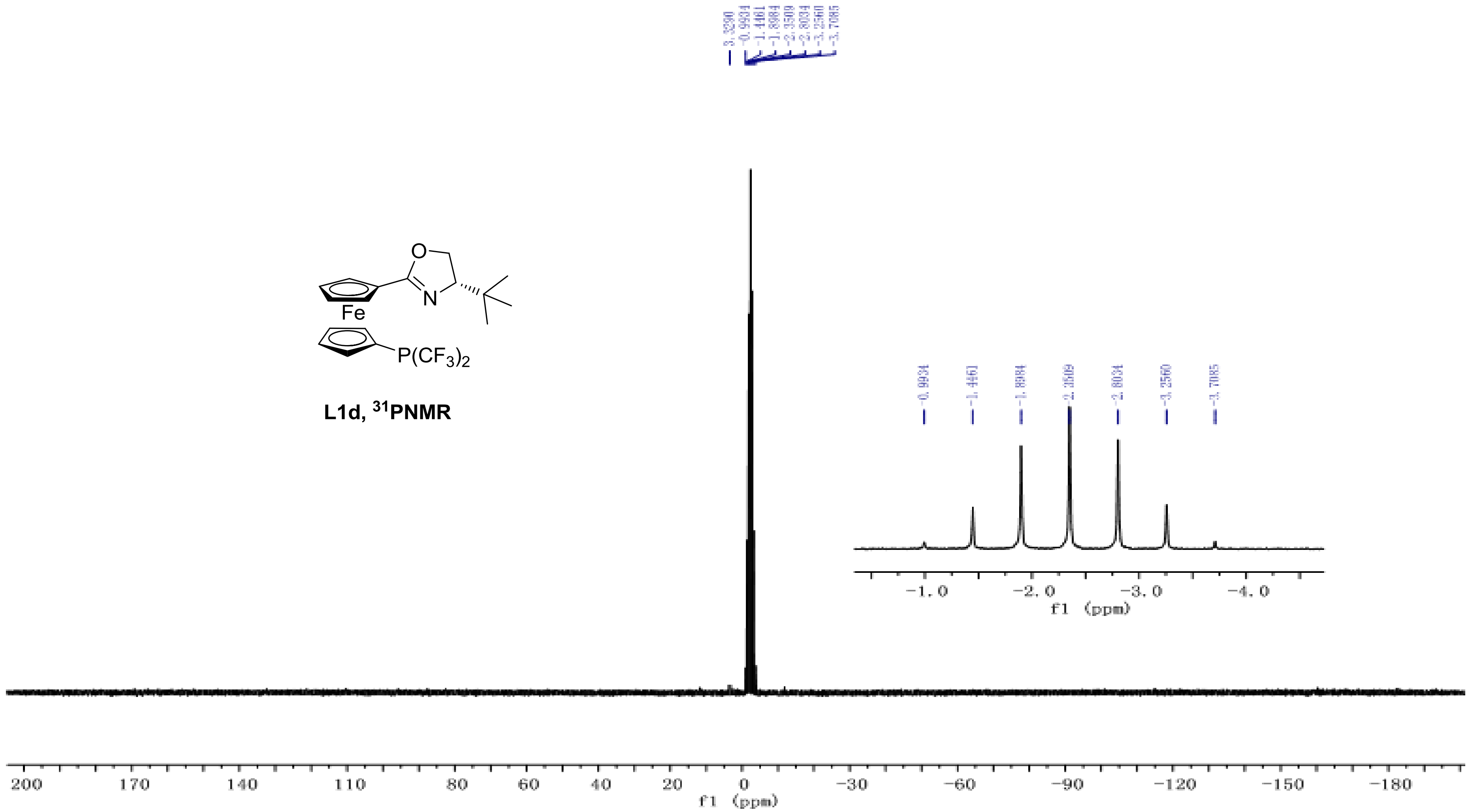

3.3290
-0.9934
-1.4461
-1.8984
-2.3509
-2.8034
-3.2560
-3.7085
Fe
O
N
P(CF3)2
L1d, 31PNMR
-1.0
-2.0
-3.0
-4.0
f1 (ppm)
200
170
140
110
80
60
40
20
0
-30
-60
-90
-120
-150
-180
f1 (ppm)

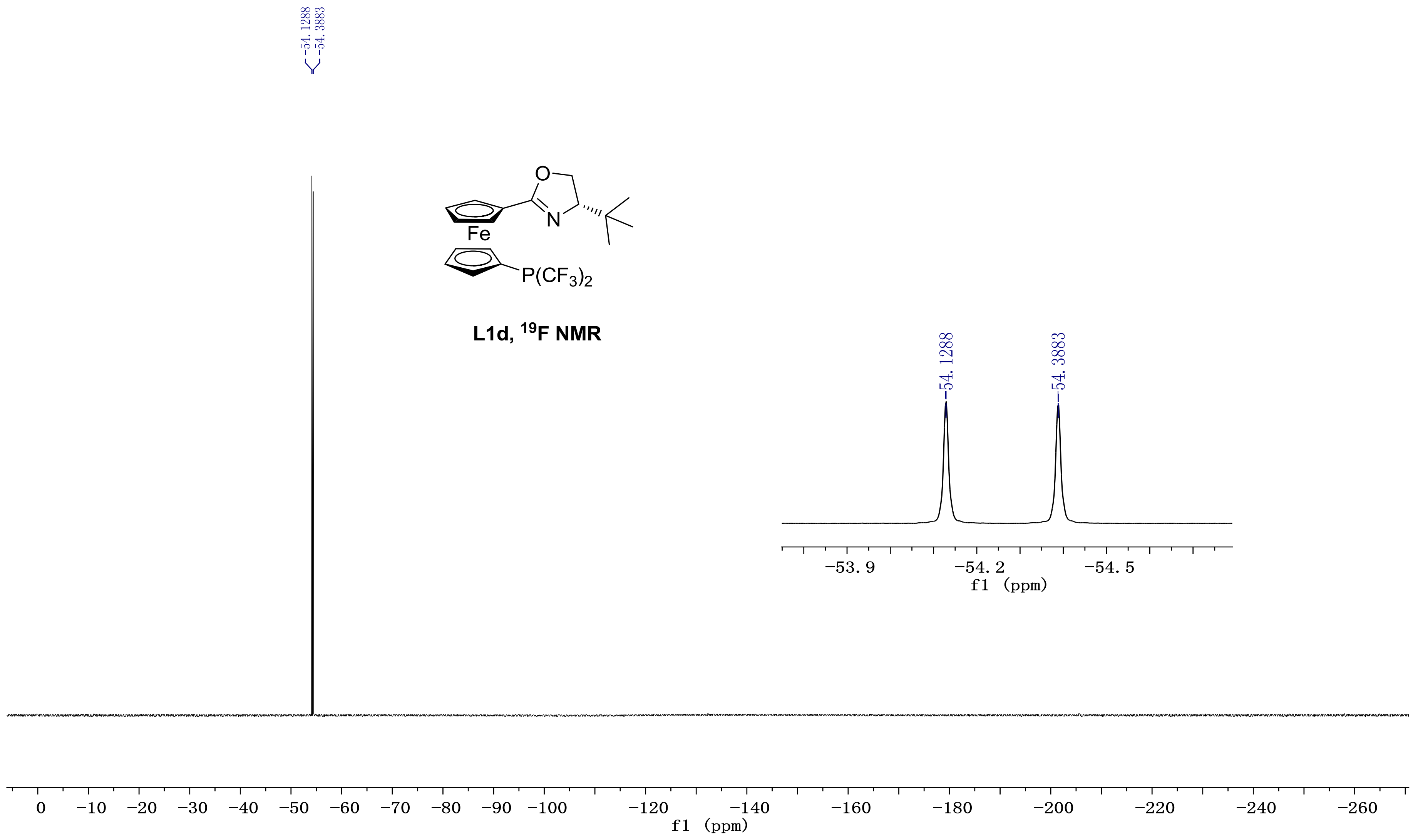

-54.1288
-54.3883
Fe
O
N
P(CF3)2
L1d, 19F NMR
-53.9
-54.2
-54.5
f1 (ppm)
0
−10
−20
−30
−40
−50
−60
−70
−80
−90
−100
−120
−140
−160
−180
−200
−220
−240
−260
f1 (ppm)

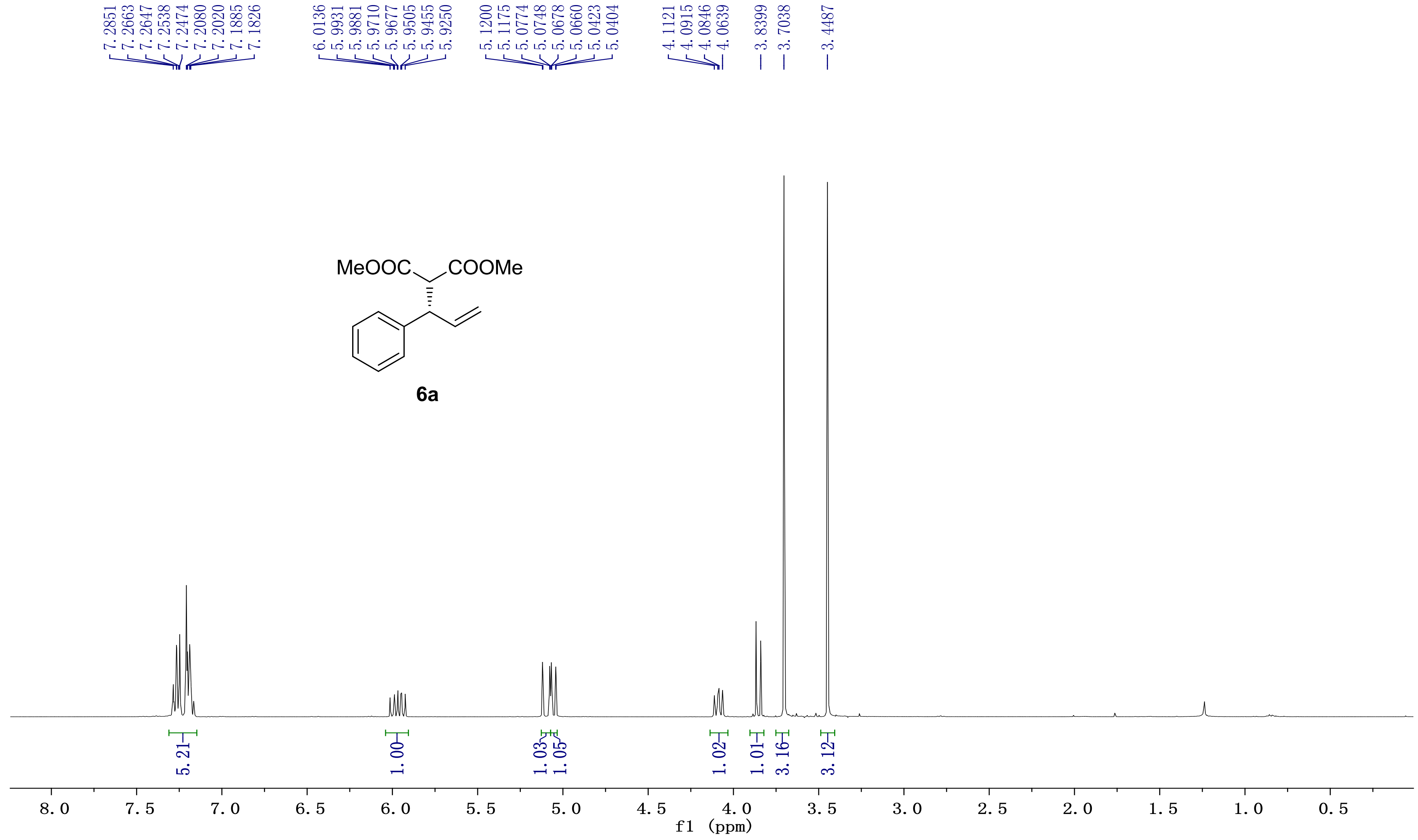
7.2851
7.2663
7.2647
7.2538
7.2474
7.2080
7.2020
7.1885
7.1826
6.0136
5.9931
5.9881
5.9710
5.9677
5.9505
5.9455
5.9250
5.1200
5.1175
5.0774
5.0748
5.0678
5.0660
5.0423
5.0404
4.1121
4.0915
4.0846
4.0639
3.8399
3.7038
3.4487
MeOOC
COOMe
6a
5.21
1.00
1.03
1.05
1.02
1.01
3.16
3.12
8.0
7.5
7.0
6.5
6.0
5.5
5.0
4.5
4.0
3.5
3.0
2.5
2.0
1.5
1.0
0.5
f1 (ppm)

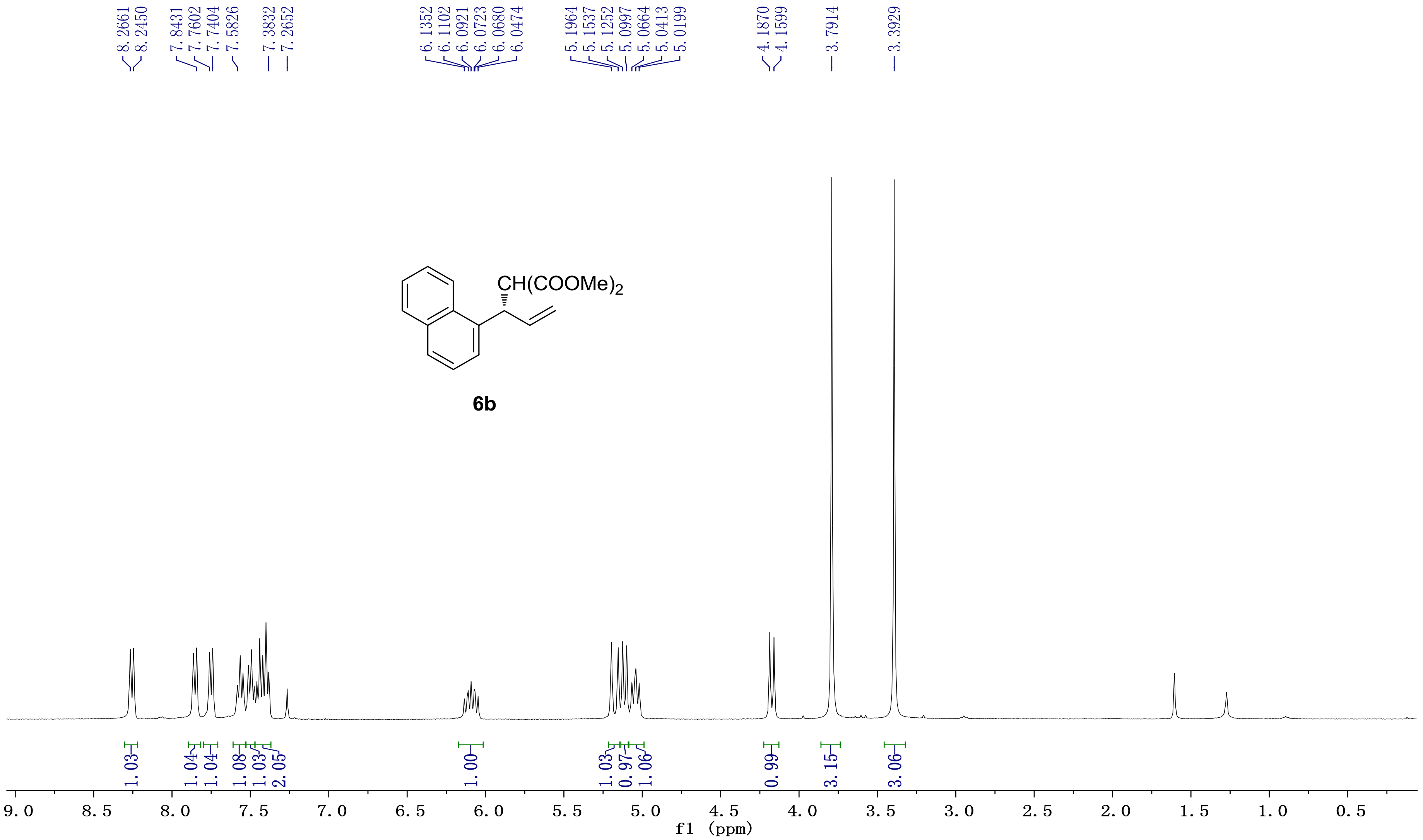
8.2661
8.2450
7.8431
7.7602
7.7404
7.5826
7.3832
7.2652
6.1352
6.1102
6.0921
6.0723
6.0680
6.0474
5.1964
5.1537
5.1252
5.0997
5.0664
5.0413
5.0199
4.1870
4.1599
3.7914
3.3929
CH(COOMe)2
6b
1.03
1.04
1.04
1.08
1.03
2.05
1.00
1.03
0.97
1.06
0.99
3.15
3.06
9.0 8.5 8.0 7.5 7.0 6.5 6.0 5.5 5.0 4.5 4.0 3.5 3.0 2.5 2.0 1.5 1.0 0.5
f1 (ppm)

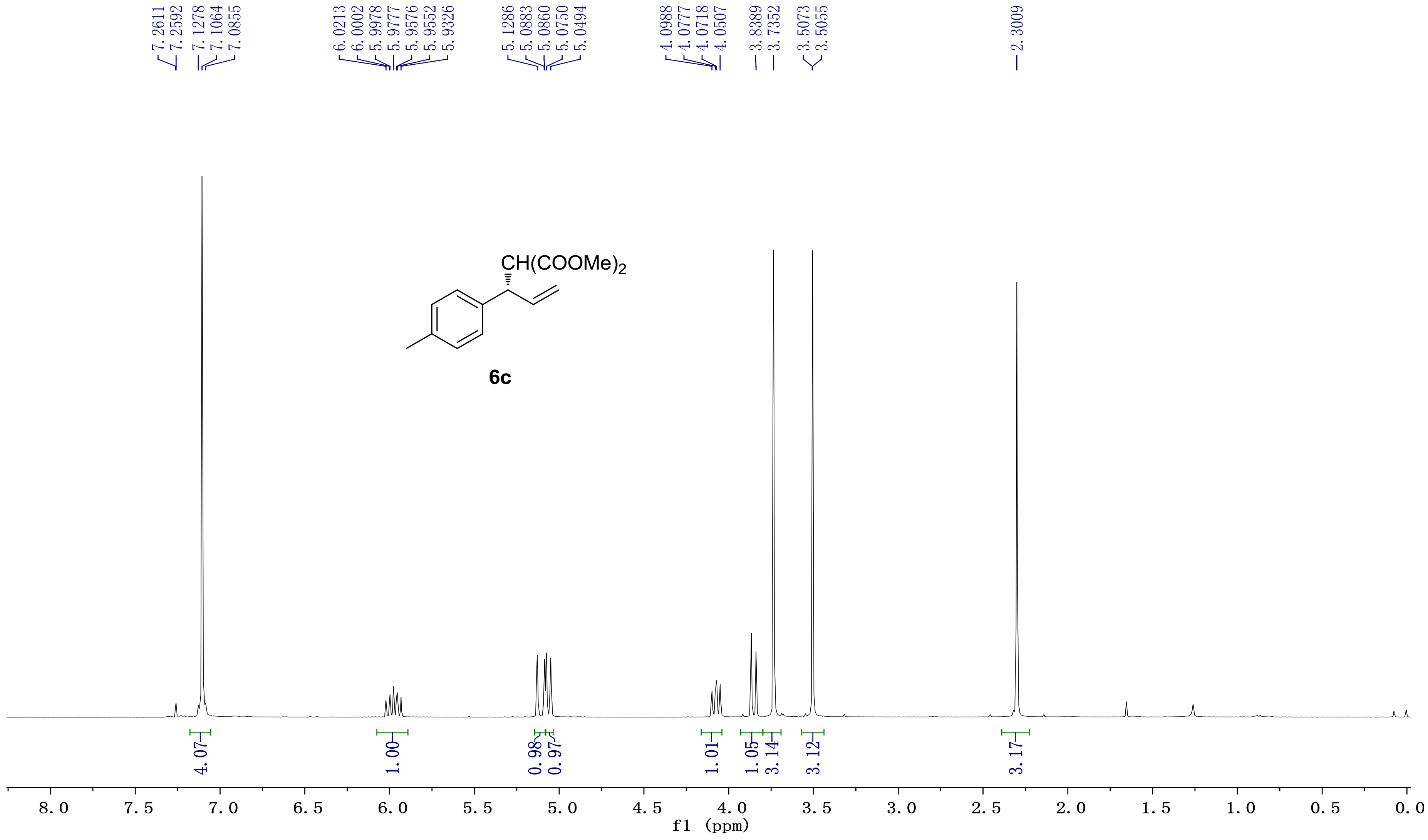
7.2611
7.2592
7.1278
7.1064
7.0855
6.0213
6.0002
5.9978
5.9777
5.9576
5.9552
5.9326
5.1286
5.0883
5.0860
5.0750
5.0494
4.0988
4.0777
4.0718
4.0507
3.8389
3.7352
3.5073
3.5055
2.3009
CH(COOMe)2
6c
4.07
1.00
0.98
0.97
1.01
1.05
3.14
3.12
3.17
8.0
7.5
7.0
6.5
6.0
5.5
5.0
4.5
4.0
3.5
3.0
2.5
2.0
1.5
1.0
0.5
0.0
f1 (ppm)

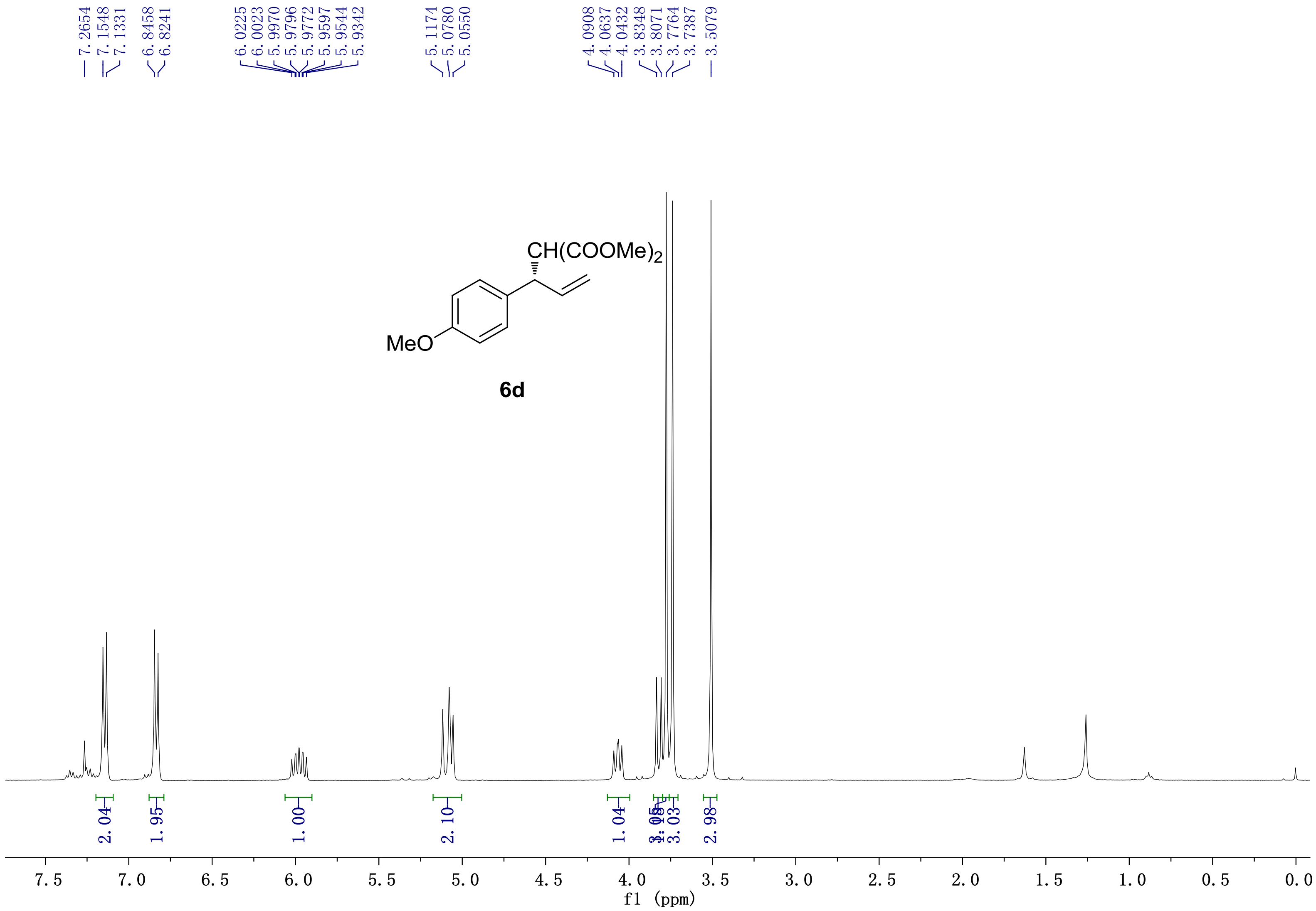
7.2654
7.1548
7.1331
6.8458
6.8241
6.0225
6.0023
5.9970
5.9796
5.9772
5.9597
5.9544
5.9342
5.1174
5.0780
5.0550
4.0908
4.0637
4.0432
3.8348
3.8071
3.7764
3.7387
3.5079
CH(COOMe)2
MeO
6d
2.04
1.95
1.00
2.10
1.04
3.03
2.98
7.5
7.0
6.5
6.0
5.5
5.0
4.5
4.0
3.5
3.0
2.5
2.0
1.5
1.0
0.5
0.0
f1 (ppm)

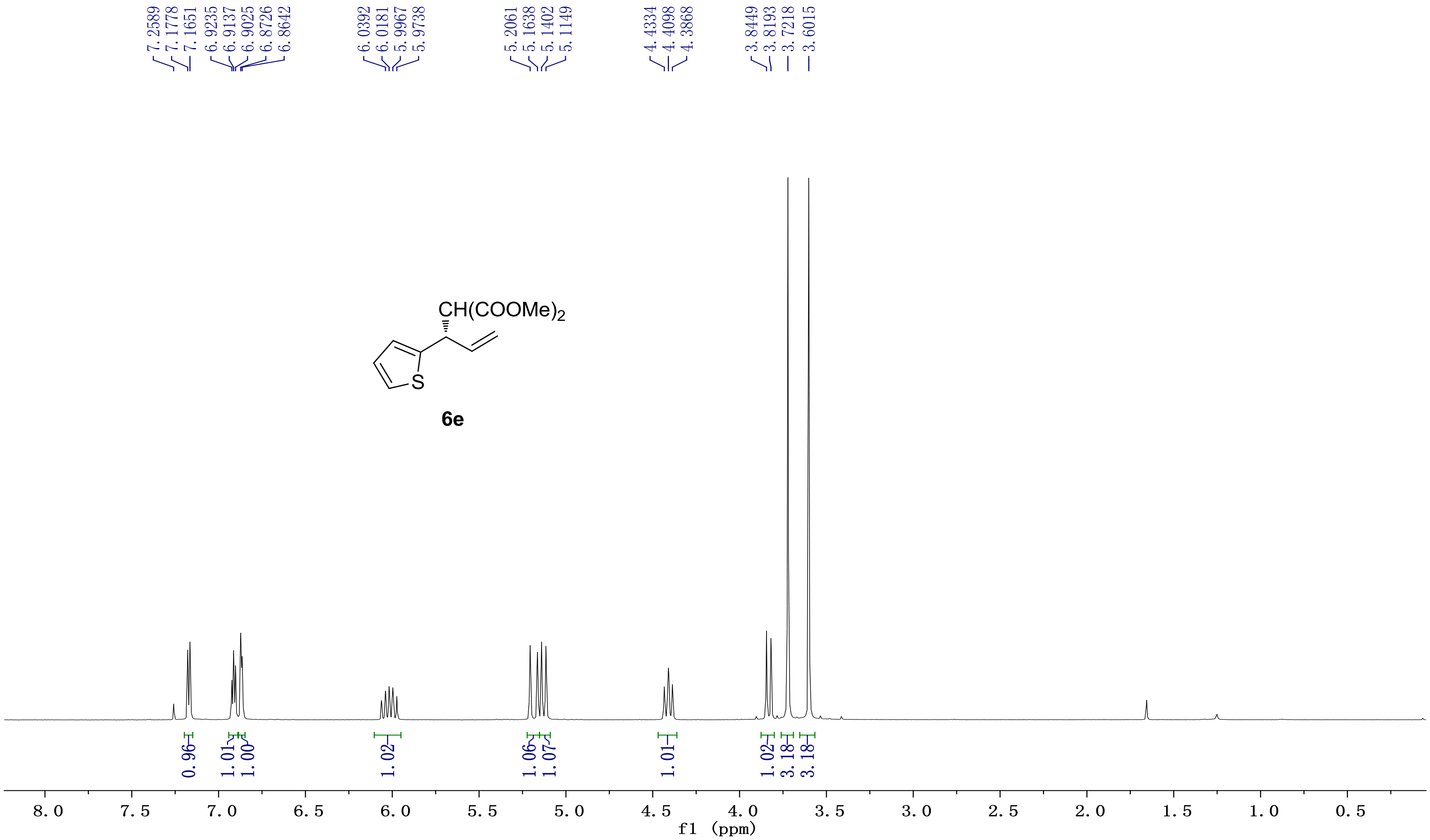
7.2589
7.1778
7.1651
6.9235
6.9137
6.9025
6.8726
6.8642
6.0392
6.0181
5.9967
5.9738
5.2061
5.1638
5.1402
5.1149
4.4334
4.4098
4.3868
3.8449
3.8193
3.7218
3.6015
CH(COOMe)2
S
0.96
1.01
1.00
1.02
1.06
1.07
1.01
1.02
3.18
3.18
8.0
7.5
7.0
6.5
6.0
5.5
5.0
4.5
4.0
3.5
3.0
2.5
2.0
1.5
1.0
0.5
f1 (ppm)

6e

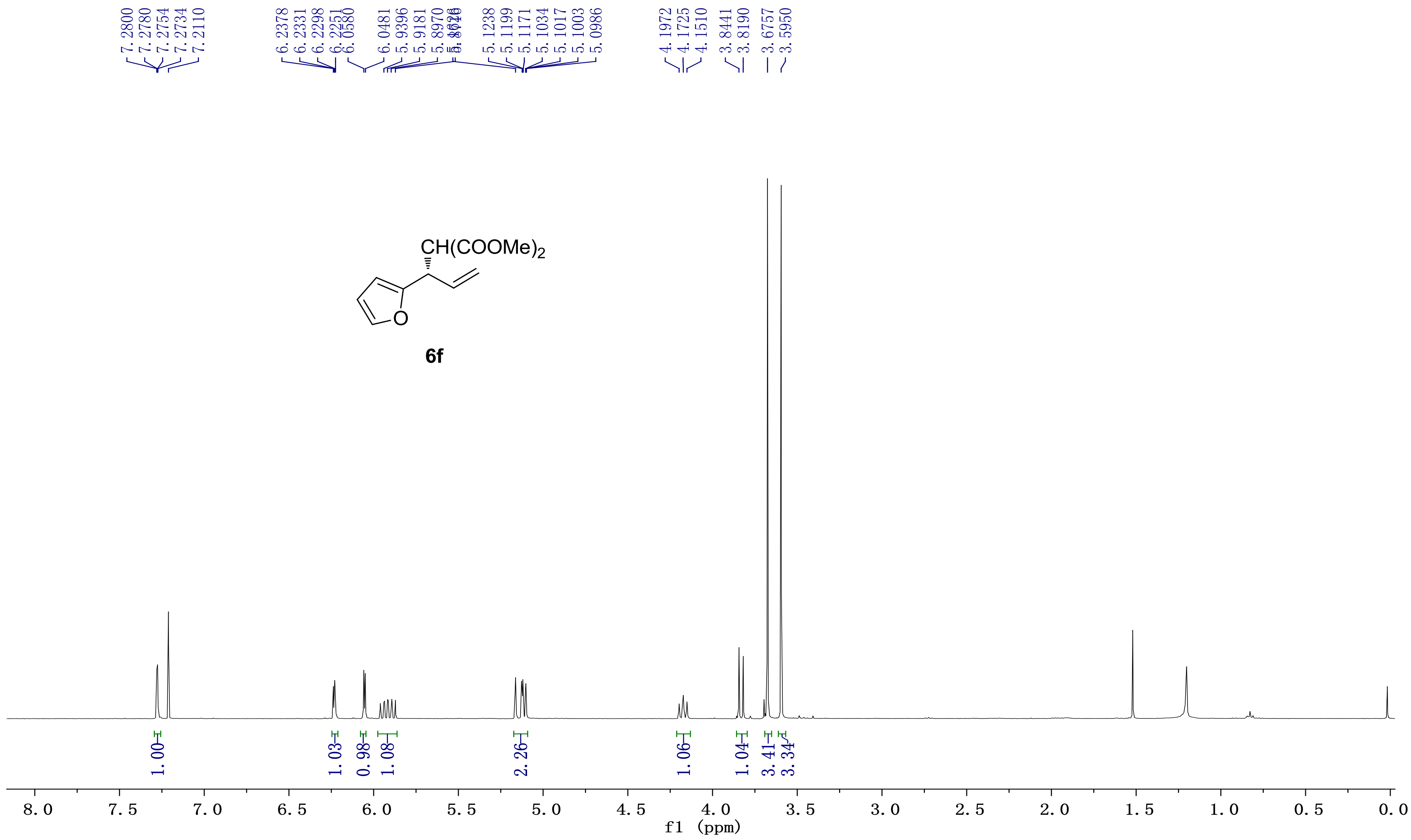
7.2800
7.2780
7.2754
7.2734
7.2110
6.2378
6.2331
6.2298
6.2251
6.0580
6.0481
5.9396
5.9181
5.8970
5.1238
5.1199
5.1171
5.1034
5.1017
5.1003
5.0986
4.1972
4.1725
4.1510
3.8441
3.8190
3.6757
3.5950
CH(COOMe)2
O
6f
1.00
1.03
0.98
1.08
2.26
1.06
1.04
3.41
3.34
8.0
7.5
7.0
6.5
6.0
5.5
5.0
4.5
4.0
3.5
3.0
2.5
2.0
1.5
1.0
0.5
0.0
f1 (ppm)

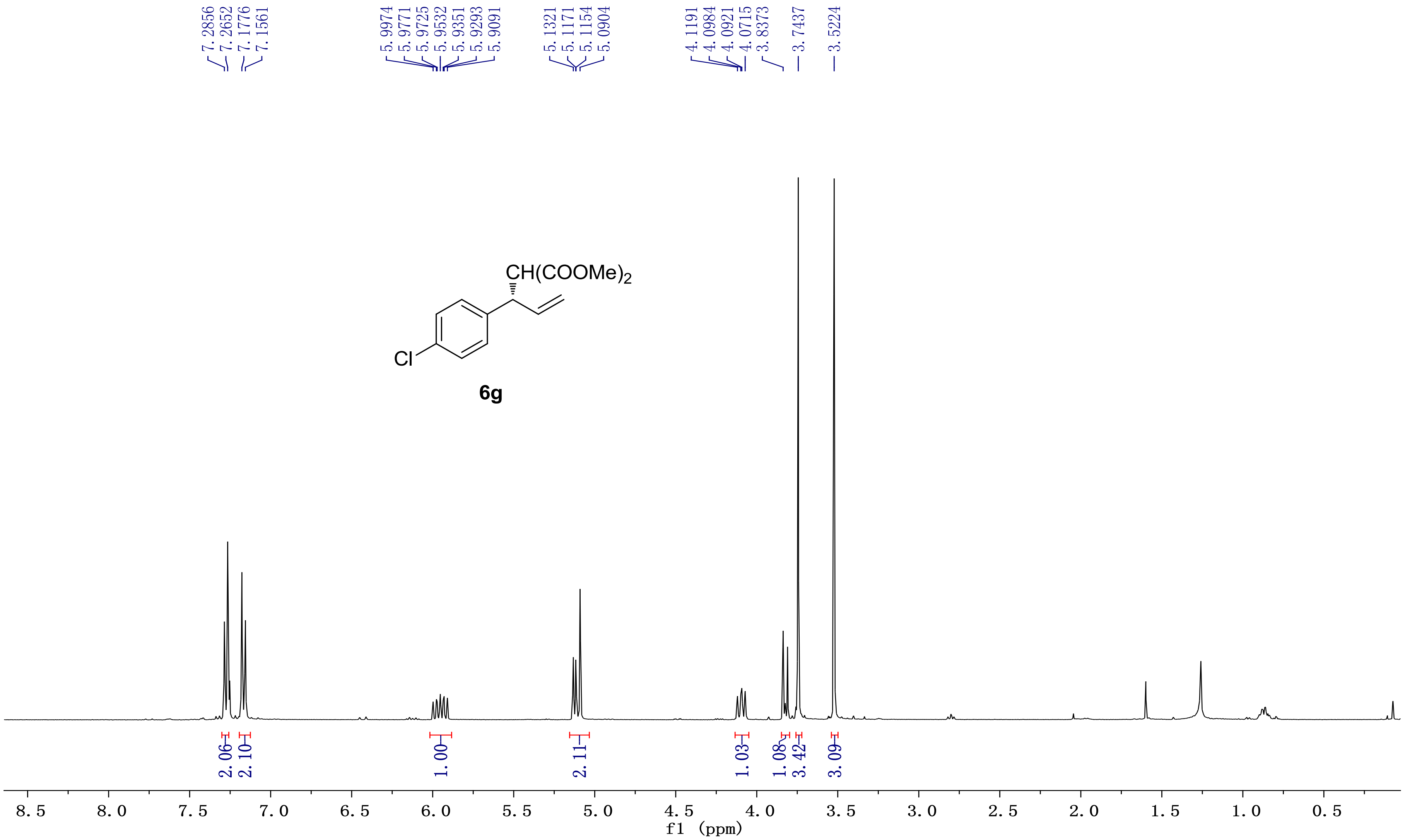
7.2856
7.2652
7.1776
7.1561
5.9974
5.9771
5.9725
5.9532
5.9351
5.9293
5.9091
5.1321
5.1171
5.1154
5.0904
4.1191
4.0984
4.0921
4.0715
3.8373
3.7437
3.5224
CH(COOMe)2
Cl
6g
2.06
2.10
1.00
2.11
1.03
1.08
3.42
3.09
8.5
8.0
7.5
7.0
6.5
6.0
5.5
5.0
4.5
4.0
3.5
3.0
2.5
2.0
1.5
1.0
0.5
f1 (ppm)

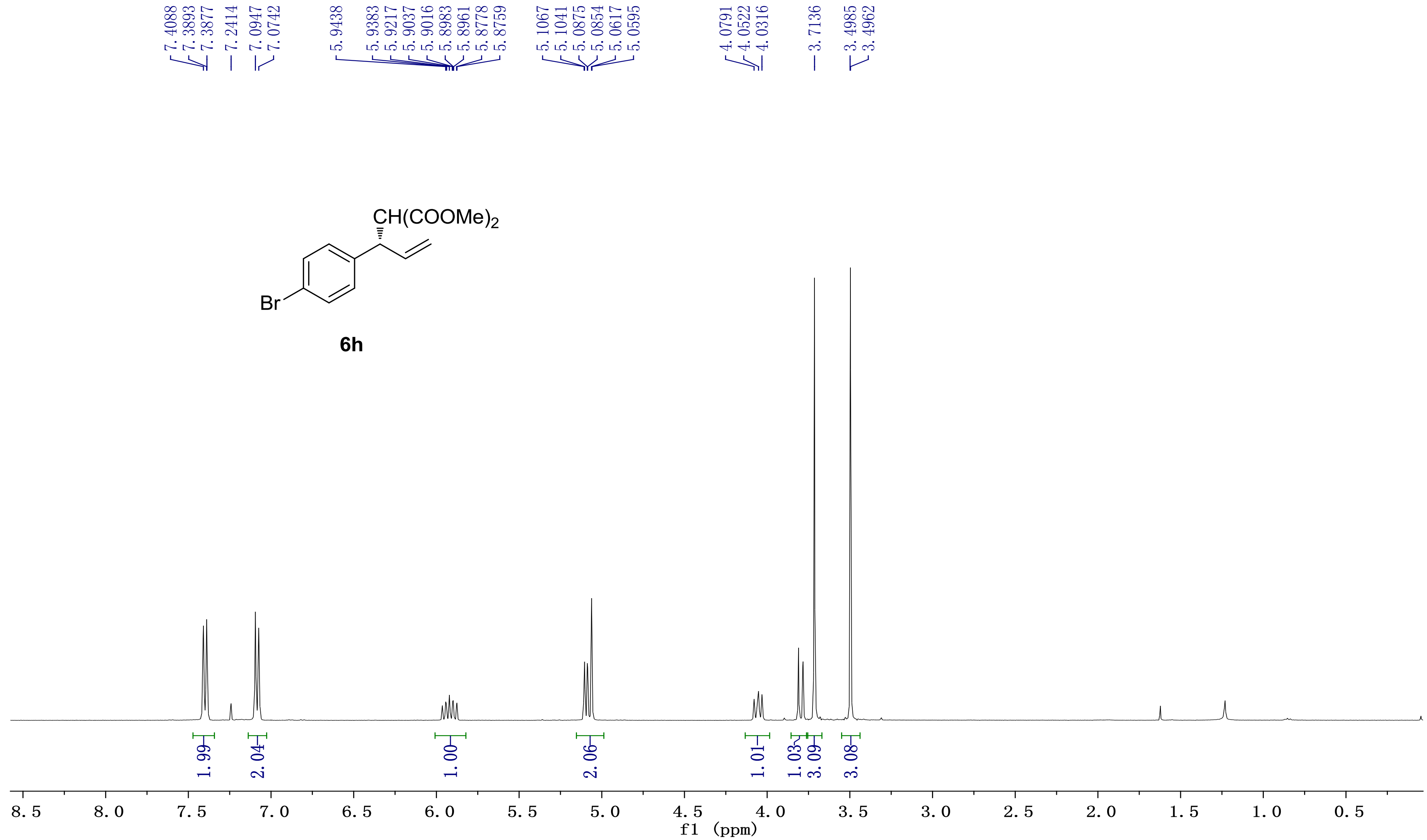
7.4088
7.3893
7.3877
7.2414
7.0947
7.0742
5.9438
5.9383
5.9217
5.9037
5.9016
5.8983
5.8961
5.8778
5.8759
5.1067
5.1041
5.0875
5.0854
5.0617
5.0595
4.0791
4.0522
4.0316
3.7136
3.4985
3.4962
CH(COOMe)2
Br
6h
1.99
2.04
1.00
2.06
1.01
1.03
3.09
3.08
8.5
8.0
7.5
7.0
6.5
6.0
5.5
5.0
4.5
4.0
3.5
3.0
2.5
2.0
1.5
1.0
0.5
f1 (ppm)

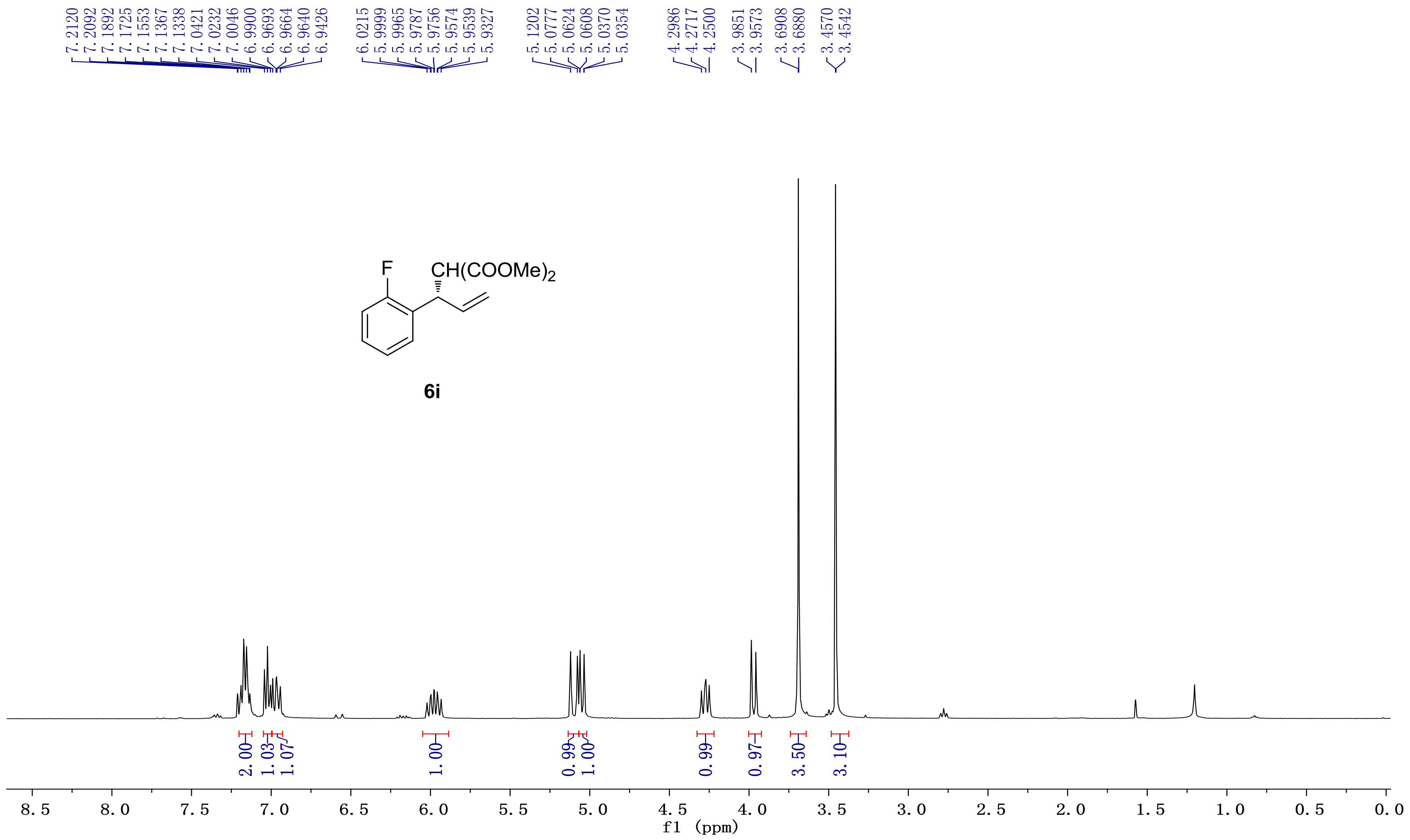

7.2120
7.2092
7.1892
7.1725
7.1553
7.1367
7.1338
7.0421
7.0232
7.0046
6.9900
6.9693
6.9664
6.9640
6.9426
6.0215
5.9999
5.9965
5.9787
5.9756
5.9574
5.9539
5.9327
5.1202
5.0777
5.0624
5.0608
5.0370
5.0354
4.2986
4.2717
4.2500
3.9851
3.9573
3.6908
3.6880
3.4570
3.4542
F
CH(COOMe)2
6i
2.00
1.03
1.07
1.00
0.99
1.00
0.99
0.97
3.50
3.10
8.5
8.0
7.5
7.0
6.5
6.0
5.5
5.0
4.5
4.0
3.5
3.0
2.5
2.0
1.5
1.0
0.5
0.0
f1 (ppm)

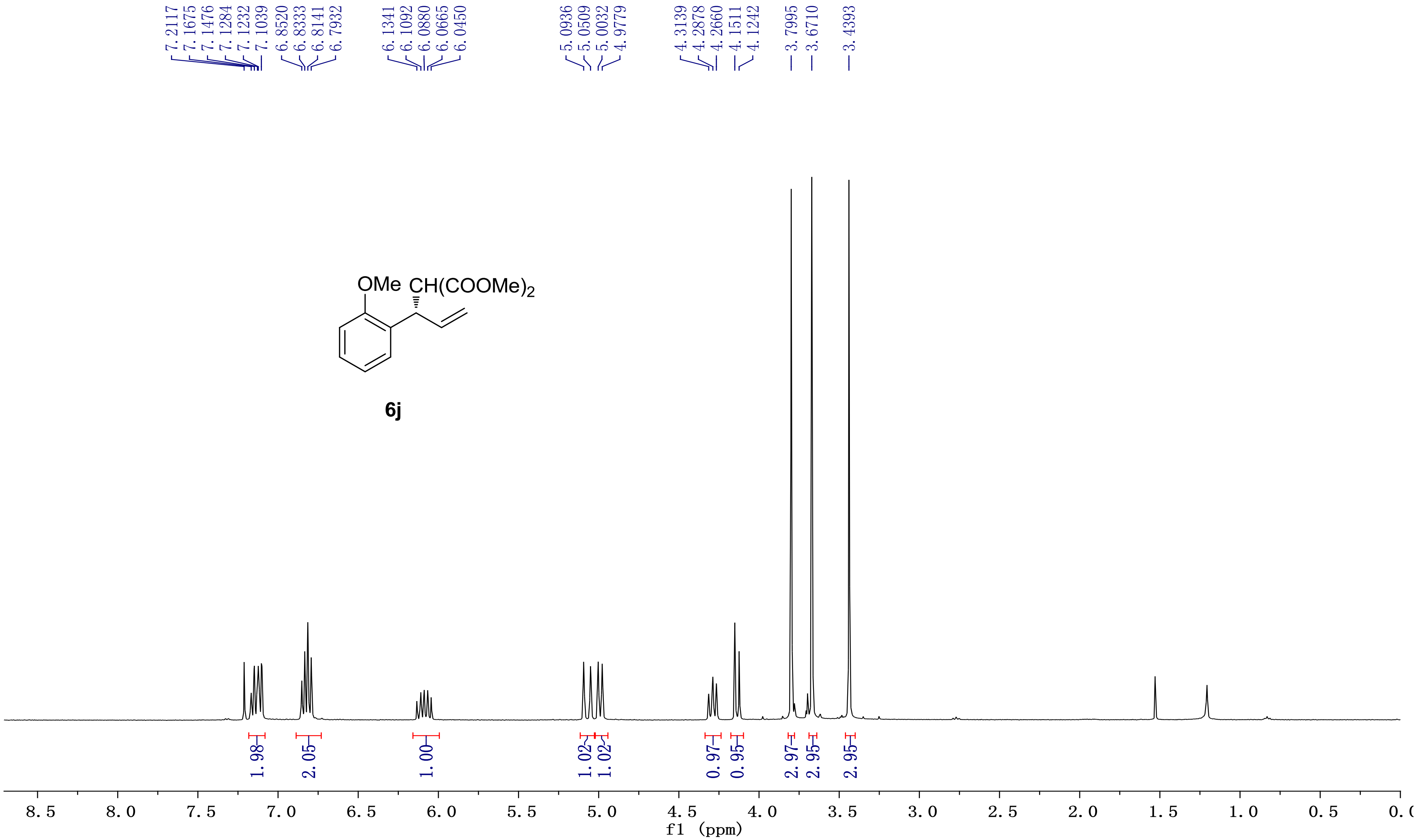
OMe
CH(COOMe)2
6j
7.2117
7.1675
7.1476
7.1284
7.1232
7.1039
6.8520
6.8333
6.8141
6.7932
6.1341
6.1092
6.0880
6.0665
6.0450
5.0936
5.0509
5.0032
4.9779
4.3139
4.2878
4.2660
4.1511
4.1242
3.7995
3.6710
3.4393
1.98
2.05
1.00
1.02
1.02
0.97
0.95
2.97
2.95
2.95
8.5
8.0
7.5
7.0
6.5
6.0
5.5
5.0
4.5
4.0
3.5
3.0
2.5
2.0
1.5
1.0
0.5
f1 (ppm)

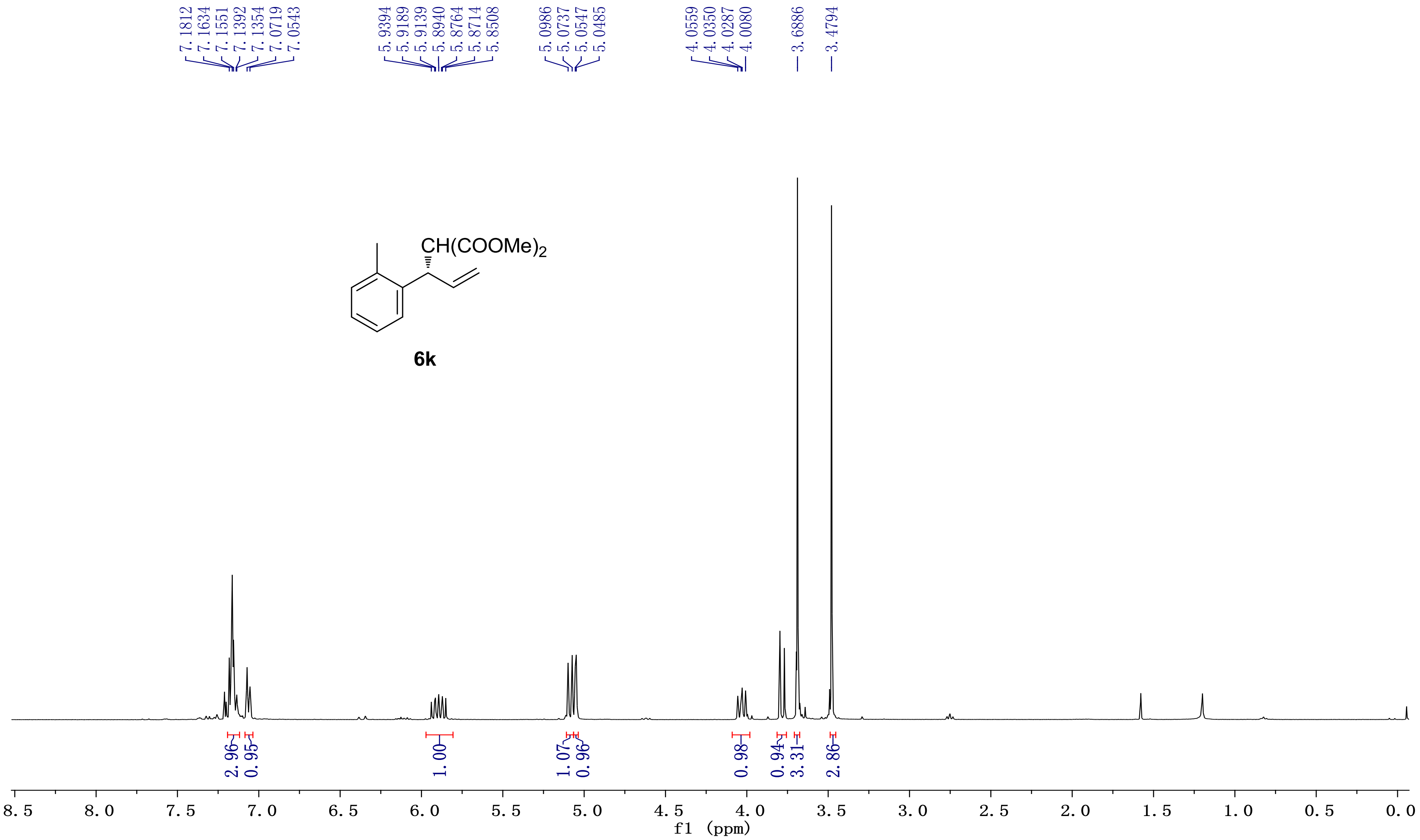
7.1812
7.1634
7.1551
7.1392
7.1354
7.0719
7.0543
5.9394
5.9189
5.9139
5.8940
5.8764
5.8714
5.8508
5.0986
5.0737
5.0547
5.0485
4.0559
4.0350
4.0287
4.0080
3.6886
3.4794
CH(COOMe)2
6k
2.96
0.95
1.00
1.07
0.96
0.98
0.94
3.31
2.86
8.5
8.0
7.5
7.0
6.5
6.0
5.5
5.0
4.5
4.0
3.5
3.0
2.5
2.0
1.5
1.0
0.5
0.0
f1 (ppm)

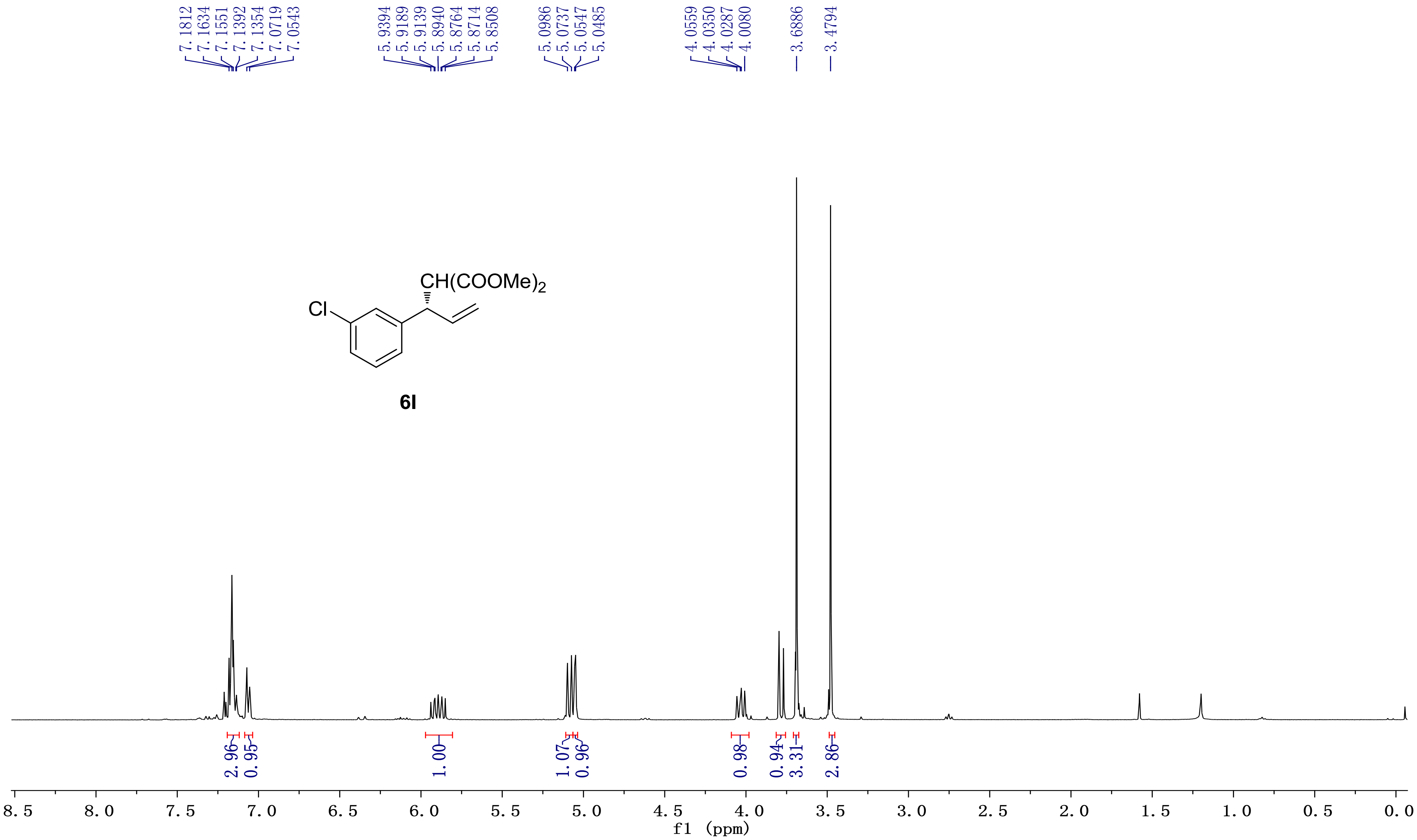

7.1812
7.1634
7.1551
7.1392
7.1354
7.0719
7.0543
5.9394
5.9189
5.9139
5.8940
5.8764
5.8714
5.8508
5.0986
5.0737
5.0547
5.0485
4.0559
4.0350
4.0287
4.0080
3.6886
3.4794
CH(COOMe)2
Cl
6l
2.96
0.95
1.00
1.07
0.96
0.98
0.94
3.31
2.86
8.5
8.0
7.5
7.0
6.5
6.0
5.5
5.0
4.5
4.0
3.5
3.0
2.5
2.0
1.5
1.0
0.5
0.0
f1 (ppm)

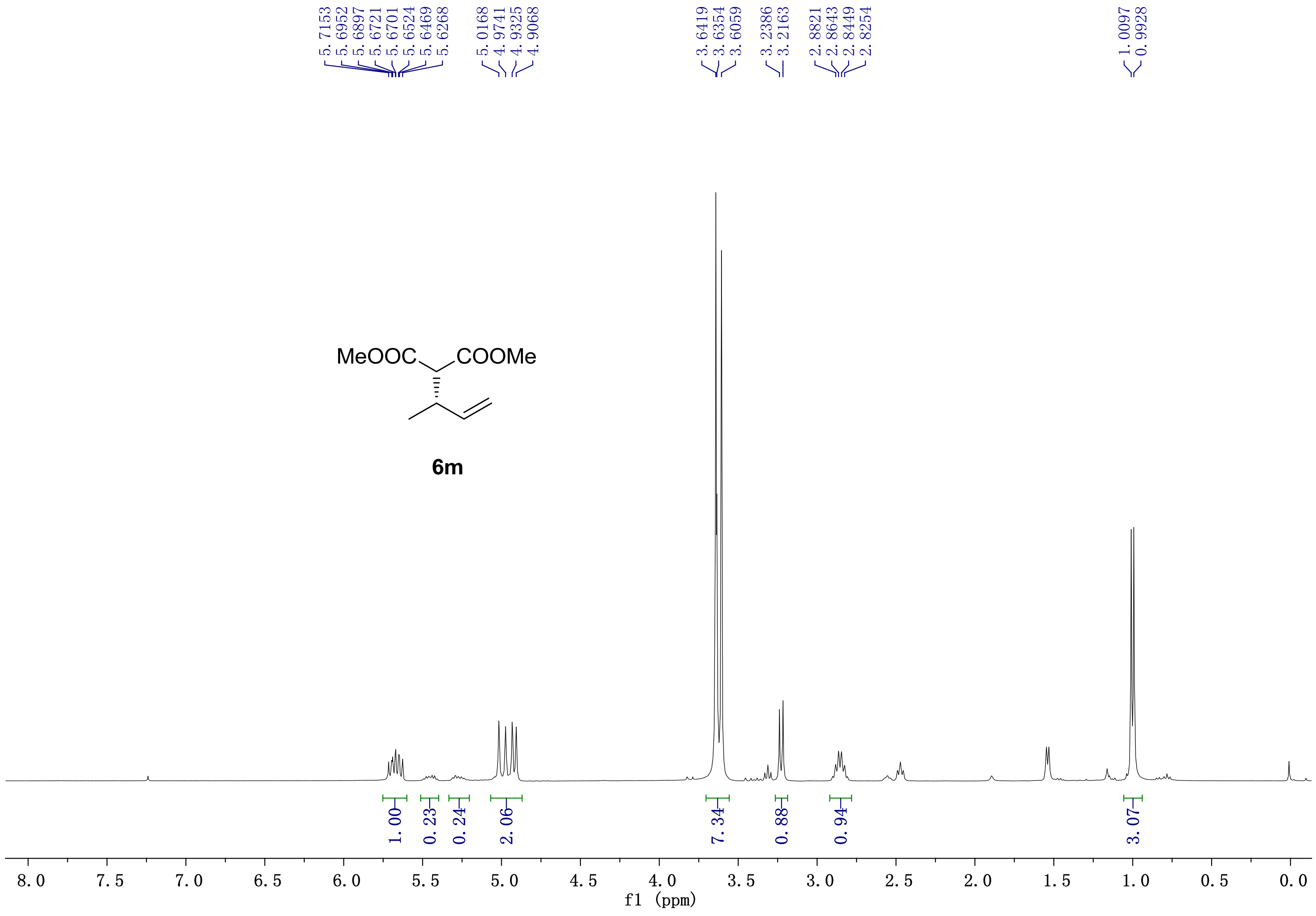
5.7153
5.6952
5.6897
5.6721
5.6701
5.6524
5.6469
5.6268
5.0168
4.9741
4.9325
4.9068
3.6419
3.6354
3.6059
3.2386
3.2163
2.8821
2.8643
2.8449
2.8254
1.0097
0.9928
MeOOC
COOMe
1.00
0.23
0.24
2.06
7.34
0.88
0.94
3.07
8.0 7.5 7.0 6.5 6.0 5.5 5.0 4.5 4.0 3.5 3.0 2.5 2.0 1.5 1.0 0.5 0.0
f1 (ppm)

6m

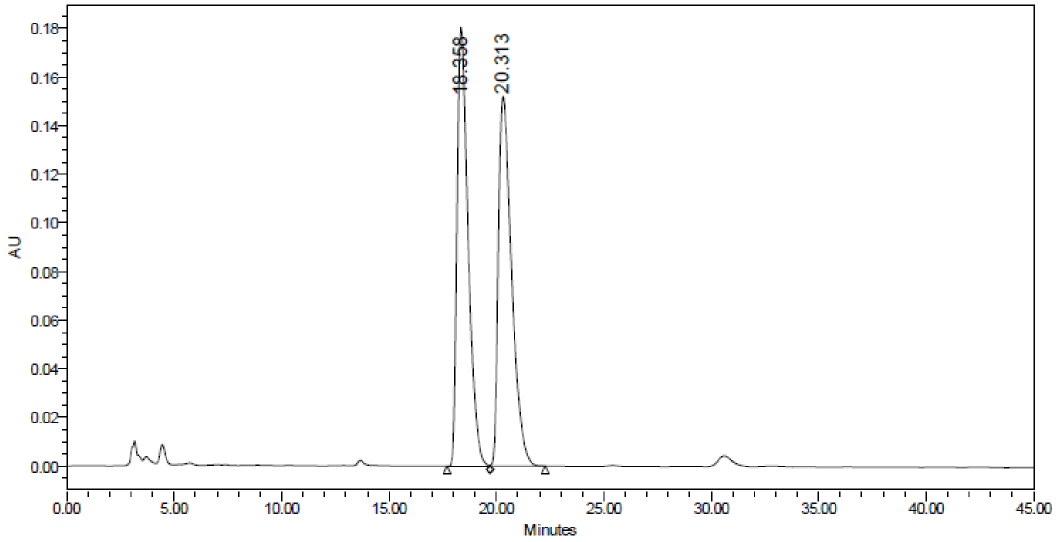

	RT (min)	Area (µV*sec)	% Area	Height (µV)	% Height
1	18.358	6475692	49.97	180624	54.31
2	20.313	6484325	50.03	151930	45.69

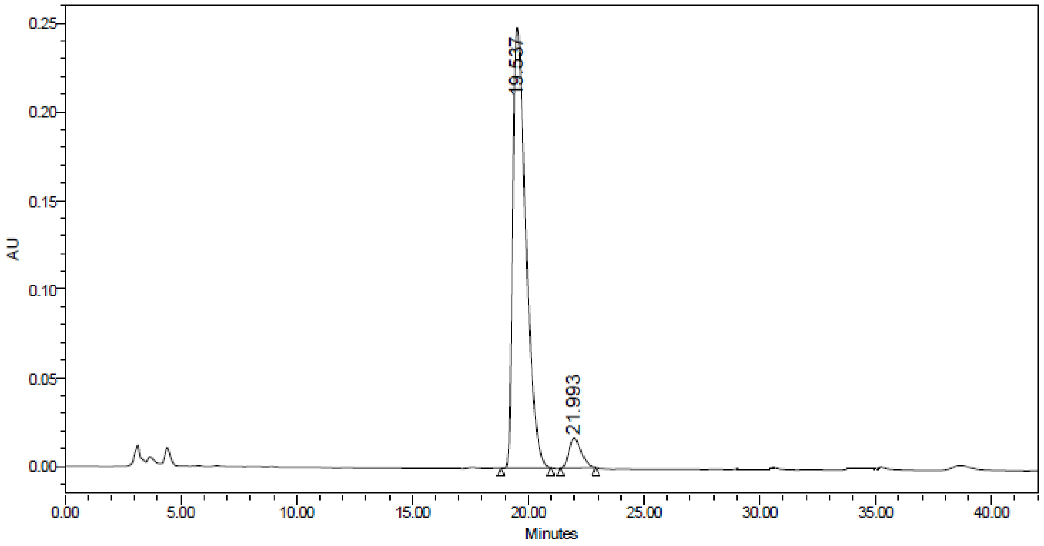

	RT (min)	Area (µV*sec)	% Area	Height (µV)	% Height
1	19.537	9738931	93.95	248498	93.73
2	21.993	626862	6.05	16626	6.27

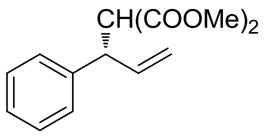

6a

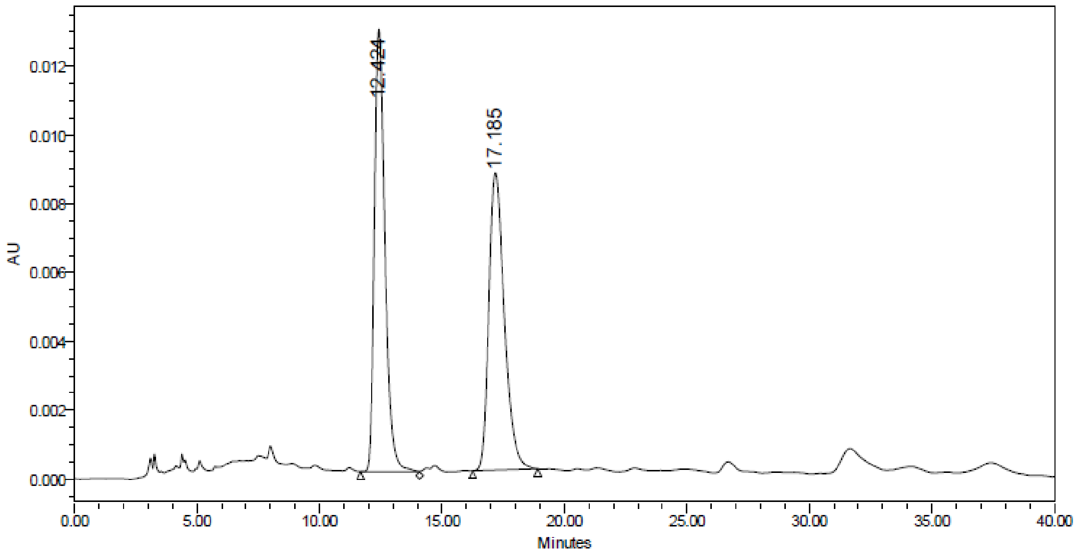

	RT (min)	Area (µV*sec)	% Area	Height (µV)	% Height
1	12.424	383706	50.28	12863	59.81
2	17.185	379418	49.72	8643	40.19

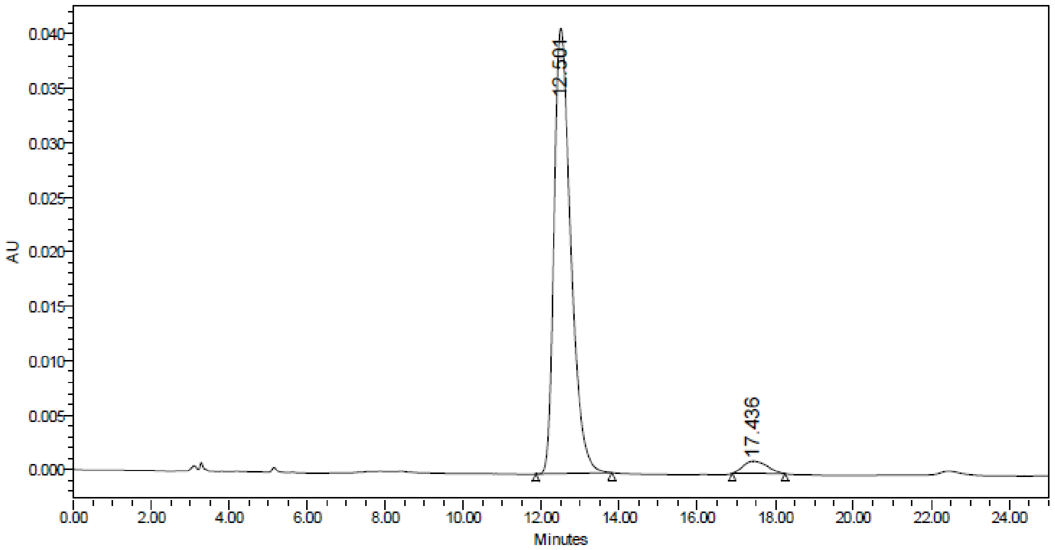

	RT (min)	Area (µV*sec)	% Area	Height (µV)	% Height
1	12.501	1214733	96.19	40874	97.33
2	17.436	48088	3.81	1122	2.67

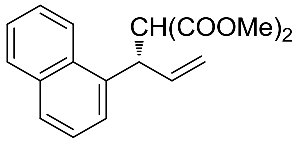

6b

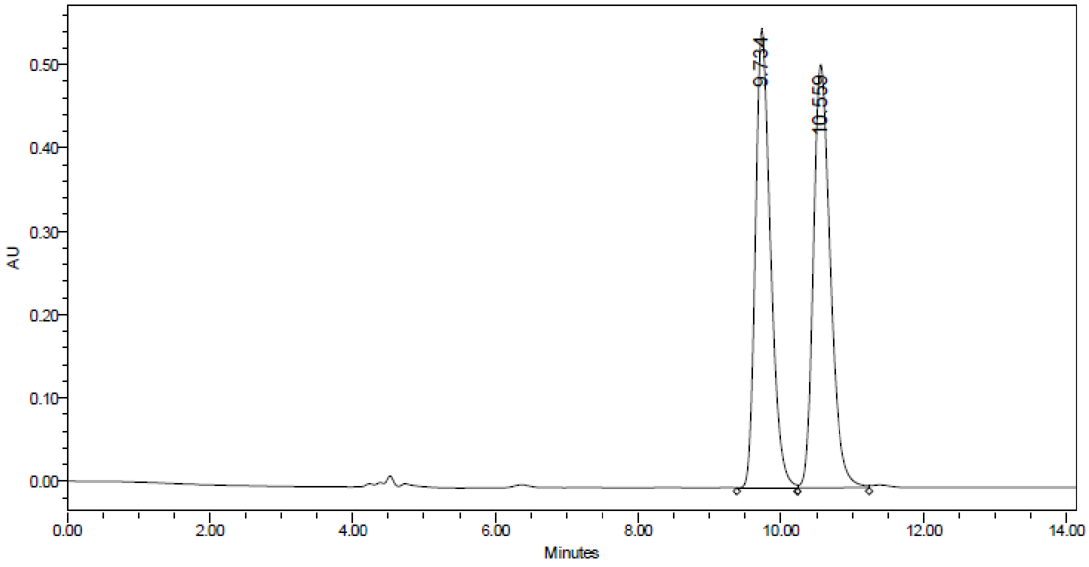

	RT (min)	Area (µV*sec)	% Area	Height (µV)	% Height
1	9.734	8229584	49.69	551394	52.03
2	10.559	8332083	50.31	508398	47.97

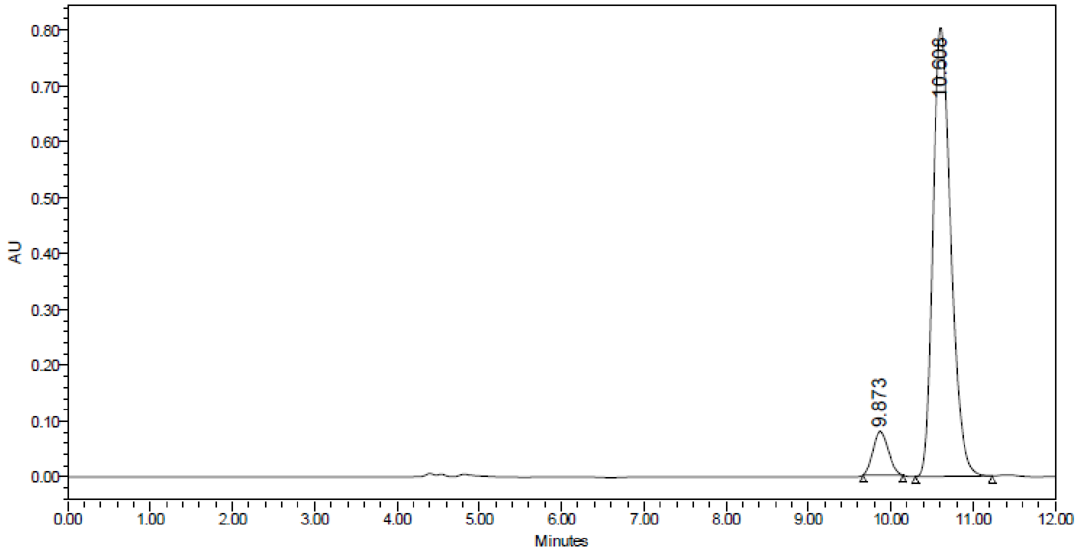

	RT (min)	Area (µV*sec)	% Area	Height (µV)	% Height
1	9.873	1011762	7.69	78231	8.85
2	10.608	12149787	92.31	805708	91.15

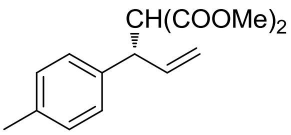

6c

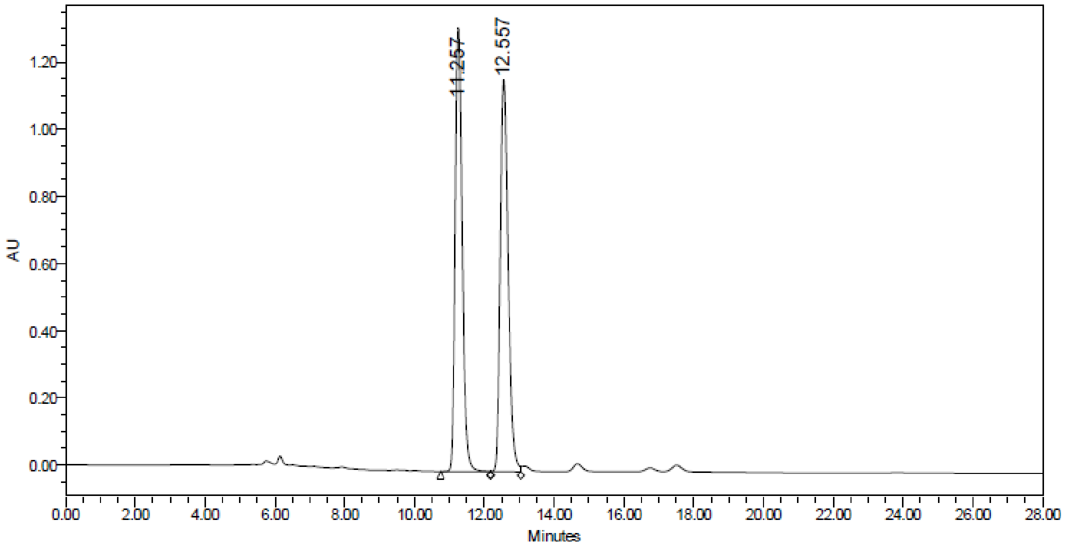

	RT (min)	Area (µV*sec)	% Area	Height (µV)	% Height
1	11.257	18267646	49.97	1325334	53.11
2	12.557	18289924	50.03	1170079	46.89

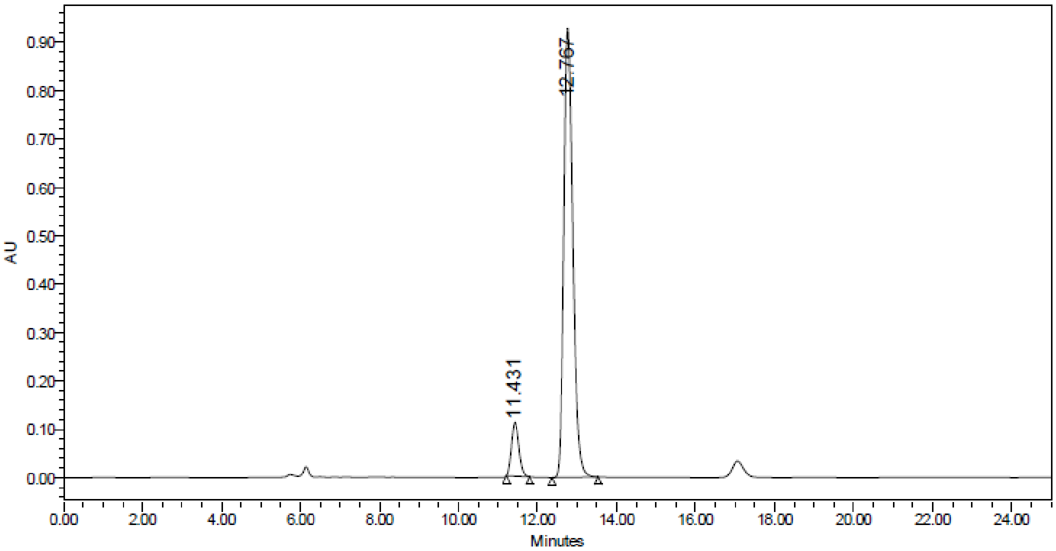

	RT (min)	Area (µV*sec)	% Area	Height (µV)	% Height
1	11.431	1458444	9.09	111225	10.70
2	12.767	14594653	90.91	928123	89.30

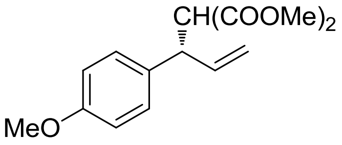

6d

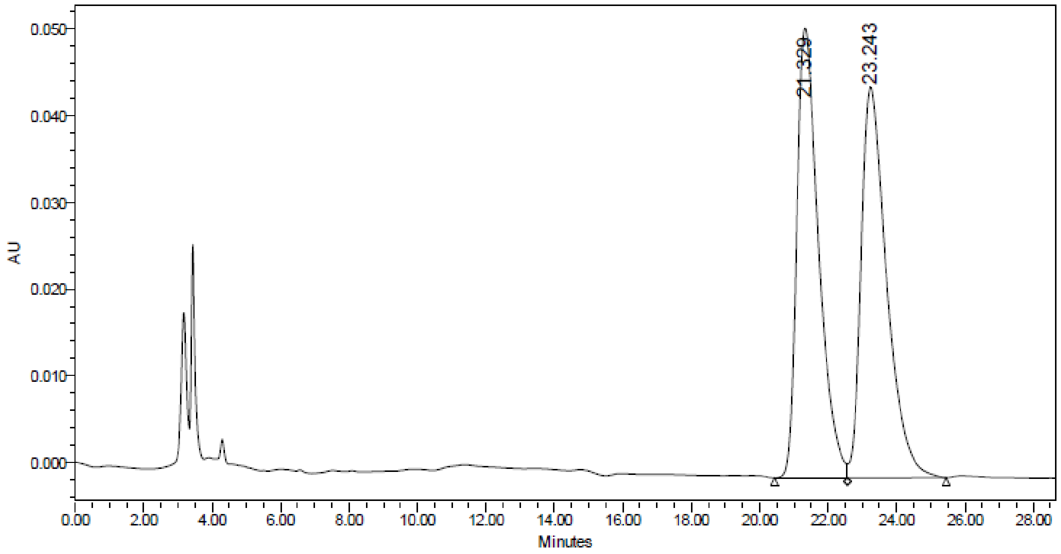

	RT (min)	Area (µV*sec)	% Area	Height (µV)	% Height
1	21.329	2303008	49.65	51878	53.52
2	23.243	2335145	50.35	45051	46.48

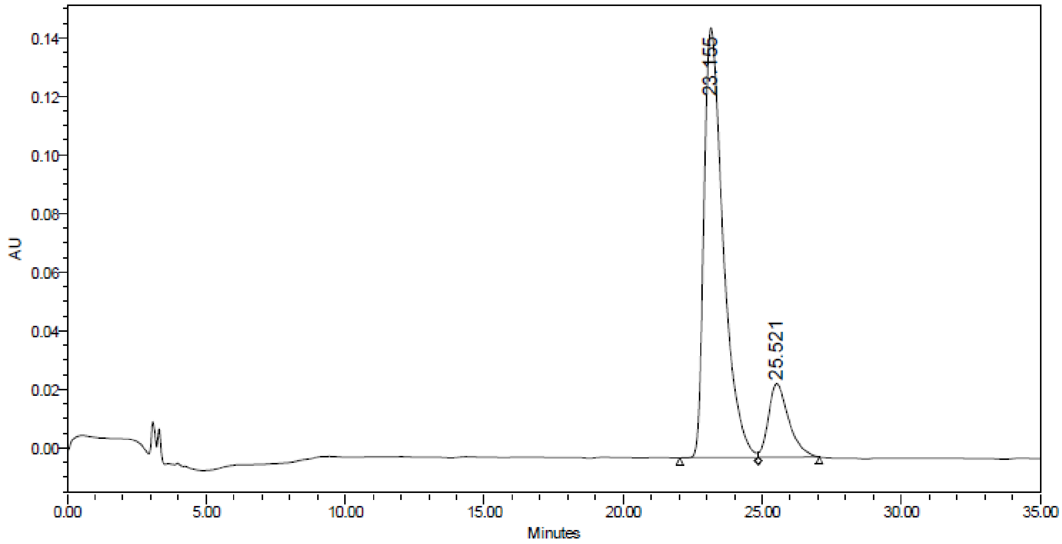

	RT (min)	Area (µV*sec)	% Area	Height (µV)	% Height
1	23.155	7066554	84.74	146840	85.43
2	25.521	1272434	15.26	25040	14.57

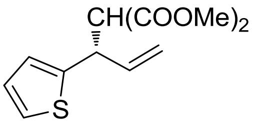

6e

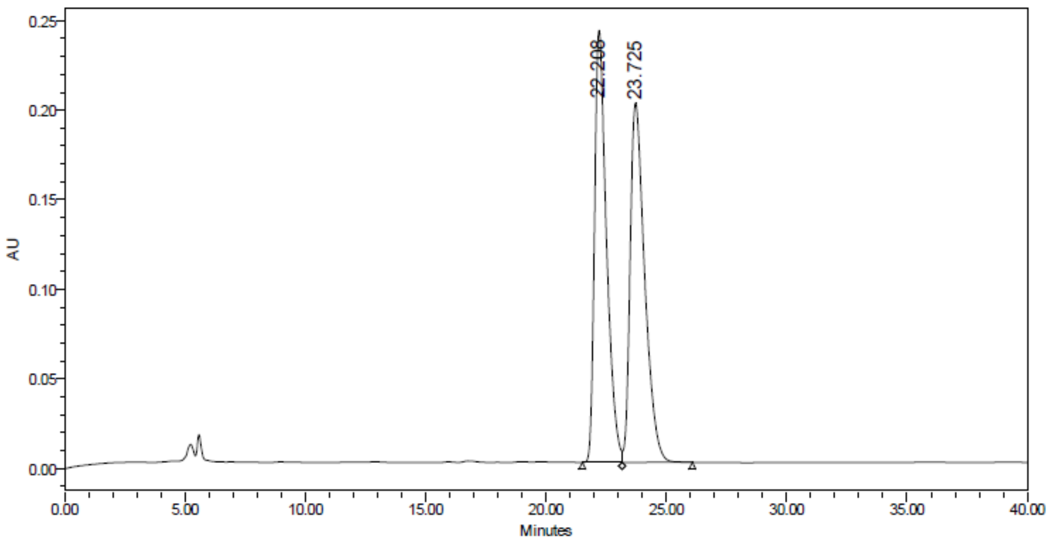

	RT (min)	Area (µV*sec)	% Area	Height (µV)	% Height
1	22.208	8300809	49.58	241382	54.61
2	23.725	8440896	50.42	200658	45.39

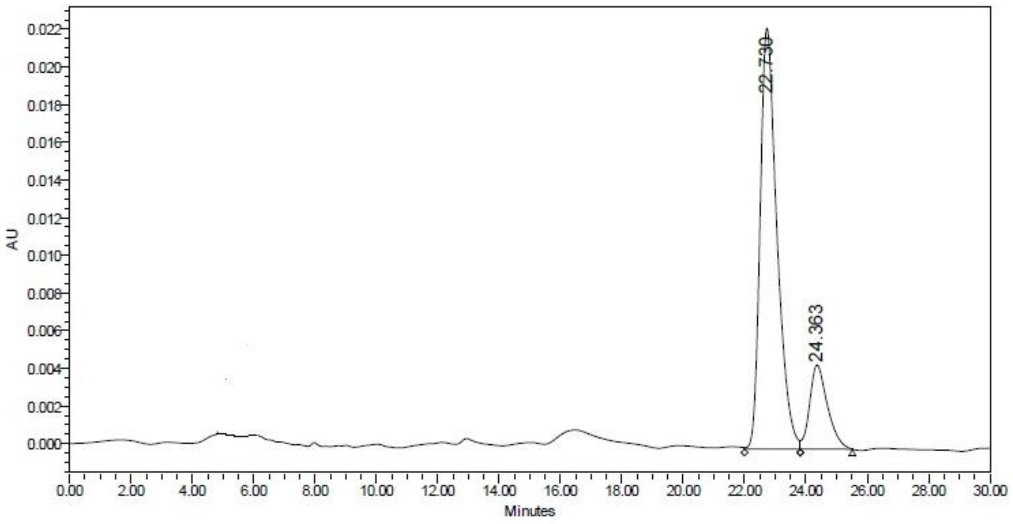

	RT (min)	Area (µV*sec)	% Area	Height (µV)	% Height
1	22.730	850027	82.46	22345	83.32
2	24.363	180865	17.54	4475	16.68

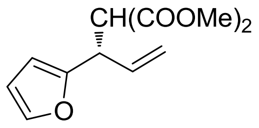

6f

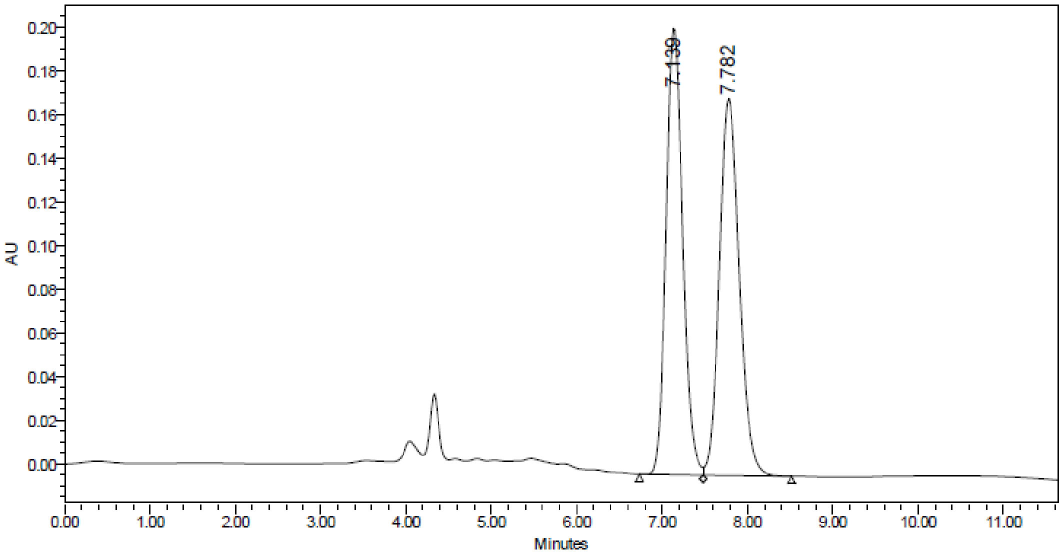

	RT (min)	Area (μV*sec)	% Area	Height (μV)	% Height
1	7.139	2771130	49.80	204622	54.26
2	7.782	2793338	50.20	172519	45.74

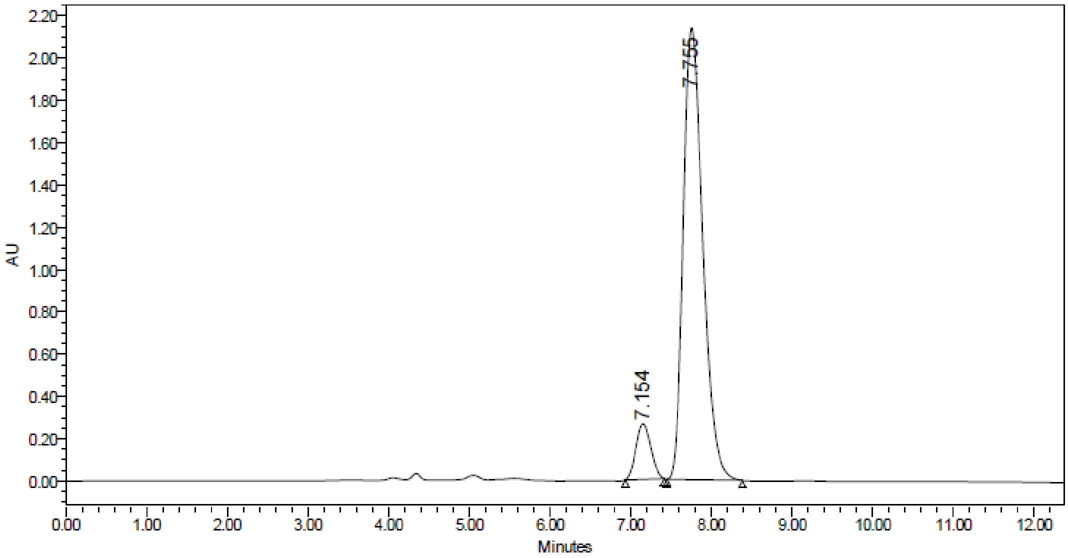

	RT (min)	Area (μV*sec)	% Area	Height (μV)	% Height
1	7.154	3352445	8.55	262725	10.95
2	7.755	35862552	91.45	2137653	89.05

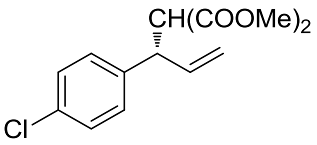

6g

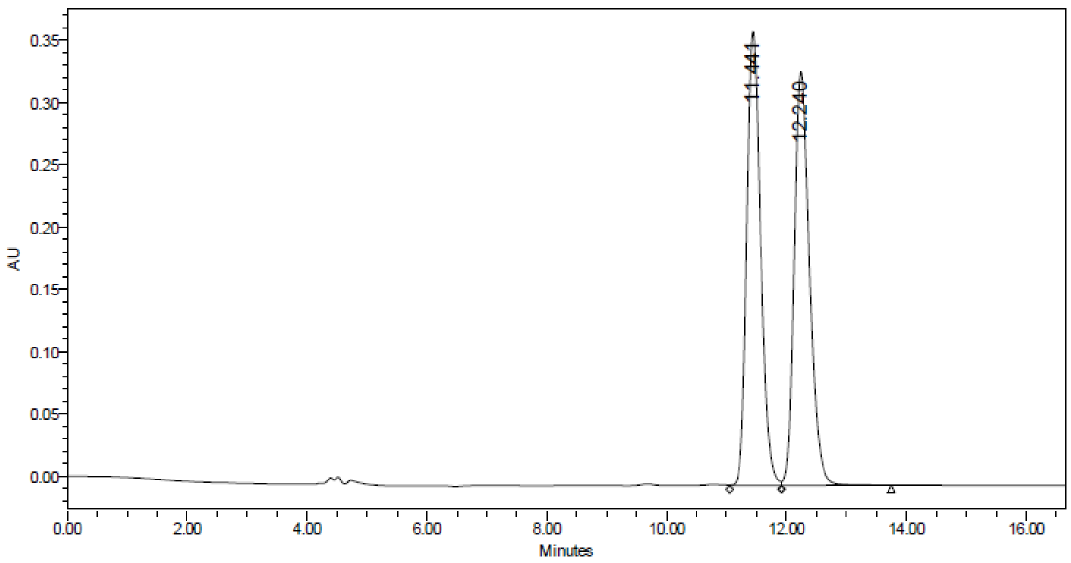

	RT (min)	Area (µV*sec)	% Area	Height (µV)	% Height
1	11.441	5891499	49.87	365237	52.35
2	12.240	5922239	50.13	332407	47.65

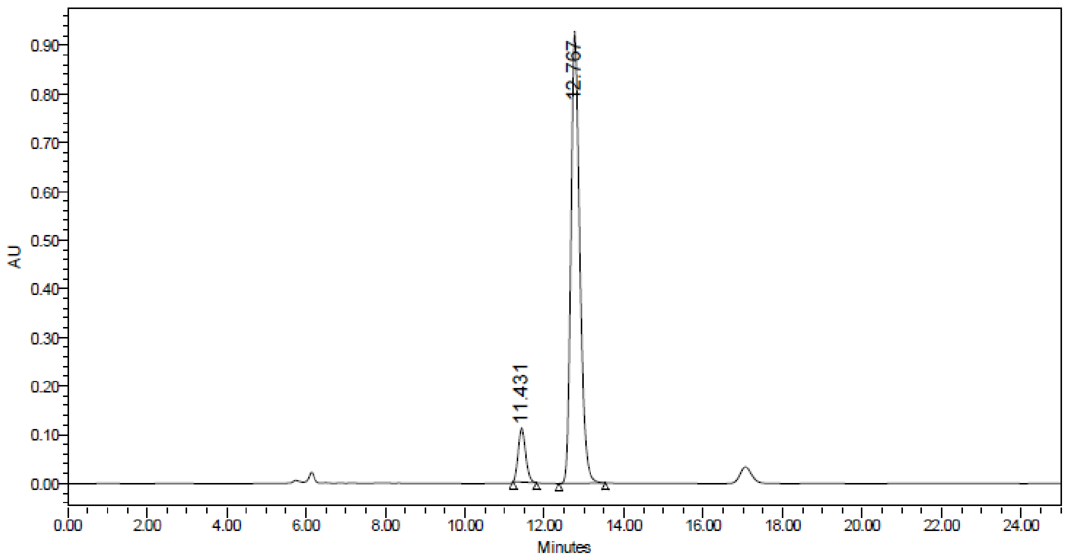

	RT (min)	Area (µV*sec)	% Area	Height (µV)	% Height
1	11.431	1458444	9.09	111225	10.70
2	12.767	14594653	90.91	928123	89.30

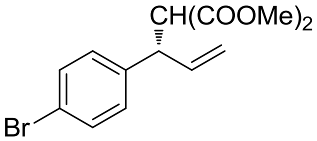

6h

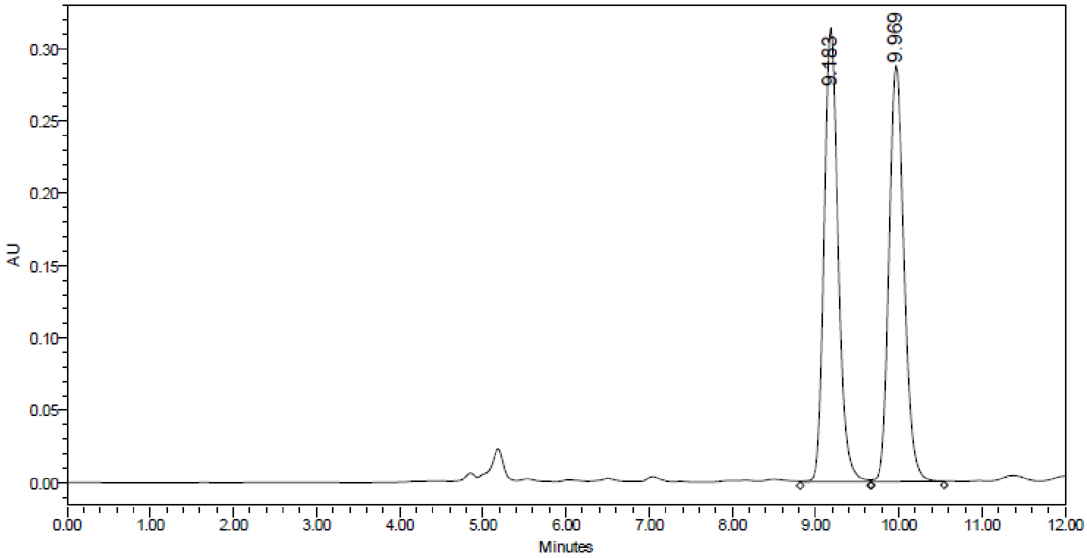

	RT (min)	Area (µV*sec)	% Area	Height (µV)	% Height
1	9.183	3684189	50.29	313703	52.14
2	9.969	3641751	49.71	287951	47.86

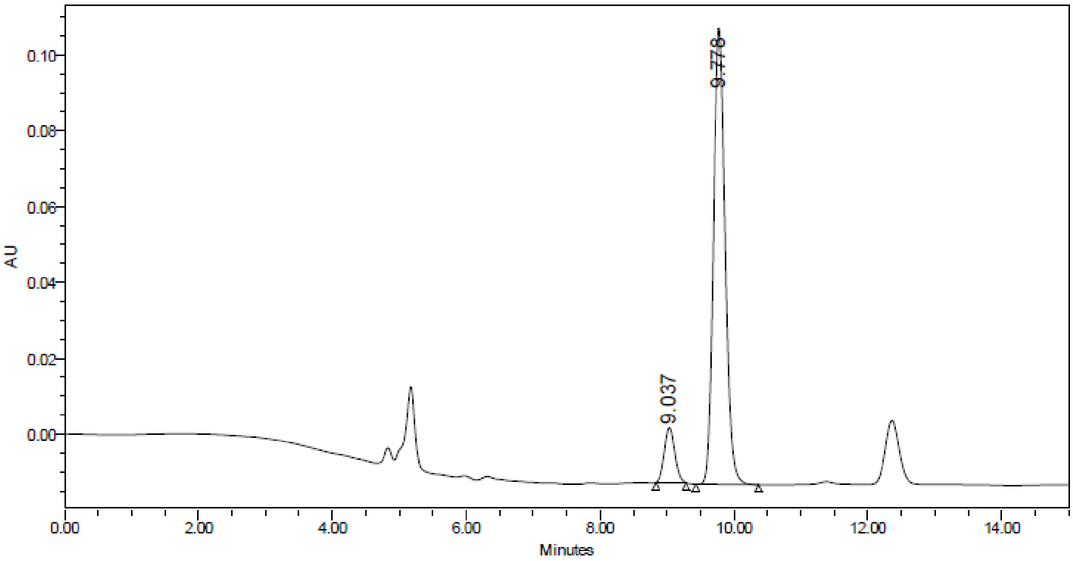

	RT (min)	Area (µV*sec)	% Area	Height (µV)	% Height
1	9.037	155756	9.62	14561	10.78
2	9.778	1464036	90.38	120464	89.22

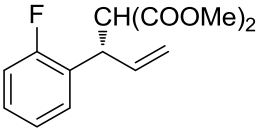

6i

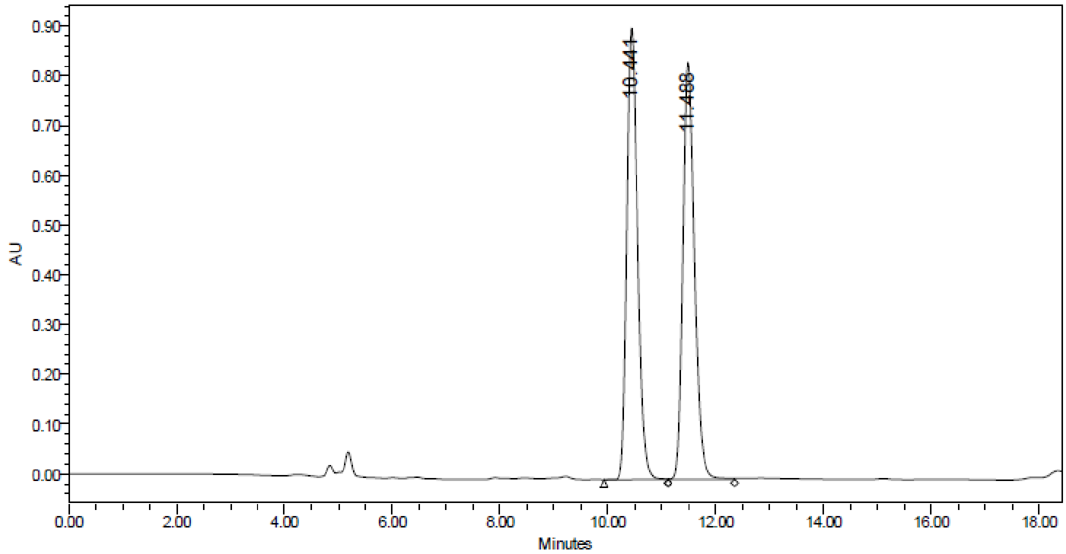

	RT (min)	Area (µV*sec)	% Area	Height (µV)	% Height
1	10.441	12564478	49.88	910416	52.05
2	11.488	12626680	50.12	838856	47.95

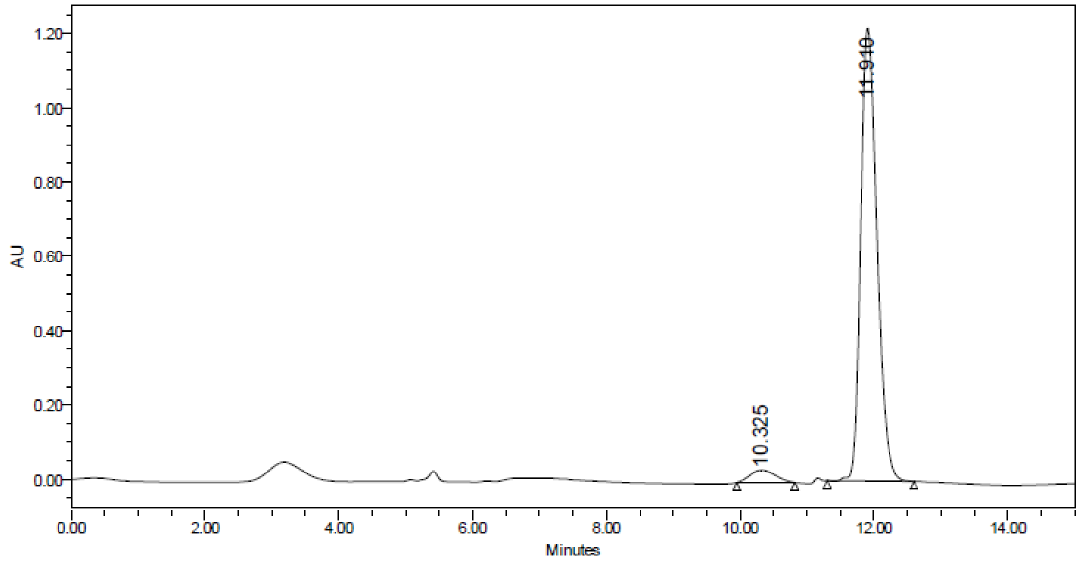

	RT (min)	Area (µV*sec)	% Area	Height (µV)	% Height
1	10.325	885124	4.11	33392	2.66
2	11.910	20653228	95.89	1221772	97.34

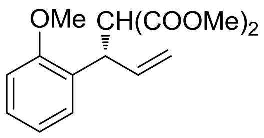

6j

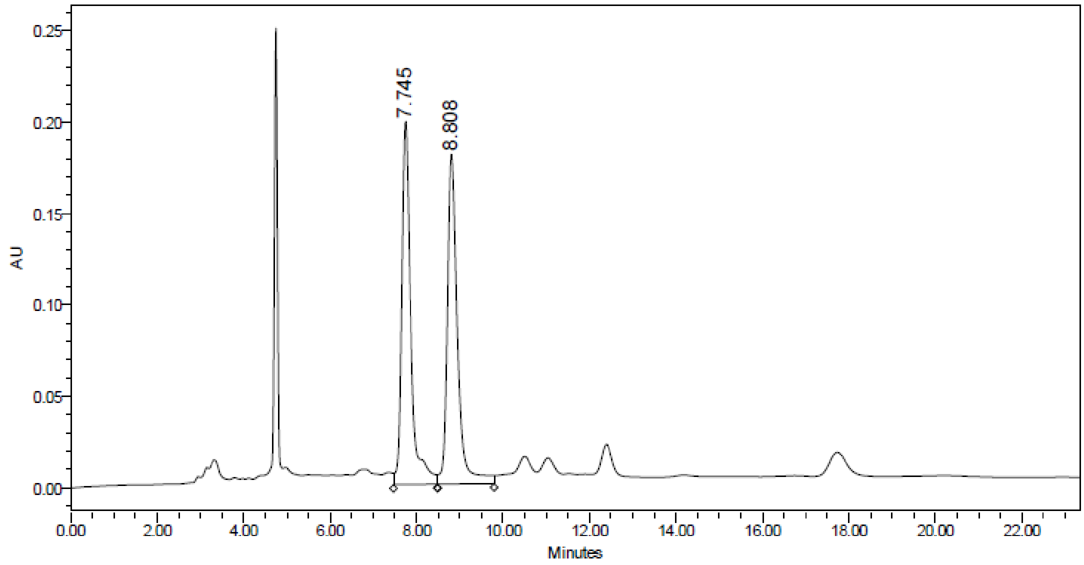

	RT (min)	Area (µV*sec)	% Area	Height (µV)	% Height
1	7.745	2930126	49.37	198886	52.36
2	8.808	3004958	50.63	180981	47.64

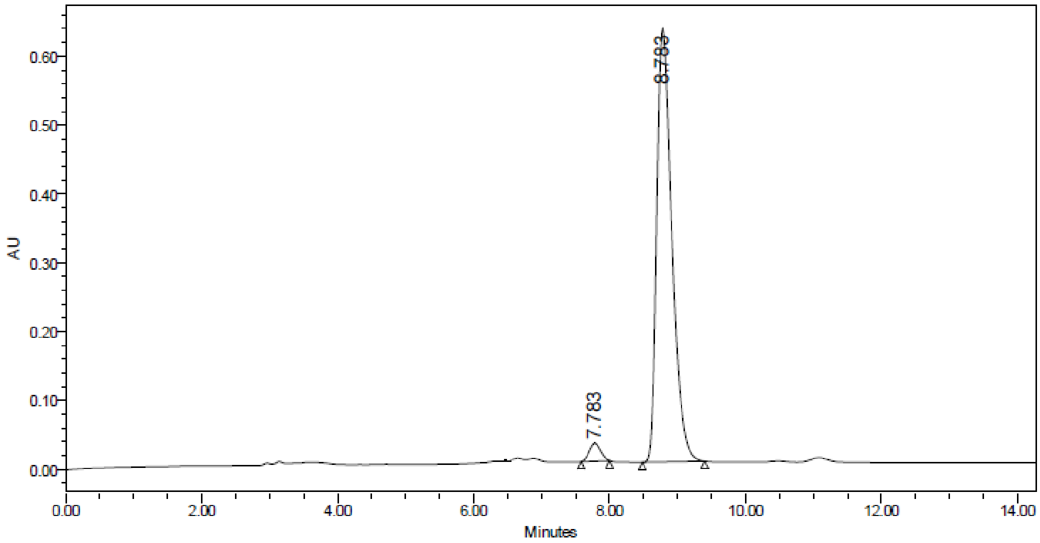

	RT (min)	Area (µV*sec)	% Area	Height (µV)	% Height
1	7.783	301969	3.02	25945	3.95
2	8.783	9712770	96.98	630885	96.05

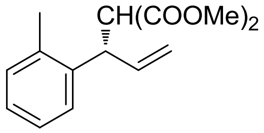

6k

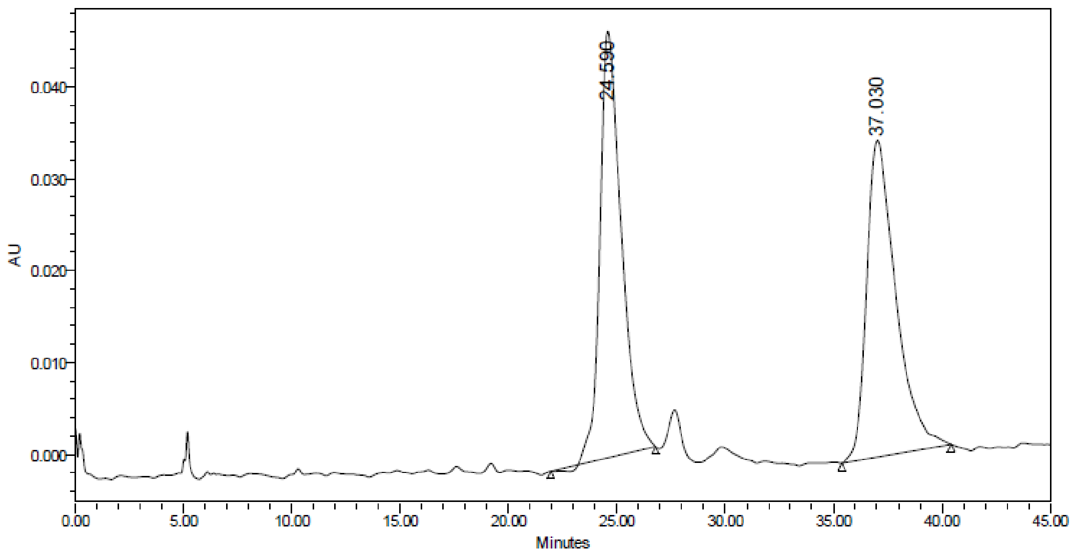

	RT (min)	Area (µV*sec)	% Area	Height (µV)	% Height
1	24.590	3235742	49.98	46381	57.39
2	37.030	3237897	50.02	34439	42.61

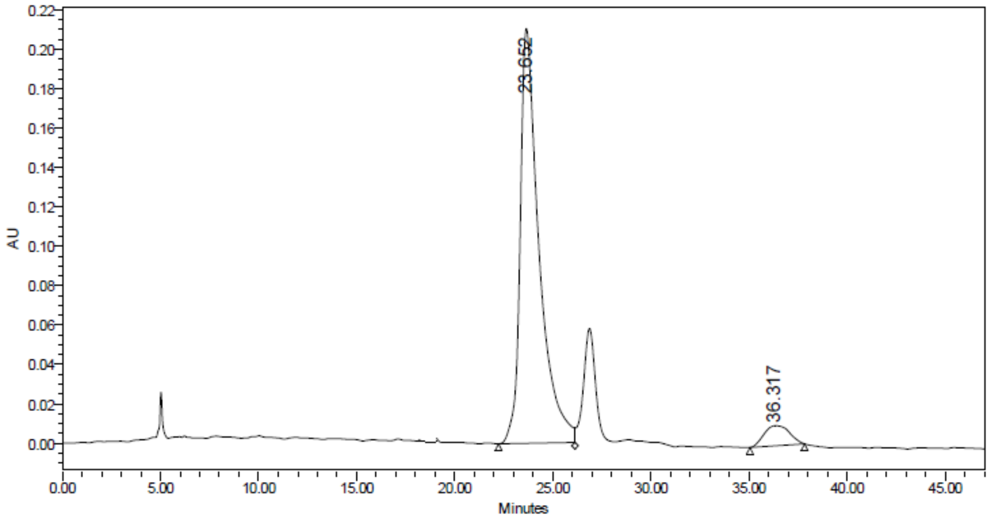

	RT (min)	Area (µV*sec)	% Area	Height (µV)	% Height
1	23.652	14103651	93.92	210550	95.38
2	36.317	912731	6.08	10191	4.62

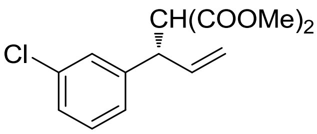

6l
